# Observational study of antibiotic prescribing patterns by age and sex in primary care in England: why we need to take this variation into account to evaluate antibiotic stewardship and predict AMR variation

**DOI:** 10.1093/jacamr/dlae210

**Published:** 2025-02-07

**Authors:** Naomi R Waterlow, Tom Ashfield, Gwenan M Knight

**Affiliations:** Centre for Mathematical Modelling of Infectious Diseases, Department of Infectious Disease Epidemiology, London School of Hygiene and Tropical Medicine, London, UK; AMR Centre, London School of Hygiene and Tropical Medicine, London, UK; The Signpost, Winchester, UK; Centre for Mathematical Modelling of Infectious Diseases, Department of Infectious Disease Epidemiology, London School of Hygiene and Tropical Medicine, London, UK; AMR Centre, London School of Hygiene and Tropical Medicine, London, UK

## Abstract

**Background:**

The drivers of antimicrobial resistance (AMR) likely vary substantially by different demographics. However, few complete, open, national detailed data exist on how antibiotic use (ABU) varies by both age and sex. Here, we aimed to describe the variation in ABU and consider how these age- and sex-specific patterns influence targets and methods for AMR control.

**Methods:**

Prescriptions of all antibiotics from general practices in England for 2015–23 disaggregated by 5 year age bands and sex were analysed at the national and Integrated Care Board (ICB) level. A descriptive analysis of the relative rates of ABU by age and sex was conducted, followed by an evaluation of comparison metrics of prescription levels between regions. ABU by age and sex were compared with global AWaRe targets, and ABU by age-group was correlated against influenza vaccination introduction, to evaluate the impact of such vaccines on ABU.

**Results:**

From a total of 249 578 795 prescriptions (across 9 years), 63% were given to women and the most prescribed were amoxicillin, nitrofurantoin and flucloxacillin sodium. Prescriptions per 100 000 population varied substantially across sex, age, geographical region, season, year, COVID-19 pandemic period and drug. Most antibiotics were prescribed more to women across most age bands (84% of antibiotics had more prescriptions to females across 50% of age bands). We show how this variation requires a more nuanced approach to comparing ABU across geographies and highlight that AWaRe targets are not met uniformly [prescribing in men aged 11–20 does not fulfil the Access, Watch and Reserve (AWaRe) 80% Access target]. We also show the impact on ABU of time-sensitive interruptions (38% of antibiotics showed a seasonal pattern in the absence of disruptions), including differential age-targeted influenza vaccination, COVID-19 restrictions and a shortage of amoxicillin due to a group A *Streptococcus* outbreak. However, we found few open data to link age- and sex-specific ABU to relevant AMR.

**Conclusions:**

These detailed differences in ABU across England suggest that there should be large variation in AMR burden by age and sex. Linkage of this ABU data with similarly detailed open-access AMR data is now needed for better intervention design.

## Introduction

The global public health priority that is antimicrobial resistance (AMR) is driven by a population-specific combination of antibiotic selection and transmission. Tackling AMR is guided by many global targets that focus on the reduction of antibacterial use (ABU). One such target committed to in the UN General Assembly is that at least 70% of total antibiotic consumption should be within the Access group of Access, Watch and Reserve (AWaRe) antibiotics,^1,2^ higher than a previous 60% WHO target. More ambitiously, it has been proposed that 80% or more of oral antibiotic use could be Access.^3,4^ To achieve these and other targets without affecting patient care, it will be key to produce nuanced information about ABU across different subpopulations. An important question is therefore how does ABU vary across age and sex and how will this affect how targets are reached/implemented?

Most antibiotics are given outside of hospital settings,^5,6^ and previous studies have suggested that this primary care antibiotic use (and hence exposure) varies substantially by age and by sex. A systematic review of primary care prescribing in 2016 suggested that women in high-income settings receive a significantly higher number of prescriptions, particularly in the wide 16 to 54 years age band.^7^ This is supported by sub-national analyses^8–10^ from the UK—e.g. approximately twice as many antibiotic prescriptions were given to women in 2013–15, and 70% of all prescriptions after excluding antibiotics used to treat urinary tract infections (UTIs) were given to women.^8^ Antibiotic exposure rates are often ‘hockey-stick’ shaped across age, with high levels in young children and an exponential increase with age.^7,11^ This likely reflects different rates of infection in different age/sex sub-groups^12^ and hence some level of appropriate prescription variation. For example, women have higher rates of UTIs.^13^ These previous studies emphasized how prescription rates vary across primary care but lacked access to full national data by detailed age and sex disaggregation.

Antibiotic use also varies geographically at national and sub-national levels,^14–16^ and identifying outliers once patient composition is accounted for is vital for improving antibiotic stewardship^17,18^ and to achieve the aforementioned targets. To improve stewardship, various comparison metrics have been proposed such as the English Specific Therapeutic group Age-Sex Related Prescribing Unit (STAR-PU),^19^ which uses different prescribing weights in different ages and sexes to compare prescriptions fairly based on underlying patient population characteristics. However, this comparison metric has flaws such as the lack of accounting of comorbidities^18^ and the grouping of all antibiotics together masking variation in antibiotic use due to patient or GP-related factors,^20^ although variation in prescribing may be decreasing over time.^21^ A key question is the appropriateness of STAR-PU metrics given updated data with more demographic details.

To reduce ABU many age-specific interventions could be considered; for example, vaccination programmes are postulated to reduce both unnecessary and necessary antibiotic use.^22,23^ Evidence that such a reduction in antibiotic exposure leads to reduced resistance is mixed,^24^ but age- and sex-disaggregated ABU could be used to reveal where there is a link between vaccination and ABU, as well as ABU and resistance rates. However, few ecological open-access ABU or AMR data exist disaggregated by both age and sex. In one of the only detailed age and sex analyses, we have previously shown that AMR prevalence in bloodstream infections in Europe varies substantially by age, sex and bacteria-antibiotic combination.^25^

Here we used 9 years of open-access national data on all antibiotic prescriptions from primary care in England to explore how ABU varies by both age and sex, with a descriptive analysis. We then asked specific questions of (i) how appropriate is STAR-PU (the main method used in England) for identifying prescribing outliers? (ii) are ABU AWaRe targets being achieved in different demographic subgroups? (iii) by how much does age-based influenza vaccination impact ABU and (iv) can we link age- and sex-disaggregated ABU to AMR? The patterns we found show the importance of such nuanced data and emphasize the need for more disaggregated AMR data.

## Materials and methods

### Data

The number of primary care antibiotic prescriptions by 23 age bands and three genders in England and Wales was received through two Freedom of Information (FOI) requests (FOI-01671, FOI-01975)^26^ to the NHS Business Service Authority (NHSBSA) Information Services Data Warehouse, which holds information from the NHSBSA Prescription Services. The first FOI contained data at the general practice and Integrated Care Board (ICB) level for England and some of Wales. ICBs (of which there are 42 in England) are NHS organizations responsible for planning health services in their local population, working with local providers of NHS services to contribute to integrated care strategies.^27^ The second FOI was for total national English data only.

These data covered the 9 year period from April 2015 to December 2023 inclusive, by month, and included all medicines from the BNF Section 5.1: Antibacterial drugs.^28^ Throughout, we followed the BNF groupings of antibiotics. Datapoints relating to fewer than five items prescribed were redacted by NHSBSA for privacy reasons. The 23 age bands were ‘0–1’, ‘2–5’ and then 5 year age bands up to ‘105+’. The three gender classes included were ‘Male’, ‘Female’ and ‘Indeterminate’ (where information does not confirm either Male or Female). We used number of items prescribed in our analysis, so as to avoid double-counting of patients.^26^

The proportion of all prescriptions in the dataset prescribed to a patient of unknown gender or age band was 3.3%. We excluded these prescriptions from our analysis, although note that the frequency varied with time, with fewer unknowns over time (Appendix 1 and Figures S1 and S2, available as Supplementary data at *JAC-AMR* Online). We also excluded prescriptions given to individuals of ‘Indeterminate’ gender, due to the small numbers (6247 items, 0.0024%) and the antibiotics for which there were less than an average of 10 prescriptions per year (22 antibiotics; Appendix 1, Section 2). For our main analysis, we assumed all numbers of items that were unavailable due to small numbers had a value of 1 (from possible values of 1, 2, 3 or 4), although we tested this assumption with a range of sensitivity analyses on this number.

Population sizes by age were obtained from the Office for National Statistics (ONS), and mid-year estimates were used for each calendar year^29,30^ (Appendix 1, Section 3). We only included prescriptions for England in the analysis, and only those for 2023 were used for sub-national analyses, due to changes in the definitions of the ICBs. Data from NHSBSA provide ‘Gender’, but most other sources use ‘Sex’. We have assumed that we can match these two in this analysis.

Data collection and transmission rates were altered during the years of SARS-CoV-2 circulation and lockdowns, which we classified to be from March 2020 to June 2022 inclusive for England.

### Descriptive analysis

A descriptive analysis was conducted on the prescription data, focusing on the relative rates of prescriptions by age group and sex, as well as looking at overall prescription rates by antibiotic. Initially the rates of prescription by these demographic factors were compared individually, followed by an analysis by both age and sex together. Trends over time were compared, and antibiotics were defined as having an annual seasonal pattern in prescriptions (pre-COVID-19) or not. This was defined as having at least 2 months where prescriptions were 20% higher than the average number of prescriptions, averaged over years.

### Antibiotic usage metrics

Comparing prescriptions across different regions is complicated by different populations (e.g. an older population, a population with more comorbidities, etc.) being present in each region. To counteract this, age- and sex-specific population weightings are often calculated to allow for less biased comparisons. The STAR-PU weighting system is often used in England.^19^ We calculated an updated STAR-PU weighting of antibiotic prescriptions per age band and sex, with smaller age bands^19^ using data from 2023. These updated weightings, which we have named Updated Comparison Metric (UCM) weightings, were calculated using females aged 66–70 as baseline, chosen as this is a subset of the STAR-PU 65–74 female baseline and therefore provides the closest comparison. Our UCM weightings were then applied to the ICBs in 2023, using the same STAR-PU principles: the new UCM weightings were multiplied by the relevant populations in each ICB, and the total prescriptions per UCM population were used to compare ICBs. In addition, drug-family–based weightings were calculated using the same methodology (‘UCM-family’), but prescriptions from each drug family were used. For children aged up to 16 (the only age groups where there were sometimes no prescriptions) a value of 0.01 was used if there were no data in that age band, and limits were set of 0.01 to 100. The interpretation of these weightings is that on average, an individual in the relevant population subset receives X-weighting more antibiotics than the baseline population group.

### Antibiotic consumption targets

Drugs were classified into the AWaRe categories using a WHO reference table.^31^ Where drug names were not identical, these were manually matched (Appendix 1 and Table S1). The percentage of drugs in each AWaRe category was broken down by age group and sex, and evaluated over time.

### Influenza vaccination

Age bands eligible for NHS influenza vaccination each year were extracted from the annual UK Health Security Agency (UKHSA) reports,^32^ and these were compared with prescriptions of antibiotics used in respiratory tract infections (RTIs) (defined as all drugs in the BNF categories ‘cephalosporins and other beta-lactams’ and ‘penicillins’), excluding amoxicillin. Amoxicillin was excluded due to the very large number of prescriptions, and its use in many varieties of infection^33^ that may not be respiratory related, thereby masking the potential effect of vaccination. The influenza season was defined as 1 October to 30 March inclusive, based on annual circulation patterns of influenza recorded by the UKHSA.^34^ The number of prescriptions given to patients in age bands that matched closely to the vaccination groups was calculated where possible (0–1, 2–5, 5–10, 11–15, 50–65, 65+).

### AMR comparison

We searched for publicly available age- and sex-disaggregated AMR data and extracted the only national-level English data we could find. This was for *Staphylococcus aureus* bloodstream infection (BSI) data from UKHSA.^35^ The proportion of these BSIs due to *S. aureus* that were methicillin-resistant (MRSA) were compared with prescriptions of antibiotics in the BNF categories ‘cephalosporins and other beta-lactams’ and ‘penicillins’.

### Code

All code is available on GitHub (https://github.com/NaomiWaterlow/prescription_foi) and was conducted in R.^36^

### Sensitivity analysis

The main issue to explore in the data was the uncertainty in the redacted small numbers (‘*’) when the number of prescriptions was 1–4. In sensitivity analysis we explored changing our default value of 1 to 4 for (i) everyone, (ii) males only and (iii) children only (<20-year-olds) (Appendix 1 Section 7, Figures S4-S6).

## Results

### Prescription data

Over the 9 year period April 2015 to December 2023, 249 578 795 prescriptions of antibiotics were given in England. For the complete years (2016–23), the average was 0.48 (range over years of 0.43, 0.52) prescriptions per person per year. The most frequently prescribed drugs were amoxicillin, nitrofurantoin and flucloxacillin sodium (Table 1). Sixty-three percent (range over years: 63%, 64%) of drugs were prescribed to females, with 0.60 (range over years: 0.54, 0.65) per person per year versus 0.36 (range over years: 0.31, 0.40) in men. Thirteen percent (range over years: 10%, 15%) of prescriptions were given to <16-year-olds, and 35% (range over years: 33%, 37%) to those over 65 years. The highest overall prescription rate in both sexes was in the 86+ year group (Appendix 1 and Figure S3). COVID-19 mostly affected prescribing in those aged <16 years (Appendix 1 and Figure S3), mostly returning to pre-COVID-19 levels in the spring of 2021.

**Table 1.** Summary data on number of prescriptions for the 20 most prescribed antibiotics (see Appendix 1, Section 6 for all antibiotics) across the complete years of the data (2016–2023) unless otherwise indicated

Antibiotic name	Total prescriptions (number)	Mean prescriptions (annual number)	Percentage to females^a^	Top month^b^	Total number of prescriptions in			Top age band^c^ (2023)	
					2016	2019	2023	Females	Males
Amoxicillin	53 415 335	6 676 917	58	12	8 130 012	6 673 263	7 311 226	**0–1,2–5,86+**	**0–1,2–5,86+**
Nitrofurantoin	27 953 870	3 494 234	83	10	2 358 297	3 740 424	3 808 622	76**–**80,81**–**85,86+	76**–**80,81**–**85,86+
Flucloxacillin sodium	26 940 364	3 367 546	55	7	3 488 057	3 270 904	3 384 802	76**–**80,81**–**85,86+	76–80,81–85,86+
Doxycycline hyclate	22 868 632	2 858 579	60	12	2 415 657	2 772 514	3 804 564	76–80,81–85,86+	76–80,81–85,86+
Penicillin V	15 557 160	1 944 645	59	12	1 892 172	1 841 679	2 447 555	**2–5,6–10,16–20,**	**0–1,2–5,6–10**
Trimethoprim	14 439 590	1 804 949	75	1	3 087 875	1 488 922	1 428 569	76–80,81–85,86+	76–80,81–85,86+
Clarithromycin	14 417 677	1 802 210	63	1	2 077 261	1 845 315	1 743 846	76–80,81–85,86+	76–80,81–85,86+
Co-amoxiclav^d^	9 181 476	1 147 684	60	1	1 304 696	1 094 885	1 092 620	76–80,81–85,86+	76–80,81–85,86+
Lymecycline	7 938 074	992 259	58	3	1 054 631	1 032 182	861 423	**11–15,16–20,21–25**	**11–15,16–20,21–25**
Cefalexin	5 910 292	738 786	80	12	748 696	668 339	816 579	76–80,81–85,86+	76–80,81–85,86+
Azithromycin	5 877 890	734 736	57	12	612 414	748 885	805 700	71–75,76–80,81–85	76–80,81–85,86+
Metronidazole	4 156 192	519 524	78	3	599 382	520 332	474 629	**21–25,26–30,31–35**	76–80,81–85,86+
Ciprofloxacin	3 564 433	445 554	48	1	532 816	445 243	355 804	76–80,81–85,86+	76–80,81–85,86+
Erythromycin	3 397 046	424 631	66	1	709 522	394 861	286 163	76–80,81–85,86+	76–80,81–85,86+
Oxytetracycline	2 432 724	304 090	51	3	463 514	310 851	178 452	61–65,66–70,76–80	71–75,76–80,81–85
Pivmecillinam hydrochloride	1 790 378	223 797	79	10	108 361	223 030	301 447	76–80,81–85,86+	76–80,81–85,86+
Erythromycin ethylsuccinate	1 576 325	197 041	50	12	341 227	171 870	164 552	**0–1,2–5,6–10**	**0–1,2–5,6–10**
Co-trimoxazole^e^	1 417 031	177 129	44	12	129 051	173 722	228 051	66–70,76–80,81–85	76–80,81–85,86+
Methenamine hippurate	1 060 060	132 508	80	12	40 789	82 081	319 013	76–80,81–85,86+	76–80,81–85,86+
Rifaximin	593 273	74 159	41	12	33 589	71 036	109 205	56–60,61–65,66–70	56–60,61–65,66–70

^a^Underlining indicates more prescriptions are to females.

^b^‘Top’ month is the month with the most prescriptions of this antibiotic (1 = January), when summing across the whole dataset.

^c^‘Top age band (2023)’ is the age band in 2023 with the highest prescription rate per 100 000. Bold font indicates relatively more prescriptions to younger individuals versus top age bands (i.e. 55 years old or more).

^d^Co-amoxiclav is amoxicillin and clavulanic acid.

^e^Co-trimoxazole is trimethoprim and sulfamethoxazole.

**Figure 1.**
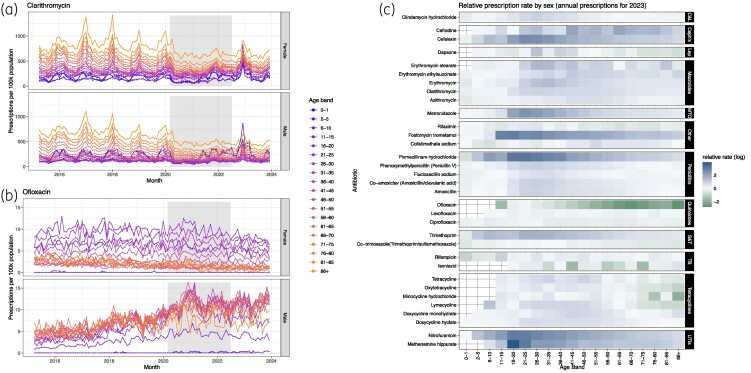
Variation in antibiotic prescribing by age and sex with examples and overall comparison. Example prescription rate per 100 000 population for (a) clarithromycin and (b) ofloxacin. Colours indicate age bands, facets indicate sex. Grey band indicates years of COVID-19 interventions. See Appendix 2 for all antibiotics. (c) Relative prescription rate by sex for prescribed medicines in 2023. Colours represent the log of the ratio, with blue indicating higher prescriptions in women, and green indicating higher prescriptions in men. Drugs are ordered by BNF classification. C&L, clindamycin and lincomycin; Cephs, cephalosporins and other β-lactams ; Lep, antileprotic drugs; MTO, metronidazole, tinidazole and ornidazole ; S&T, sulphonamides and trimethoprim; TB, antituberculosis drugs; UTIs, urinary tract infections.

The top 20 prescribed antibiotics, totalling 98% of prescriptions (Table 1), had annual prescription totals varying from 74 159 to over 6.5 million prescriptions, with most being prescribed more commonly to females (17/20) and most often in December (9/20) or January (5/20). The top 20 antibiotics prescribed more to men were ciprofloxacin, co-trimoxazole and rifaximin. Just over half of the top 20 prescribed antibiotics had fewer prescriptions in 2023 than in 2016 (11/20), with 61% of antibiotics having at least a 10% reduction in prescriptions between 2019 and 2023, although some had sex-based time variation (e.g. ofloxacin, Figure 1b). Most of the top 20 antibiotics (15/20) had the highest rates in older ages (>55-year-olds) in 2023.Twenty-eight antibiotics (38%) showed an annual seasonal pattern (Figure 1a and Appendix 2) in prescriptions pre-COVID-19. These seasonal antibiotics all had a decrease in prescriptions during the COVID-19 period, followed by a spike in late 2022 (Appendix 2) correlating with a group A *Streptococcus* outbreak and amoxicillin shortage.^37^

For most age bands in 2023 we observed higher levels of prescription to females compared with males (Figure 1c) (84% of antibiotics had more prescriptions to females in at least 50% of age bands, with 54% in at least 75% of age bands, and 24% in at least 90% of age bands). Notable exceptions with higher prescriptions to males in 2023 were quinolones (especially ciprofloxacin) and isoniazid, and in age group 66+, minocycline hydrochloride (Figure 1c). For some ages, in 2023, the sex that received the most prescriptions changed with age [e.g. colistimethate sodium, azithromycin and rifampicin had higher prescriptions in males in children but higher prescription rates in females in adults (Figure 1c)].

### Antibiotic usage metric

**Figure 2.**
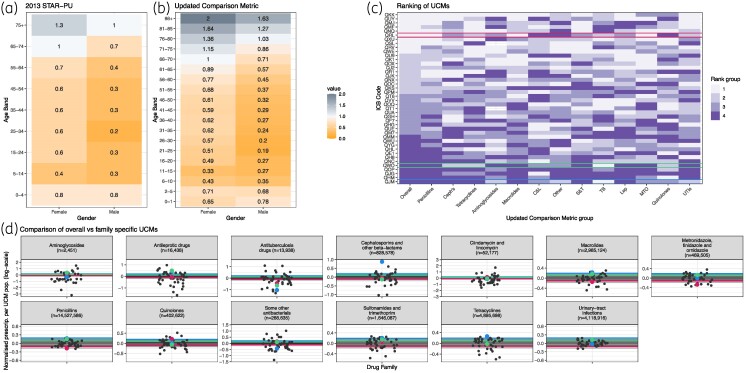
(a) 2013 STAR-PU values using England data. (b) Updated Comparison Metric (UCM) using 2023 English data and more disaggregated age bands. Grey = high value, yellow = low value, centred around the baseline: females, 66–70 (white). (c) Ranking of UCMs across English ICBs, grouped by colour into quarters: rank 1–10 (top 10 highest prescribers, grouped into category ‘1’), 11–20 (next 10 highest prescribers, grouped into category ‘2’), 21–30 (next 10 highest prescribers, grouped into category ‘3’) and 30–42 (lowest 12 prescribers, grouped into category ‘4’). Therefore, higher rank indicates higher prescribing. The ‘Overall’ column shows the ranking of ICBs using our UCM, and the other columns are ranked by UCM-family. (d) Normalized prescriptions per English ICB and antibiotic (facet) on the log-scale for overall UCM (lines, same on each facet) and UCM families (dots). This allows for comparison of the overall UCM with the variation in UCM family, indicating how much the overall UCM represents the diversity that is present when considering the UCM families. In (c) and (d) the same three ICBs are highlighted with colour (blue, green and pink). *n* indicates the total number of prescriptions across all ICBs. C&L, clindamycin and lincomycin; Cephs, cephalosporins and other β-lactams ; Lep, antileprotic drugs; MTO, metronidazole, tinidazole and ornidazole ; S&T, sulphonamides and trimethoprim; TB, antituberculosis drugs; UTIs, urinary tract infections.

Our UCM with smaller age bands than STAR-PU (Figure 2b) resulted in more extreme values across age bands, ranging from 0.19 to 2 in our UCM, compared with a range of 0.2–1.3 in the 2013 STAR-PU, with particularly higher values in older males (Figure 2a versus 2b). Values by BNF drug value varied considerably by age and sex (‘UCM-family’, Appendix 1, Section 7, Figures S7-S9).

Our new UCM ranking of ICBs (first column Figure 2c, lines in Figure 2d) changed substantially when applying the UCM-family (Figure 2c, points in Figure 2d). For example, ICB QRL ranks as a high prescriber with the new UCM (sixth overall, Figure 2c, red in Figure 2c and d) but a low prescriber of quinolones (38th overall, Appendix 1 and Figure S10). The range in UCM-family ranking decreased with more prescriptions (Figure 2d).

### Antibiotic consumption targets

**Figure 3.**
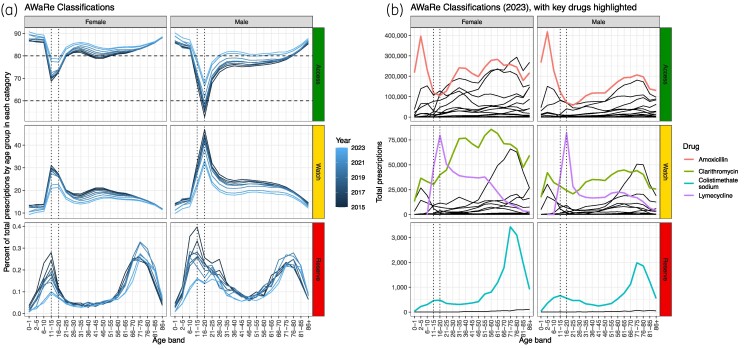
(a) Percentage of total prescriptions in each of the AWaRe categories (row), by age band, sex (column) and year (colour). Dashed horizontal lines represent targets of 60% and 80% in the Access category, and dotted vertical lines are included for comparison across graphs. Note the difference in *y*-axis ranges. (b) Total prescriptions in 2023 by AWaRe categories (row), age band and sex (columns). Key contributing drugs are highlighted by colour and vertical dotted lines are included for comparison across graphs.

The WHO 60% ‘Access’ prescribing target has been reached in England: 84% of primary care prescriptions were ‘Access’ in 2023. However, the more ambitious 80% level, and the 70% UN General Assembly target are not being met in males aged 11–20, largely driven by a spike in prescriptions for lymecycline (Figure 3b), commonly prescribed for acne.^38^ The main Reserve drug prescribed by GPs in 2023 was colistimethate sodium, which must be given by injection or as a nebulizer as it is not given orally,^39^ likely under the direction of tertiary care, potentially for cystic fibrosis patients.^40,41^ The percentage difference in proportion of antibiotics prescribed that were ‘Access’ between the lowest and highest prescribing ICBs varied by age and sex, with the smallest difference across ICBs being 5.3% (females, aged 6–10) and the largest difference being 13.4% (males, aged 16–20) (Appendix 1 and Figure S11).

### Influenza vaccination

**Figure 4.**
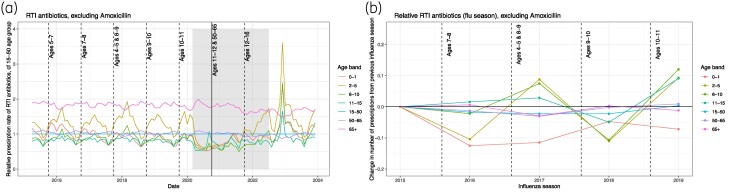
(a) Age band–specific prescription rates of RTI antibiotics (penicillins, cephalosporins and other β-lactams) relative to the rate in age band 15–50. See Appendix 1, Section 11 for analysis with amoxicillin. Vertical lines indicate the year of introduction of vaccination in the labelled age band, and the COVID-19 years are shaded grey. (b) Proportional change in the number of RTI prescriptions (excluding amoxicillin) from the influenza season of the previous year by age band. Vertical lines indicate the introduction of vaccinations in the named age bands.

Comparing the relative rate of prescriptions commonly given for (suspected) RTIs before and after influenza vaccination programmes (Figure 4a without amoxicillin; Appendix 1, Section 11 with amoxicillin) shows no clear impact. For example, compared with ages 15–50, RTI relative prescription rates declined by just under 5% in age band 6–10 following the introduction of vaccination in 7–8 year-olds (Figure 4a and b). However, in this same period the prescriptions in age band 0–1 decreased by over 10%. There was a relative increase in prescriptions in all ages except older adults when vaccination was introduced in 4–5-year-olds and 8–9-year-olds. Vaccination of 50–65-year-olds would likely have the largest impact (more people), but is hard to interpret, as this occurred during the COVID-19 years. No decrease in prescriptions in this age band compared with the other age bands was identified (Figure 4a).

### Impact on AMR

We found few age- and sex-disaggregated AMR data. As might be expected, comparison of this community AMU data with data on the hospital-associated pathogen MRSA found no clear link (Appendix 1, Section 12).

### Sensitivity analyses on data availability

The majority of age bands across antibiotics showed higher levels of prescription in females compared with males in all sensitivity analysis. When the hidden low prescription numbers (*s) were all set to the maximum possible value (4 instead of 1, equating to 0.13% instead of 0.033% of all prescriptions), 85% of antibiotics had more prescriptions to females in at least 50% of age bands, 55% in at least 75% of age bands, and 27% in at least 90% of age bands. The sensitivity analysis with the higher value (4) in males only or children only had highly similar levels of 78%/55%/18% and 85%/55%/24%, respectively (Appendix 1, Section 7).

## Discussion

We report here, to our knowledge, the first descriptive analysis of national data on all antibiotic prescriptions in primary care in England disaggregated by both 5 year age bands and sex. This unprecedented level of detail highlights huge variation and will be vital in bringing a more nuanced approach to ABU and hence AMR control.

Whereas most antibiotics (15/20 of most prescribed antibiotics) had higher prescription rates in older ages (ages 55+), a substantial proportion had higher prescription rates in children or younger adults. Matching previous reporting,^7–10^ women were prescribed more antibiotics, but this pattern was not uniform across age bands, and the patterns seen would have been hidden if using only three age groupings. Higher prescribing in women is likely linked to both infection syndrome variation and to behavioural differences in consultation rates.^8,42–45^ We saw clear syndrome-linked prescribing: higher levels of UTI prescribing (nitrofurantoin and trimethoprim^46,47^) in women and higher levels of prescribing linked to prostate infection or gonorrhoea (ofloxacin)^28^ in men. Quantifying this age and sex variation provides a baseline for intervention targeting and may help assess prescription appropriateness.

Most antibiotics (∼60%) did not have a seasonal pattern of exposure. Those that did had a lower prescribing rate during COVID-19, likely reflecting that antibiotics prescribed seasonally are given for infections that are dependent on seasonal changes in transmission (schools/time indoors), or linked to other infections (e.g. respiratory viruses^48,49^). Supporting this theory is the rapid increase in prescribing at the end of 2022 that coincided with a group A *Streptococcus* outbreak and a wide increase in prevalence of respiratory virus infections linked to ‘re-opening’.^50^ How this could drive seasonality in AMR needs untangling. Other studies have found a similar decrease in prescribing during COVID-19 in primary care in sub-national data in the UK,^51,52^ mostly driven by prescribing in children. However, the prescription rates largely returned to their previous trends following the pandemic and the group A *Streptococcus* outbreak, indicating that the pandemic had little impact on long-term prescribing.

These data allow for more subtle analysis of prescribing outliers. We found that STAR-PU likely underestimates prescribing to older adults, especially males, and that a metric split by antibiotic family would better identify places that are prescribing outliers. This analysis strengthens the case for updated STAR-PUs.^53^

We found that England is exceeding the UN General Assembly (UNGA) ‘70% Access’ targets for antibiotic use, and likely has been since 2018, like other European settings.^54^ However, these data show the utility of knowing the variation by detailed age bands and sex: males aged 11–20, as a result of high levels of prescribing of the Watch antibiotic lymecycline, likely for acne,^38^ had less than 60% Access in 2015, rising to 70% in 2023. Although also spiking in females, due to the higher overall prescribing in women, the proportion Access in women remains above 70%.

Interventions to reduce antibiotic prescribing, including the Quality Premium, which is an incentive system for encouraging healthcare providers to focus on national priorities, appear generally to be working in England, with most antibiotics (61%) having at least a 10% reduction in prescriptions between 2019 and 2023.^55,56^ Disaggregating by age and sex reveals subgroups where changes in prescribing practice could be further targeted; e.g. ofloxacin use has increased in men since 2016, potentially linked to rises in gonorrhoea.^57^ Interestingly, a large reduction in prescriptions is evidently not driven by more ages being eligible for influenza vaccination: despite roughly 50% vaccine coverage we saw little prescription reduction in that influenza season in the age group vaccinated nor in linked age groups known to benefit from influenza vaccination of children (e.g. 65+  age band).^58,59^ These results are lower than those previously reported (e.g. 29% reduction in antibiotic prescribing in one systematic review^22^) potentially due to our grouping antibiotics together, using wider age bands (to match vaccine targets) and not considering influenza complexity (e.g. strain variation). The impact of influenza vaccinations may also be higher in other non-UK settings.

When considering AMR selection, we found few open-access data and no obvious link between the age- and sex-disaggregated exposures of β-lactam antibiotics in primary care and higher MRSA prevalence in BSIs in England. However, MRSA is often a hospital-associated pathogen,^60^ and BSIs are usually treated in hospitals, hence a lack of a strong link might have been expected but not no link. More broadly, we clearly see that women receive more antibiotics in primary care, which is at odds with higher male resistance rates in our previous research in BSIs across Europe^25^ and available UK data.^35^ Such patterns point to unexplained complexity in the pathways to resistance acquisition and potential intervention targets such as increased age- or even sex-based prescribing recommendations. Untangling the link from ABU to AMR will require further age- and sex-disaggregated data but also information on other covariables such as patient ethnicity, deprivation level and comorbidities.^18,61^

Although the data used here are unparalleled in their detail and completeness, more patient data were available at later time points (a total of 3.3% of prescriptions were excluded). This led us to focus on the more complete 2023 data and we had to use sensitivity analyses to explore the hidden low numbers of prescriptions. Our data improve upon previous studies, which have banded either patient age (in 10+ age bands), not considered disaggregation by sex, or only focused on a subset of patient diagnoses.^7–9^ Moreover, much work in the UK is not all national data, using either only a subset of general practices,^8,9,61,62^ which although designed to be representative may miss subtle geographical variation, or incomplete market coverage (e.g. the company IQVIA has 89% market coverage in the UK^54^). However, here we only had access to prescription data, with no measure of the propensity to take the prescribed medicine and whether this varies by age, sex, time or drug, no linked diagnosis or reason for prescribing, and only ‘Items’, rather than a more specific quantification, such as DDDs or grams.^63^ This is important as dose and duration of exposure are potentially relevant to the development of resistance.^64–66^

The generalizability of this analysis to other locations is likely low (except in related geographies, e.g. Wales, Scotland), as the results may not be applicable to countries with different prescribing guidelines and healthcare systems (e.g. US insurance-based prescribing,^67^ or over-the-counter availability in low- and middle-income countries^68^). However, many of the underlying infection syndromes and behaviour patterns may exist, and hence our findings add to the increasing evidence that as selection pressures vary so substantially we need disaggregated data by age and sex to optimally intervene against AMR.

Our analysis highlights the possibilities of using FOI requests in research. Future researchers could request additional UK data, e.g. at the general practice level over larger time periods to avoid the issue of small numbers, as well as exploring requests for further (ideally linked) data that could help answer questions on health inequality such as socio-economic status and secondary healthcare infrastructure.^69^

Overall, by showing wide antibiotic prescription variation across age and sex this research has demonstrated the need for a nuanced approach when exploring and understanding trends in ABU. Understanding how this affects the global public health problem that is AMR can only be done with matched age- and sex-disaggregated AMR data otherwise the impact of ABU targets, ageing populations and future interventions will be very hard to determine.

## Supplementary Material

dlae210_Supplementary_Data

## Supplementary data

Figures S1–S3, S10 and S11, Table S1, and Appendices S1 and S2 are available as Supplementary data at *JAC-AMR* Online.
