# Supplementary material for: Observational study of antibiotic prescribing patterns by age and sex in primary care in England: why we need to take this variation into account to evaluate antibiotic stewardship and predict AMR variation
Source: JAC Antimicrob Resist. 2025 Feb 7;7(1):dlae210. doi: 10.1093/jacamr/dlae210 (PMC11803082; doi:10.1093/jacamr/dlae210)
Supplement: dlae210_Supplementary_Data [file dlae210_supplementary_data.zip › Appendix2.pdf]

# Appendix 2

Naomi Waterlow,Gwen Knight

2024-11-08

## Paper: Prescriptions of antibiotics in England

## Antibiotic specific results

This Appendix contains figures looking at each antibiotic by age band, sex and time.

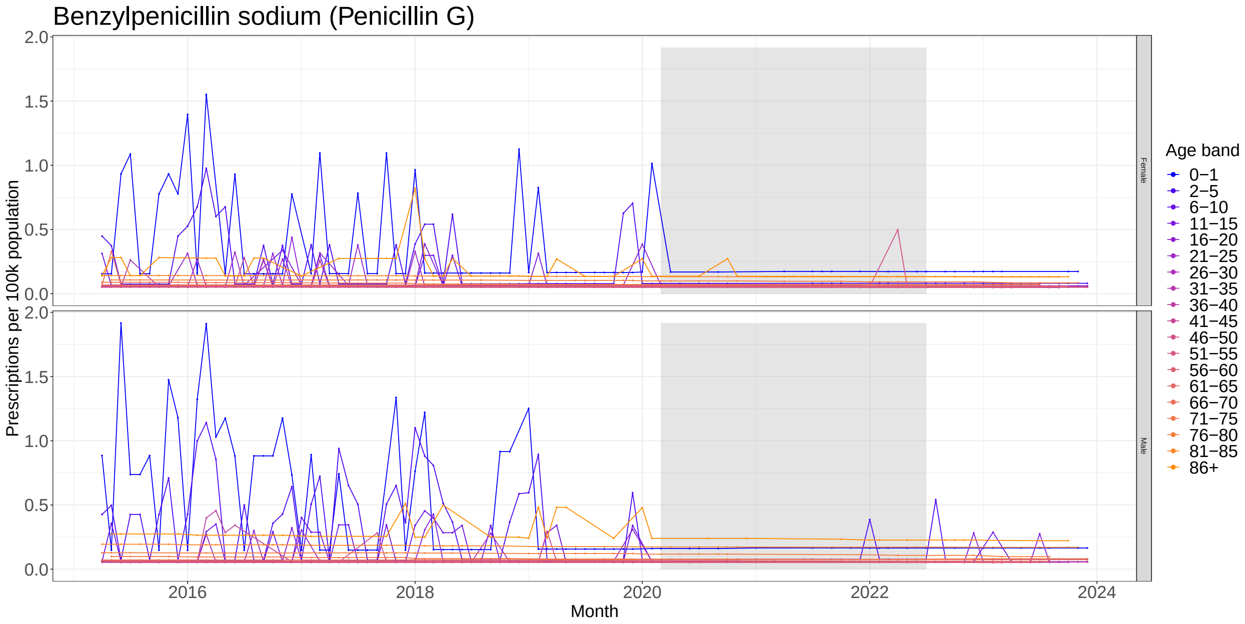


Figure S1: Prescription rate per 100'000 population for Benzylpenicillin sodium (Penicillin G) . Colours indicate age groups, facets indicate sex. Grey shading indicates years of Covid-19 interventions

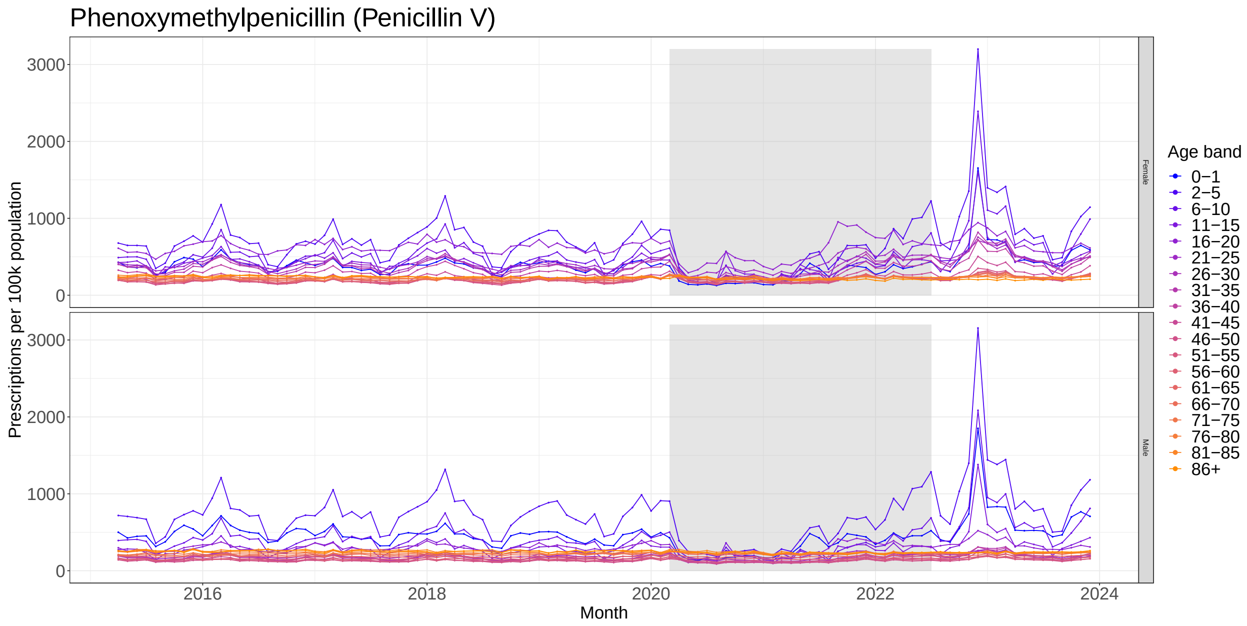


Figure S2: Prescription rate per 100’000 population for Phenoxymethylpenicillin (Penicillin V) . Colours indicate age groups, facets indicate sex. Grey shading indicates years of Covid-19 interventions

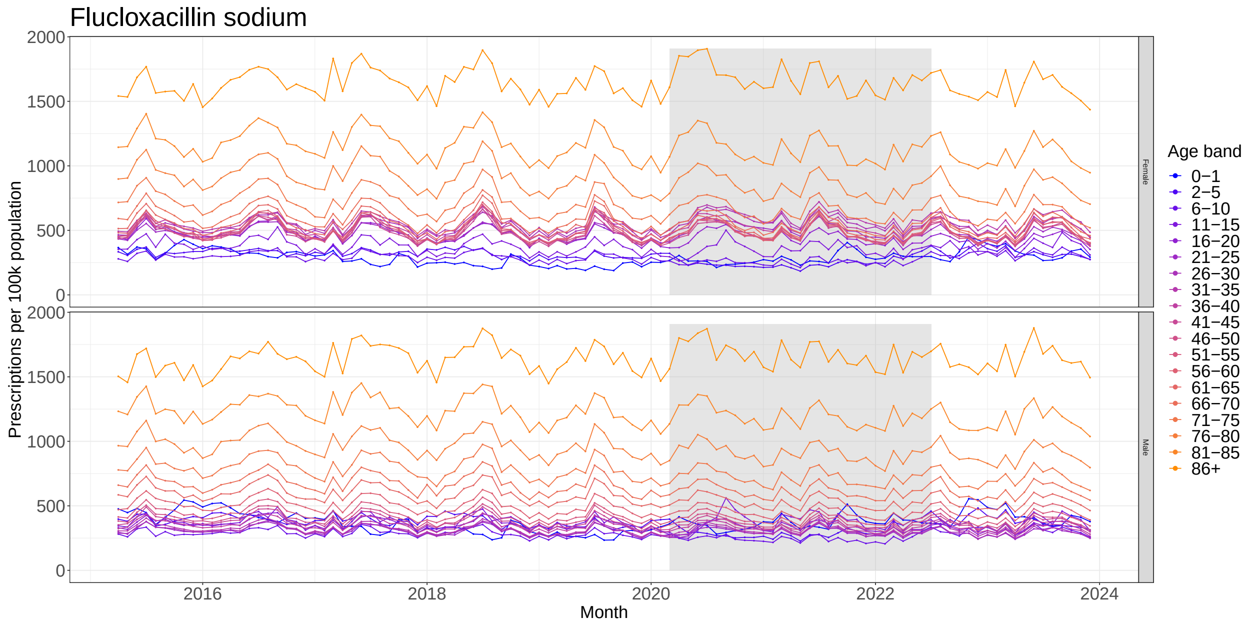


Figure S3: Prescription rate per 100’000 population for Flucloxacillin sodium . Colours indicate age groups, facets indicate sex. Grey shading indicates years of Covid-19 interventions

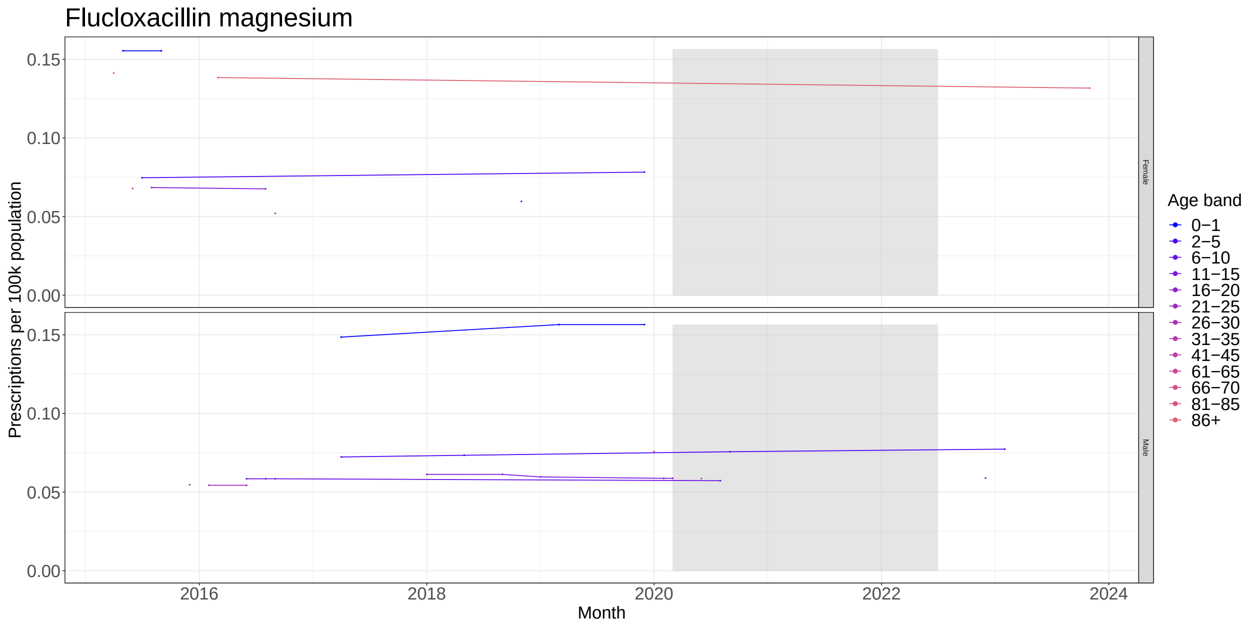


Figure S4: Prescription rate per 100’000 population for Flucloxacillin magnesium . Colours indicate age groups, facets indicate sex. Grey shading indicates years of Covid-19 interventions

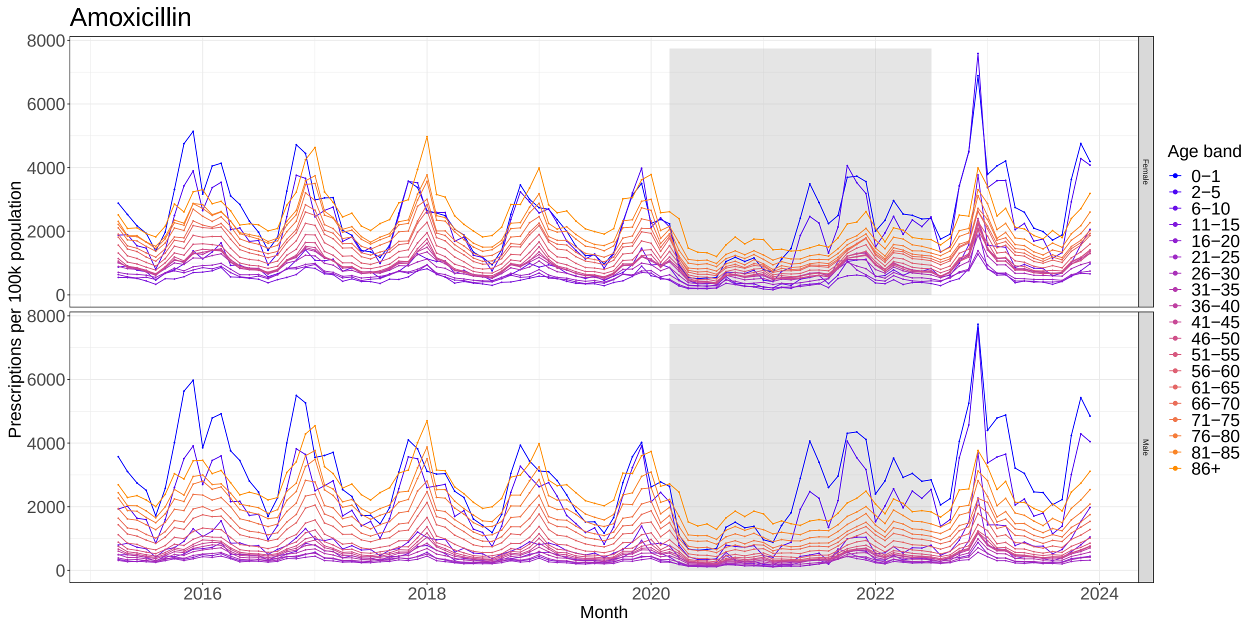


Figure S5: Prescription rate per 100’000 population for Amoxicillin . Colours indicate age groups, facets indicate sex. Grey shading indicates years of Covid-19 interventions

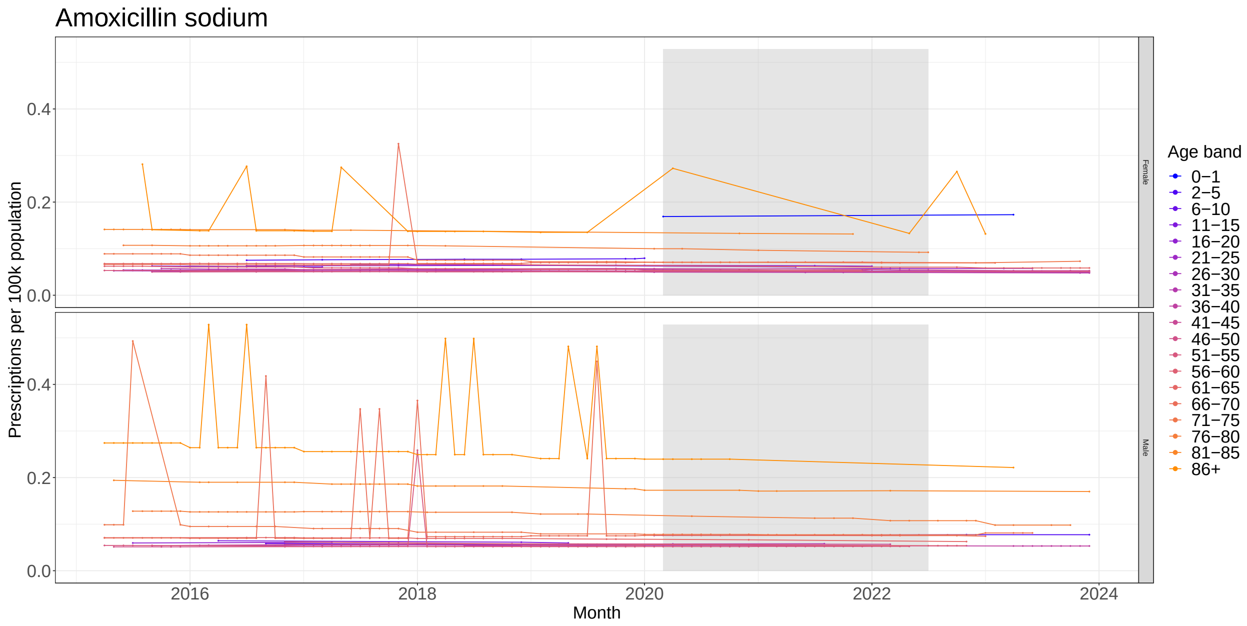


Figure S6: Prescription rate per 100'000 population for Amoxicillin sodium . Colours indicate age groups, facets indicate sex. Grey shading indicates years of Covid-19 interventions

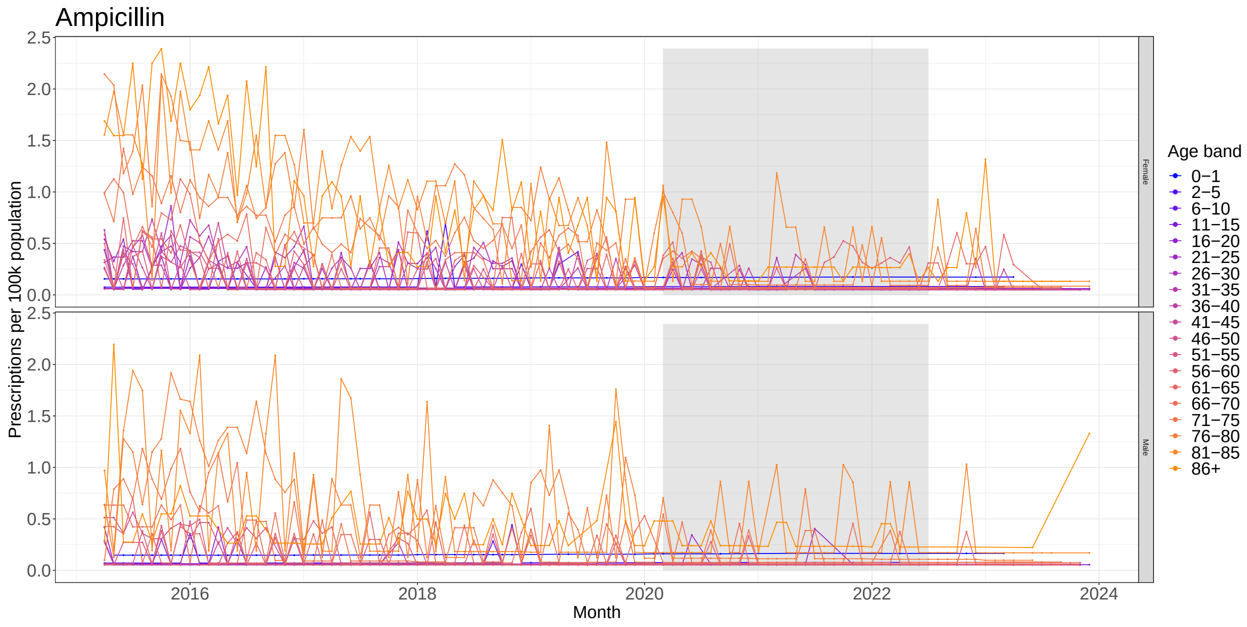


Figure S7: Prescription rate per 100'000 population for Ampicillin . Colours indicate age groups, facets indicate sex. Grey shading indicates years of Covid-19 interventions

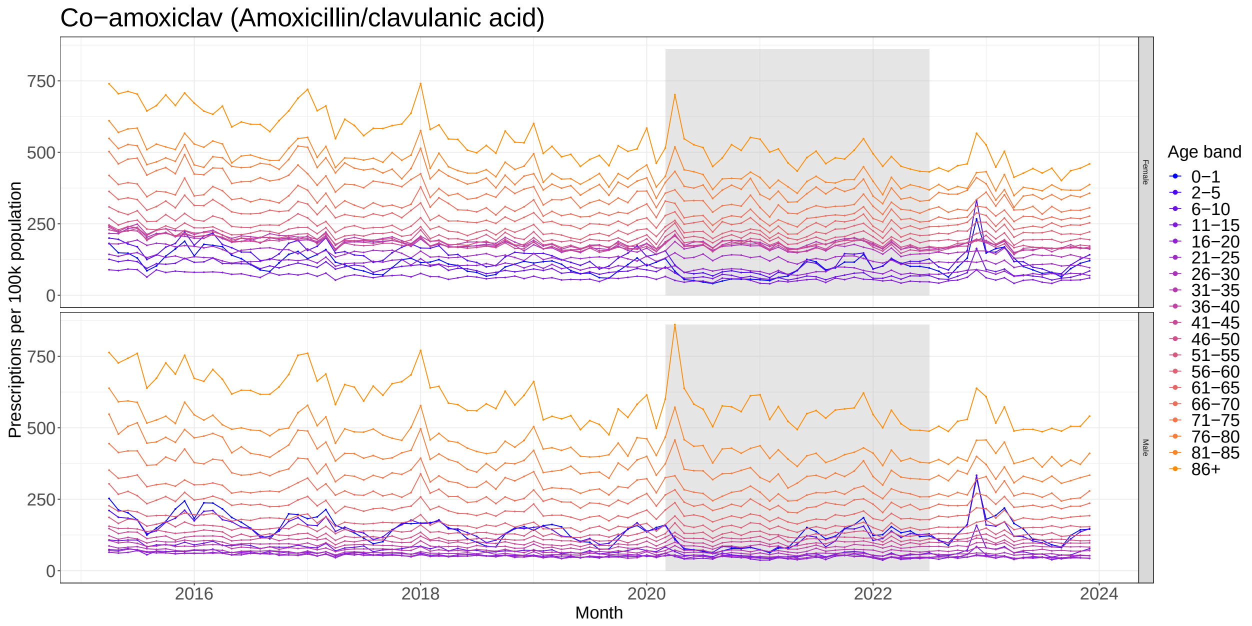


Figure S8: Prescription rate per 100'000 population for Co-amoxiclav (Amoxicillin/clavulanic acid) . Colours indicate age groups, facets indicate sex. Grey shading indicates years of Covid-19 interventions

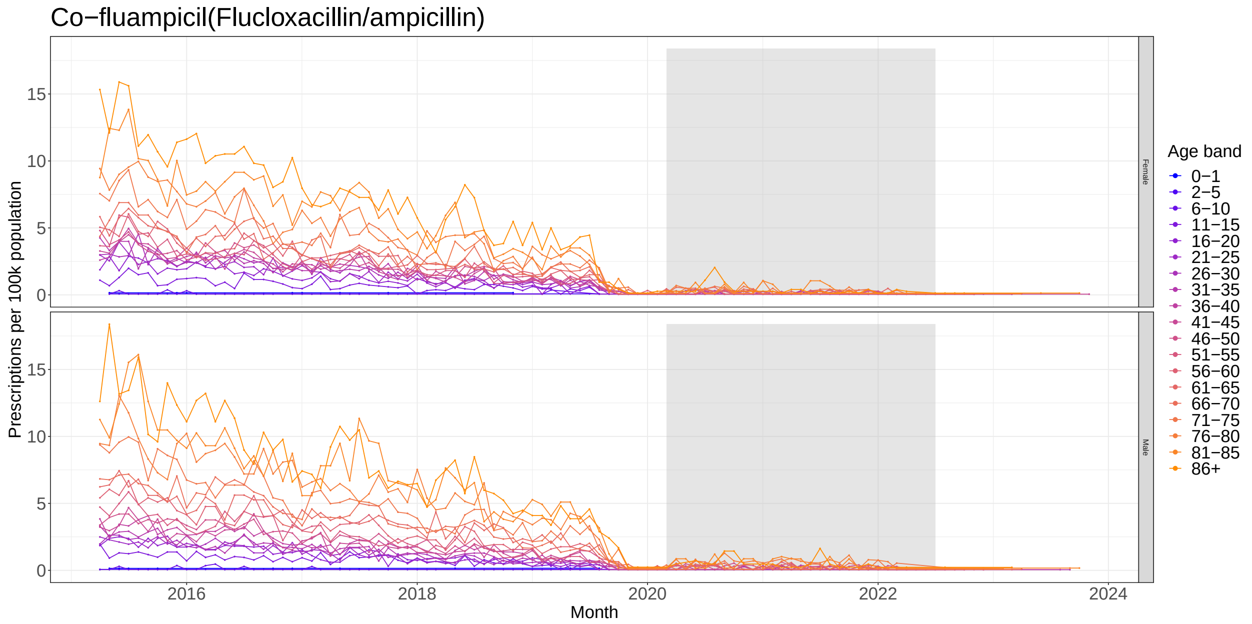


Figure S9: Prescription rate per 100'000 population for Co-fluampicil(Flucloxacillin/ampicillin) . Colours indicate age groups, facets indicate sex. Grey shading indicates years of Covid-19 interventions

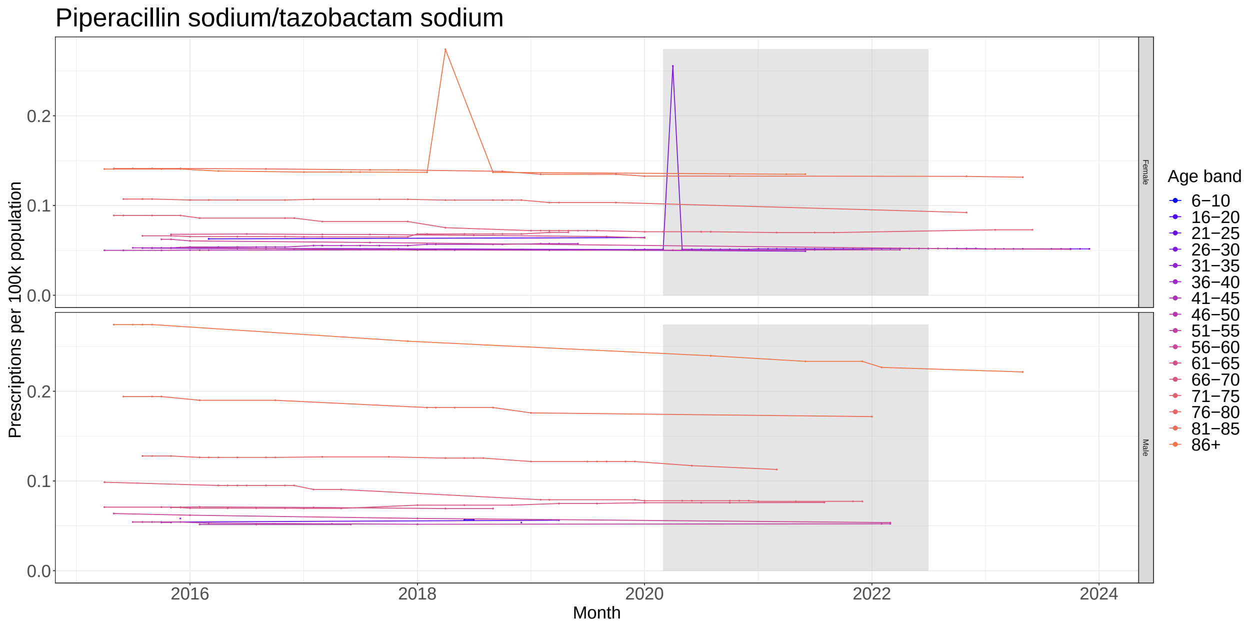


Figure S10: Prescription rate per 100'000 population for Piperacillin sodium/tazobactam sodium . Colours indicate age groups, facets indicate sex. Grey shading indicates years of Covid-19 interventions

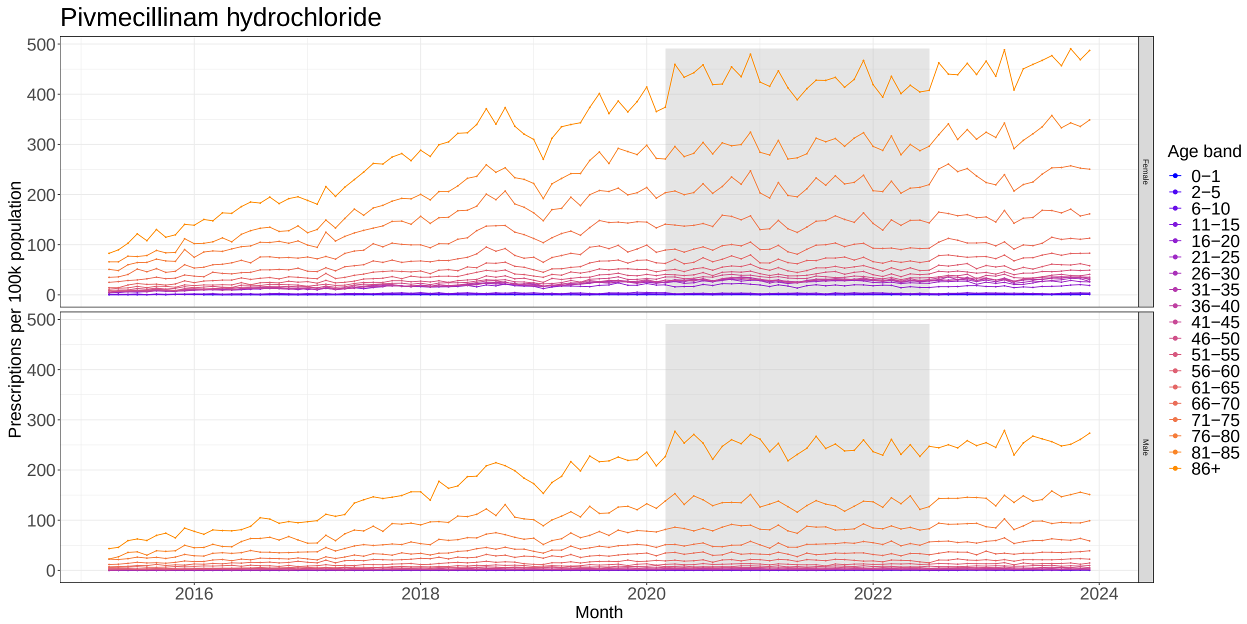


Figure S11: Prescription rate per 100'000 population for Pivmecillinam hydrochloride . Colours indicate age groups, facets indicate sex. Grey shading indicates years of Covid-19 interventions

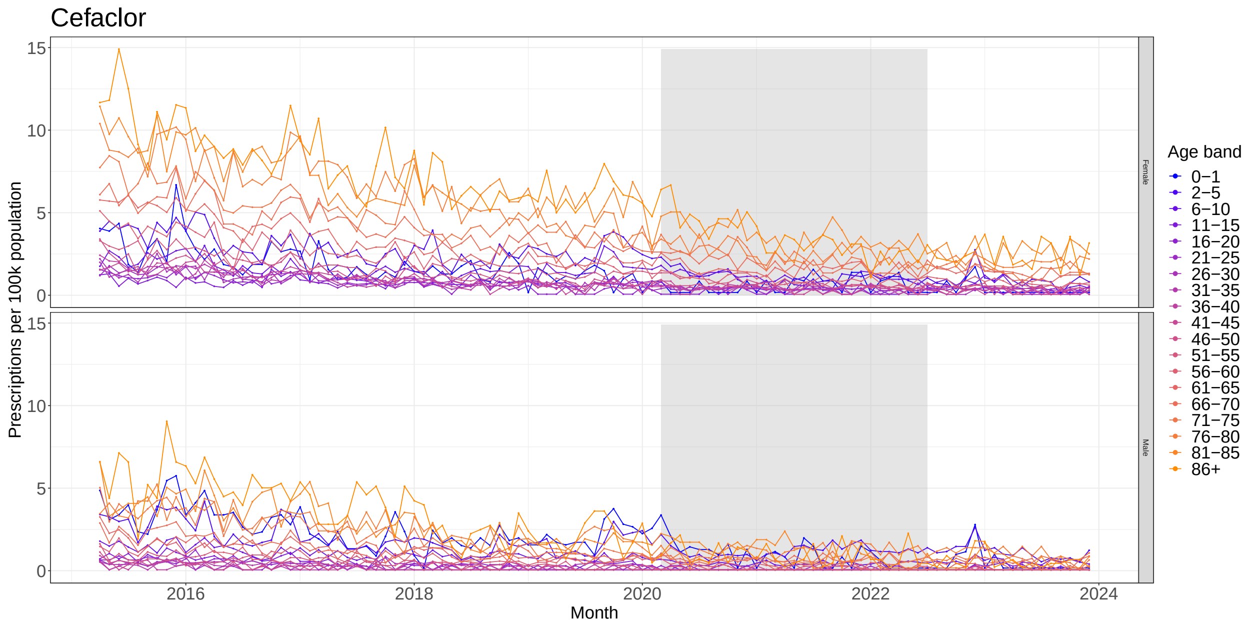


Figure S12: Prescription rate per 100'000 population for Cefaclor . Colours indicate age groups, facets indicate sex. Grey shading indicates years of Covid-19 interventions

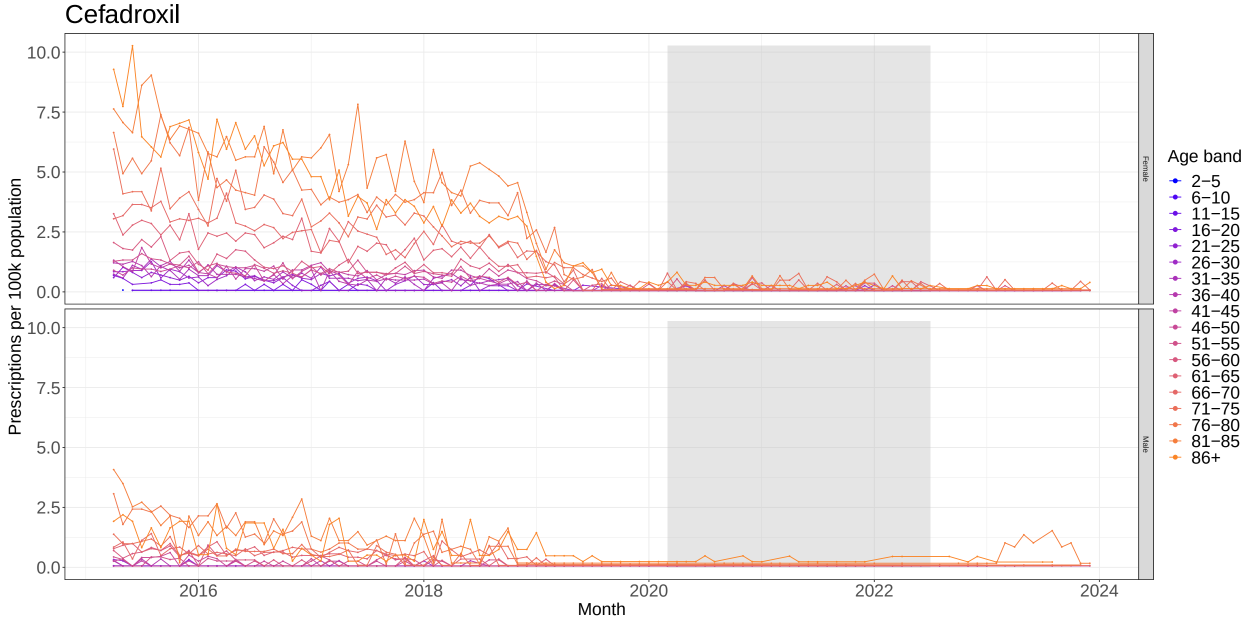


Figure S13: Prescription rate per 100'000 population for Cefadroxil . Colours indicate age groups, facets indicate sex. Grey shading indicates years of Covid-19 interventions

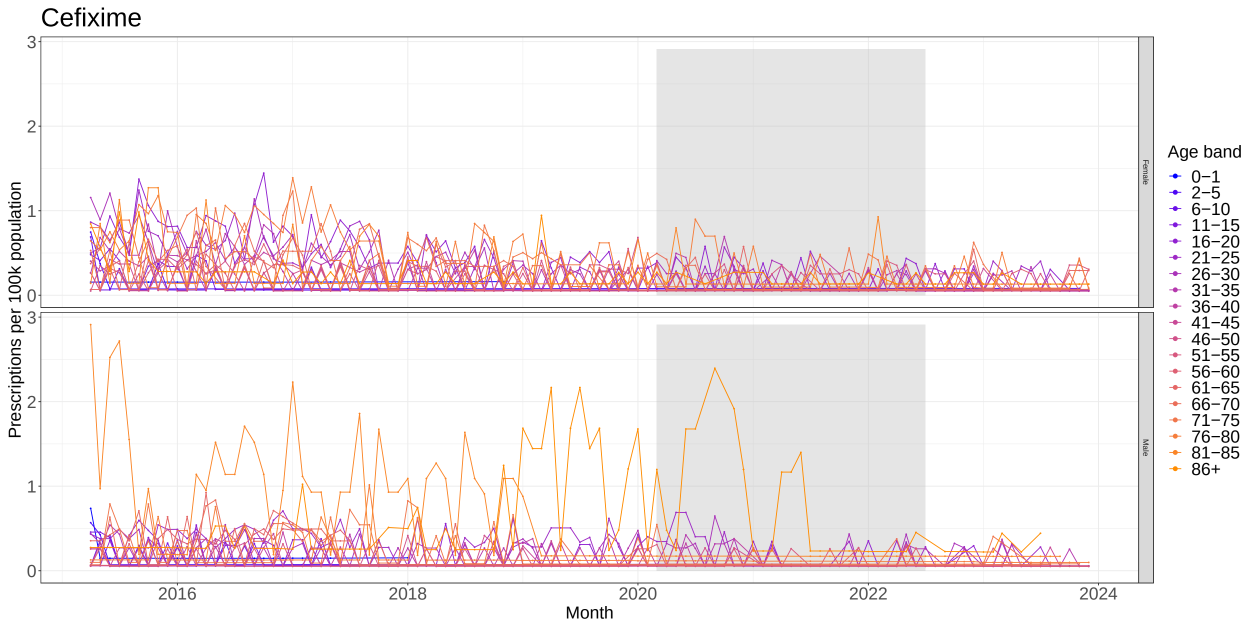


Figure S14: Prescription rate per 100'000 population for Cefixime . Colours indicate age groups, facets indicate sex. Grey shading indicates years of Covid-19 interventions

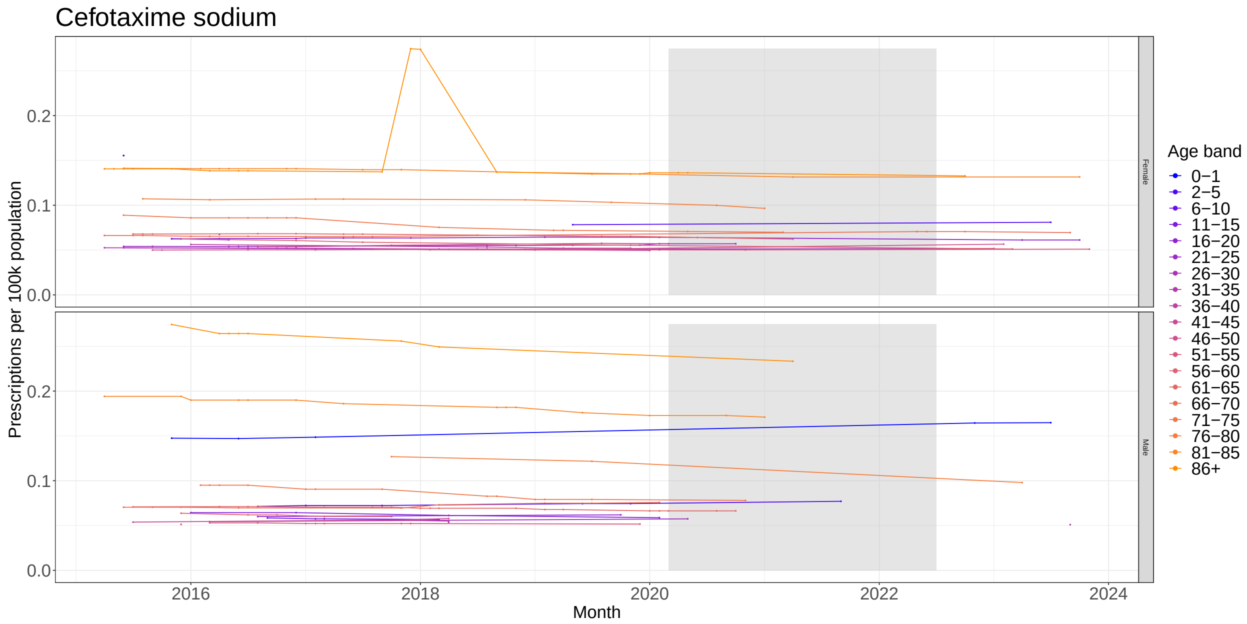


Figure S15: Prescription rate per 100'000 population for Cefotaxime sodium . Colours indicate age groups, facets indicate sex. Grey shading indicates years of Covid-19 interventions

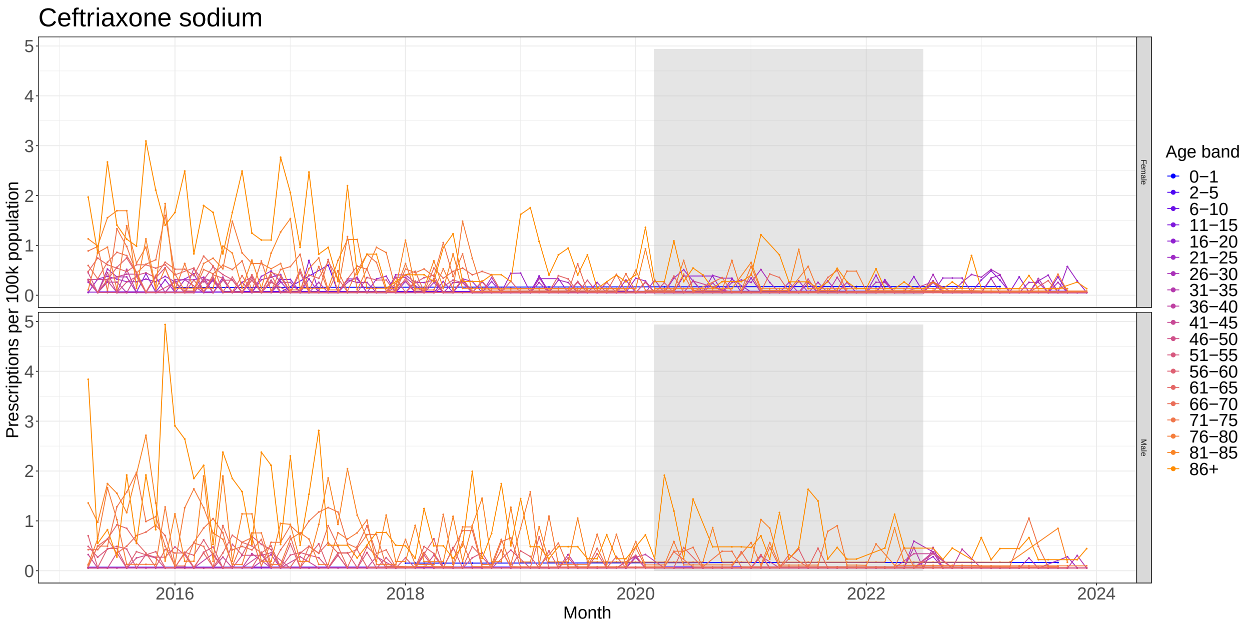


Figure S16: Prescription rate per 100'000 population for Ceftriaxone sodium . Colours indicate age groups, facets indicate sex. Grey shading indicates years of Covid-19 interventions

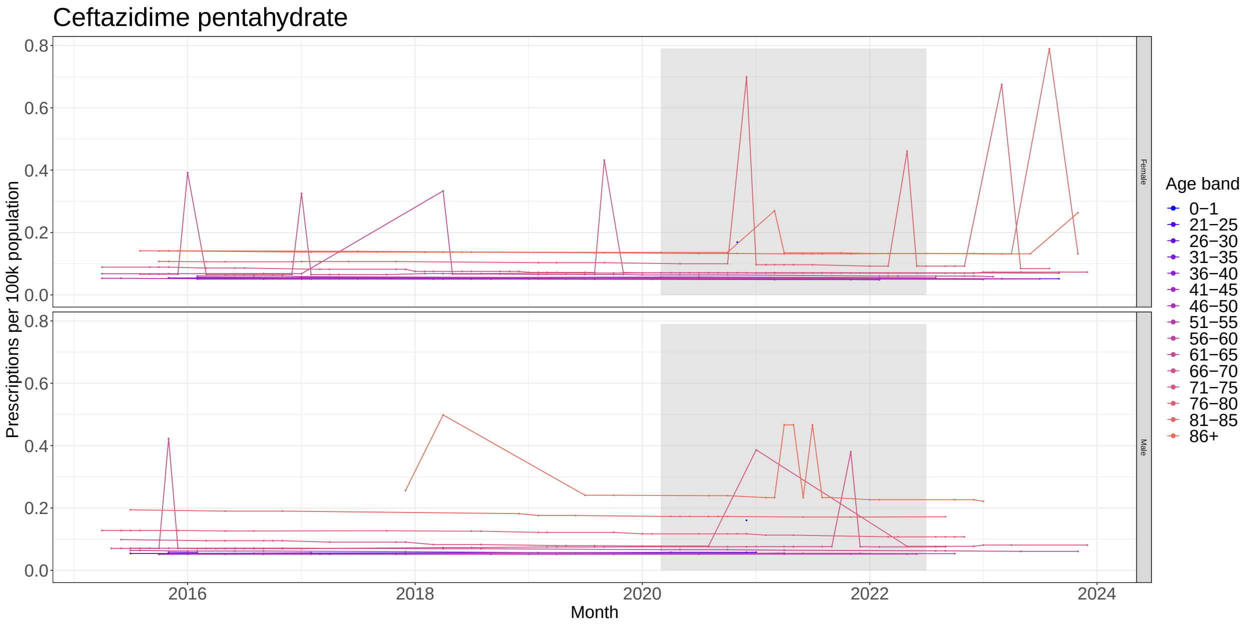


Figure S17: Prescription rate per 100'000 population for Ceftazidime pentahydrate . Colours indicate age groups, facets indicate sex. Grey shading indicates years of Covid-19 interventions

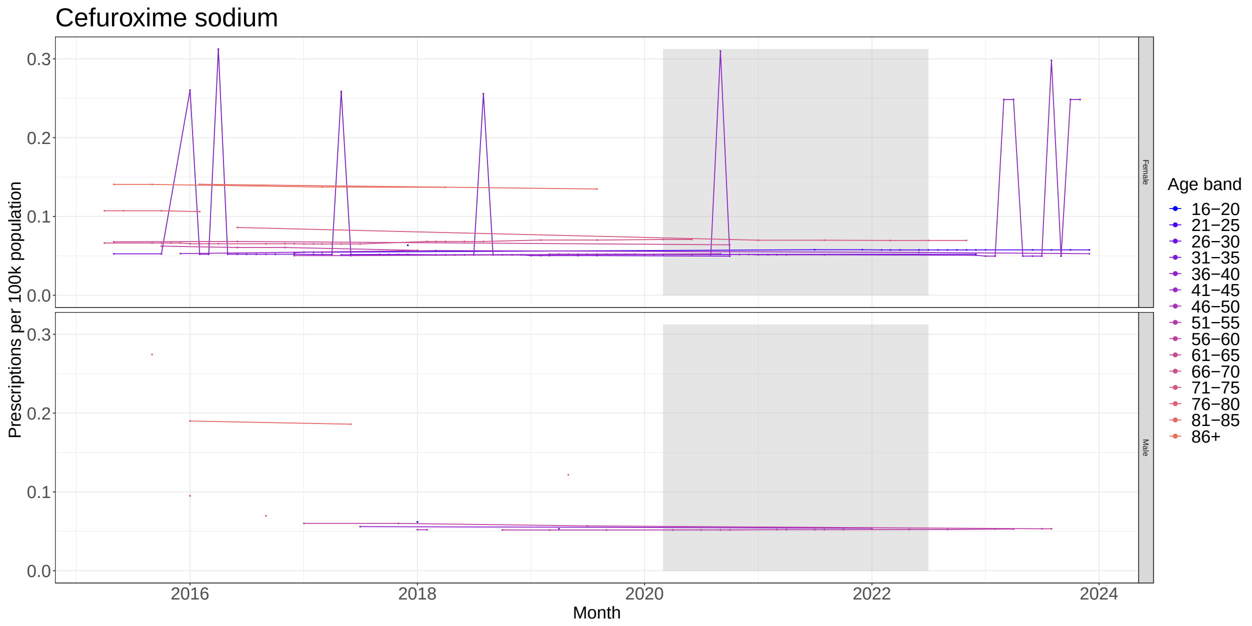


Figure S18: Prescription rate per 100'000 population for Cefuroxime sodium . Colours indicate age groups, facets indicate sex. Grey shading indicates years of Covid-19 interventions

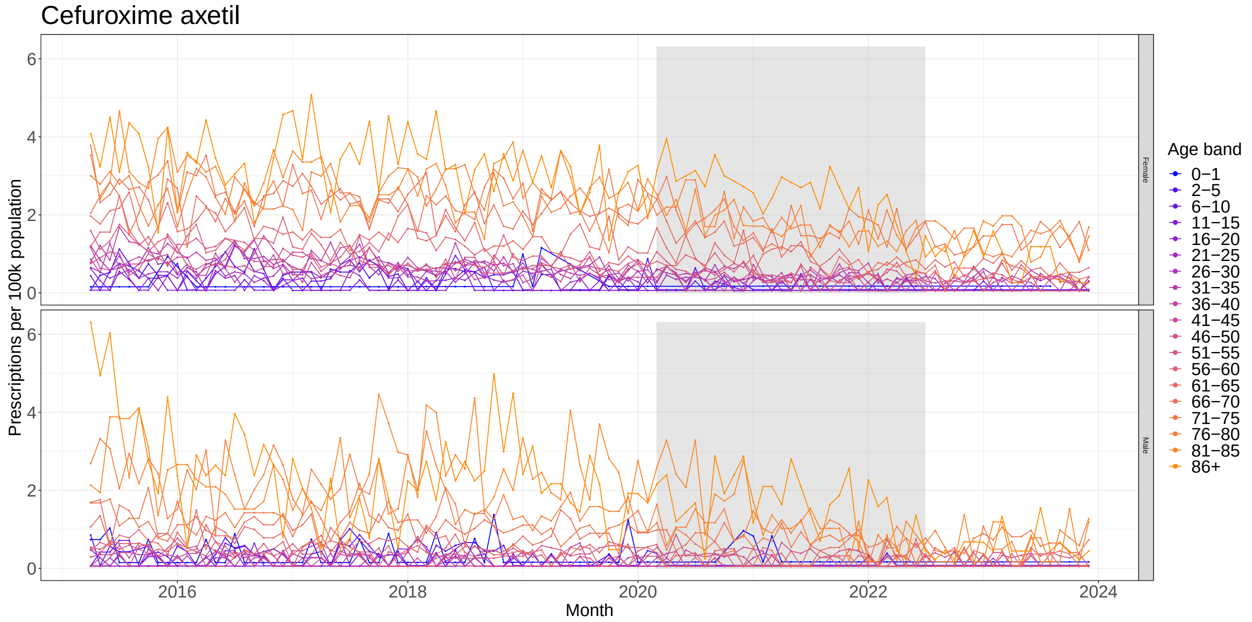


Figure S19: Prescription rate per 100'000 population for Cefuroxime axetil . Colours indicate age groups, facets indicate sex. Grey shading indicates years of Covid-19 interventions

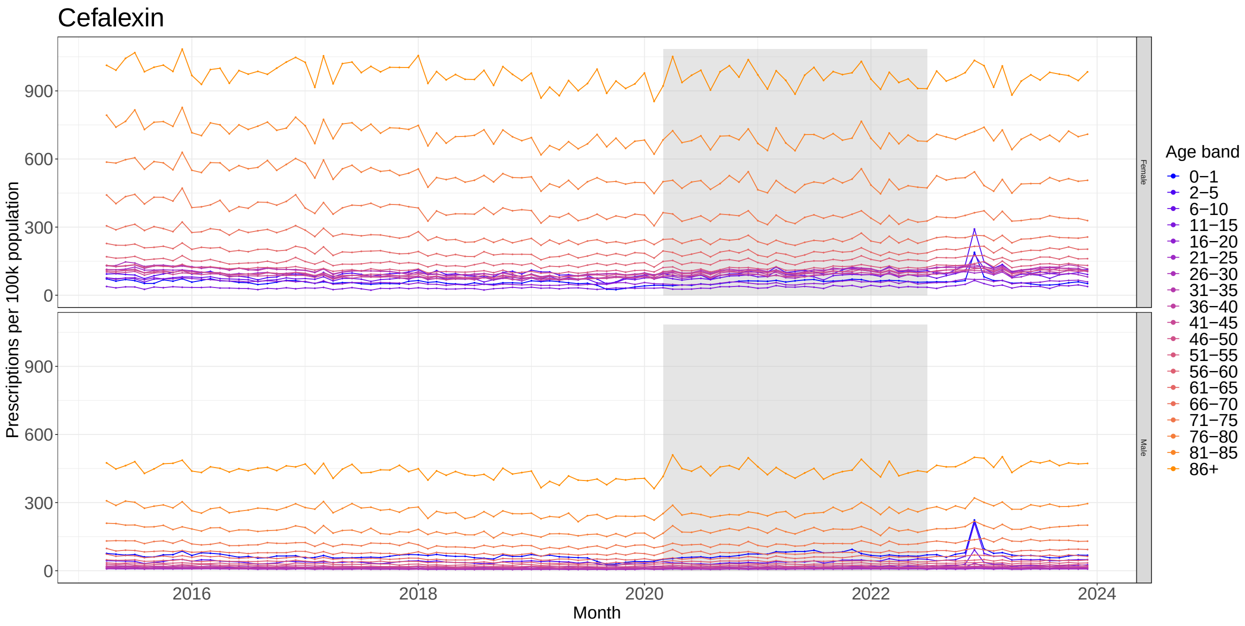


Figure S20: Prescription rate per 100’000 population for Cefalexin . Colours indicate age groups, facets indicate sex. Grey shading indicates years of Covid-19 interventions

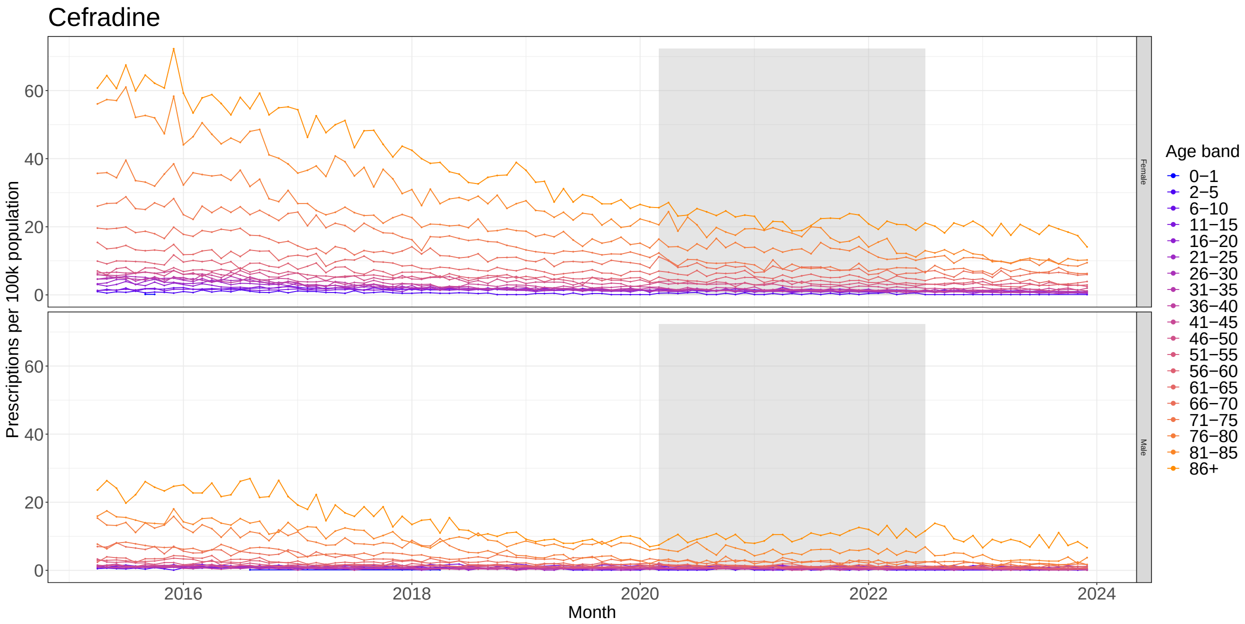


Figure S21: Prescription rate per 100’000 population for Cefradine . Colours indicate age groups, facets indicate sex. Grey shading indicates years of Covid-19 interventions

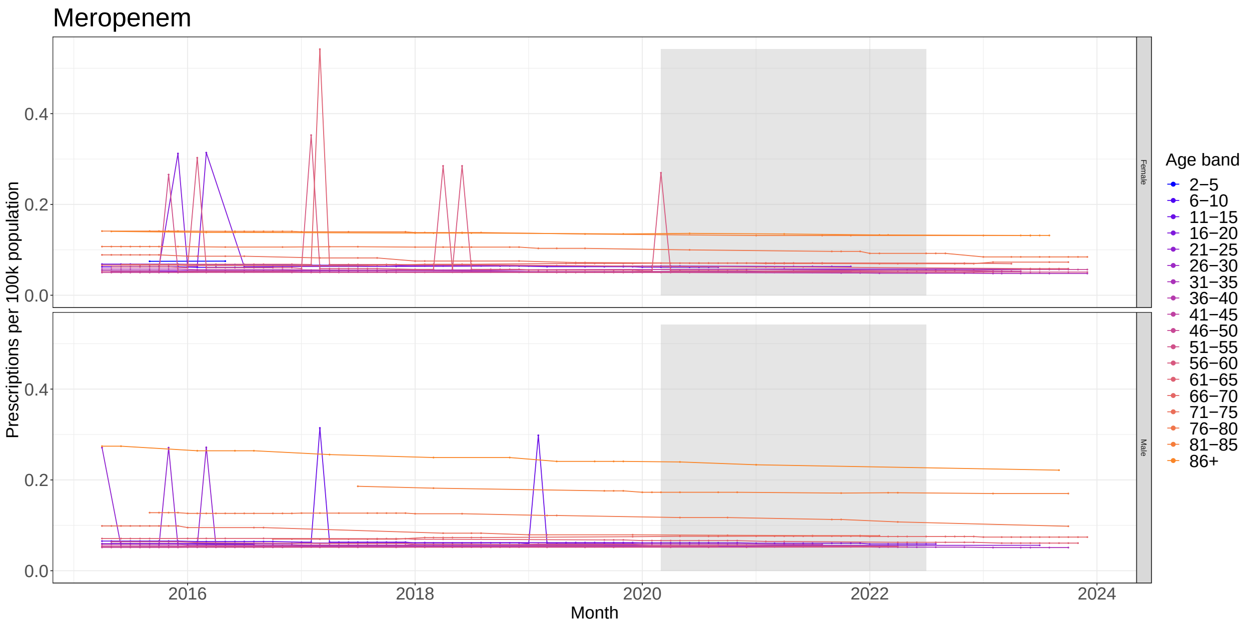


Figure S22: Prescription rate per 100'000 population for Meropenem . Colours indicate age groups, facets indicate sex. Grey shading indicates years of Covid-19 interventions

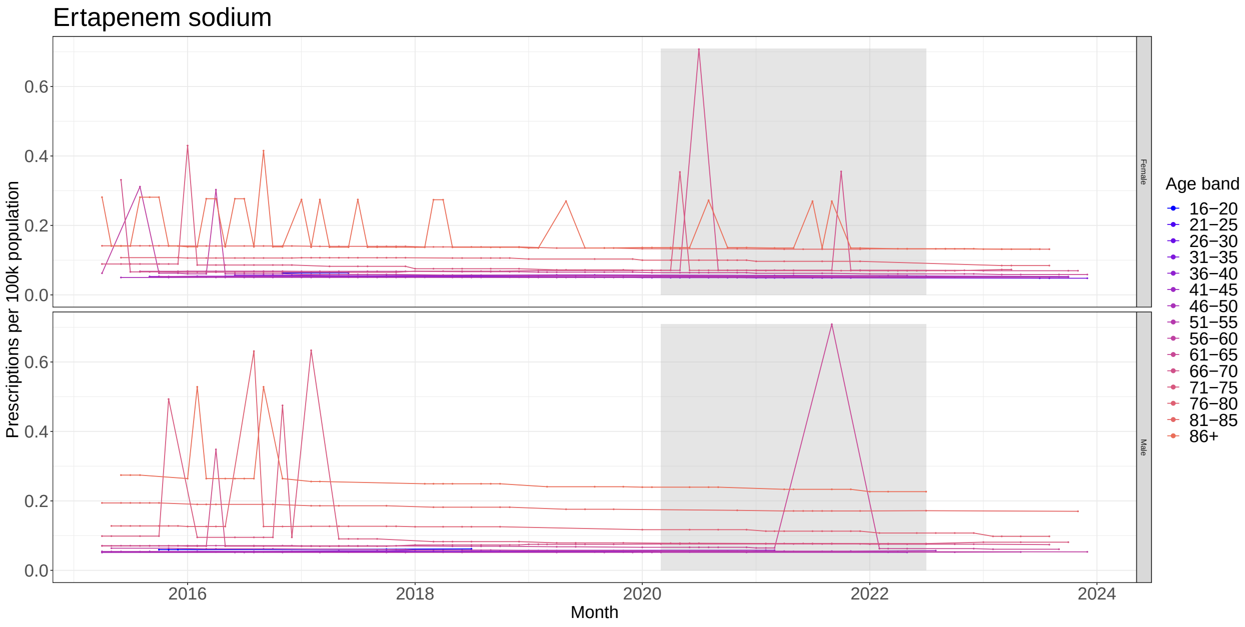


Figure S23: Prescription rate per 100'000 population for Ertapenem sodium . Colours indicate age groups, facets indicate sex. Grey shading indicates years of Covid-19 interventions

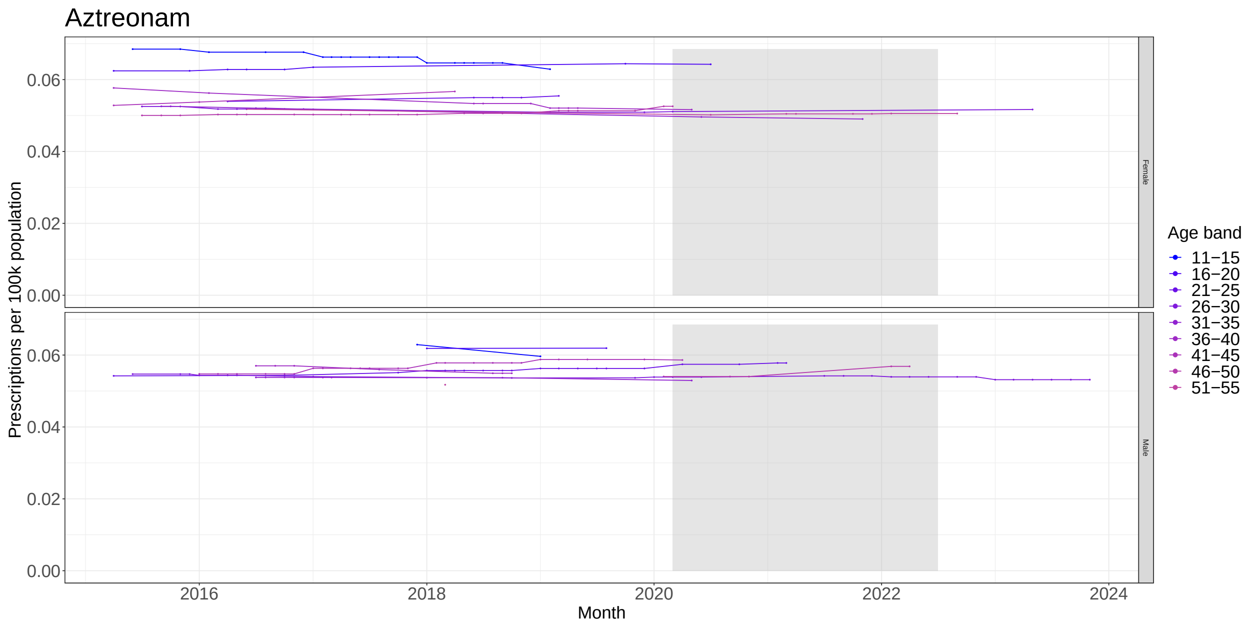


Figure S24: Prescription rate per 100’000 population for Aztreonam . Colours indicate age groups, facets indicate sex. Grey shading indicates years of Covid-19 interventions

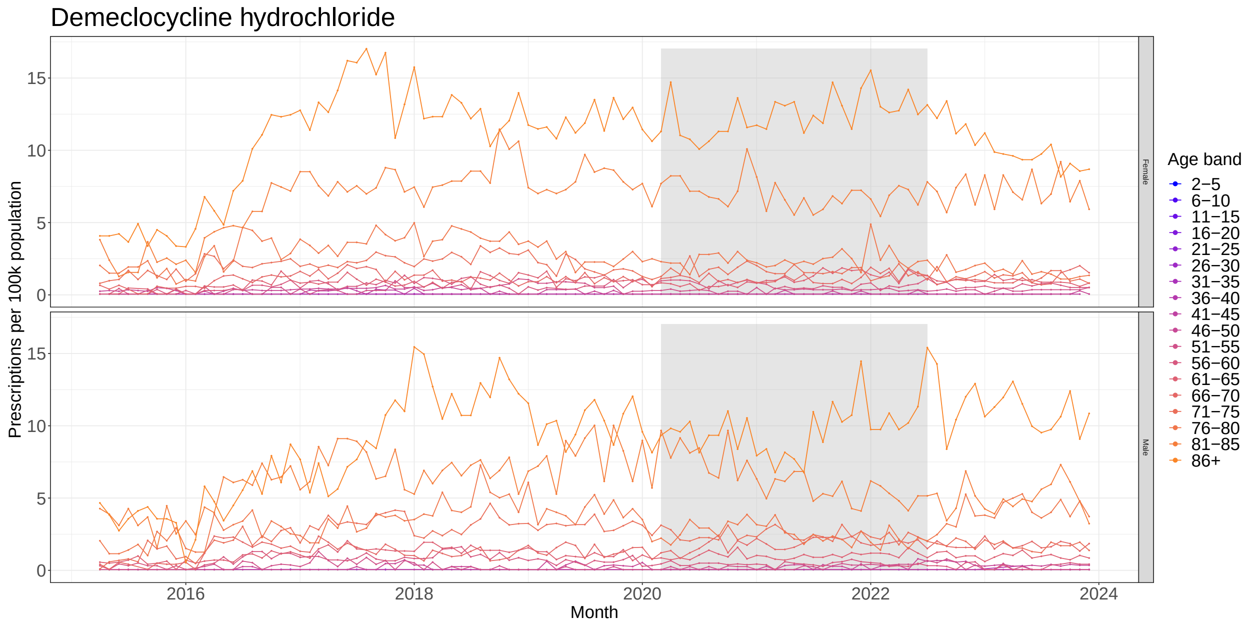


Figure S25: Prescription rate per 100’000 population for Demeclocycline hydrochloride . Colours indicate age groups, facets indicate sex. Grey shading indicates years of Covid-19 interventions

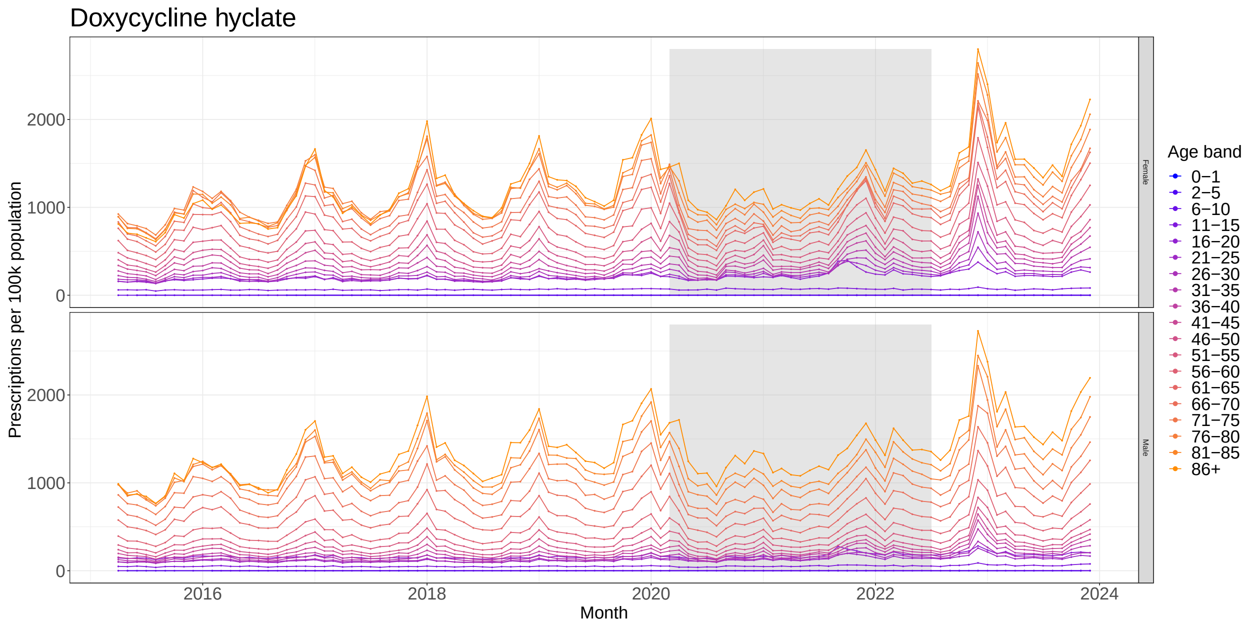


Figure S26: Prescription rate per 100’000 population for Doxycycline hyclate . Colours indicate age groups, facets indicate sex. Grey shading indicates years of Covid-19 interventions

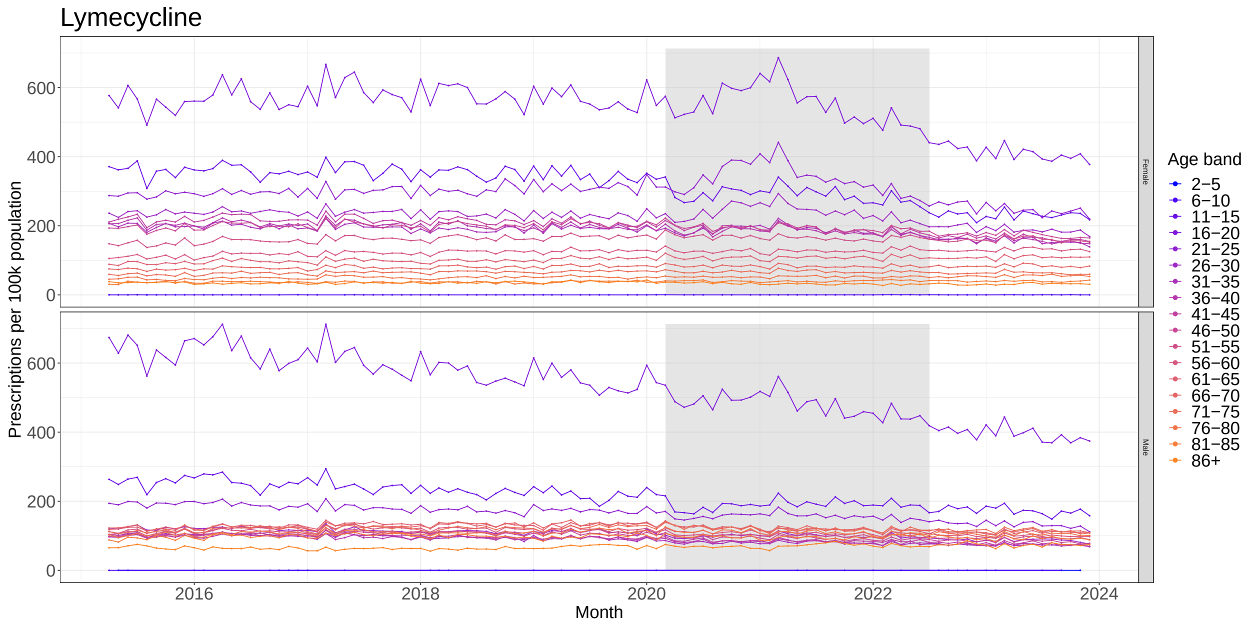


Figure S27: Prescription rate per 100’000 population for Lymecycline . Colours indicate age groups, facets indicate sex. Grey shading indicates years of Covid-19 interventions

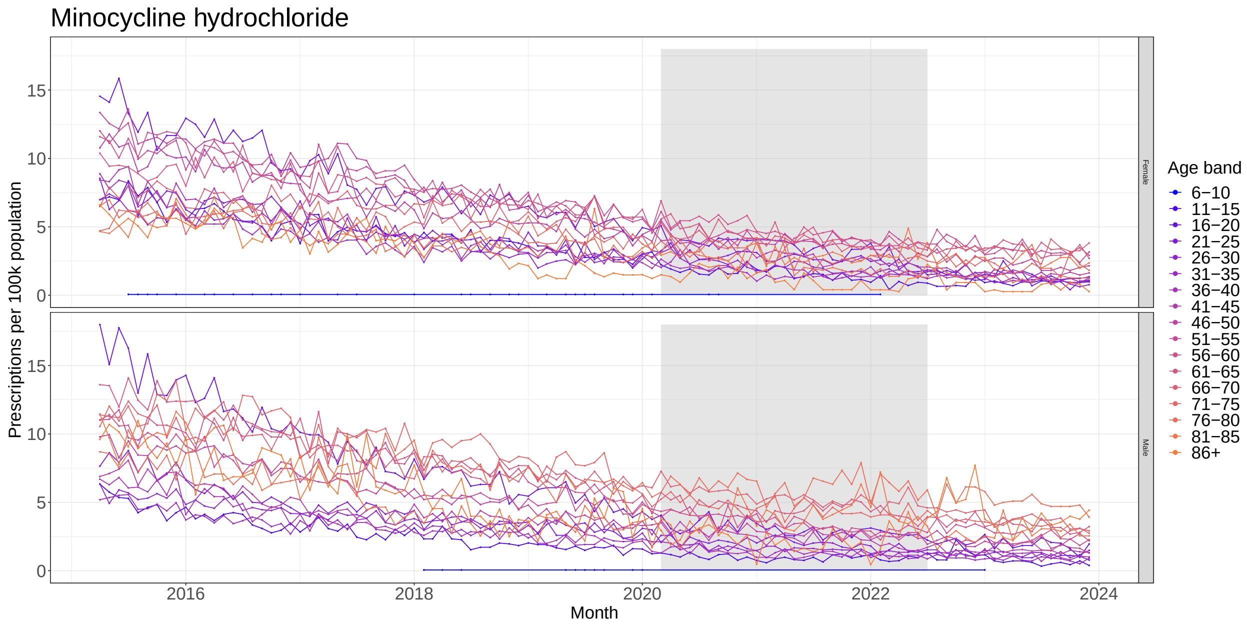


Figure S28: Prescription rate per 100’000 population for Minocycline hydrochloride . Colours indicate age groups, facets indicate sex. Grey shading indicates years of Covid-19 interventions

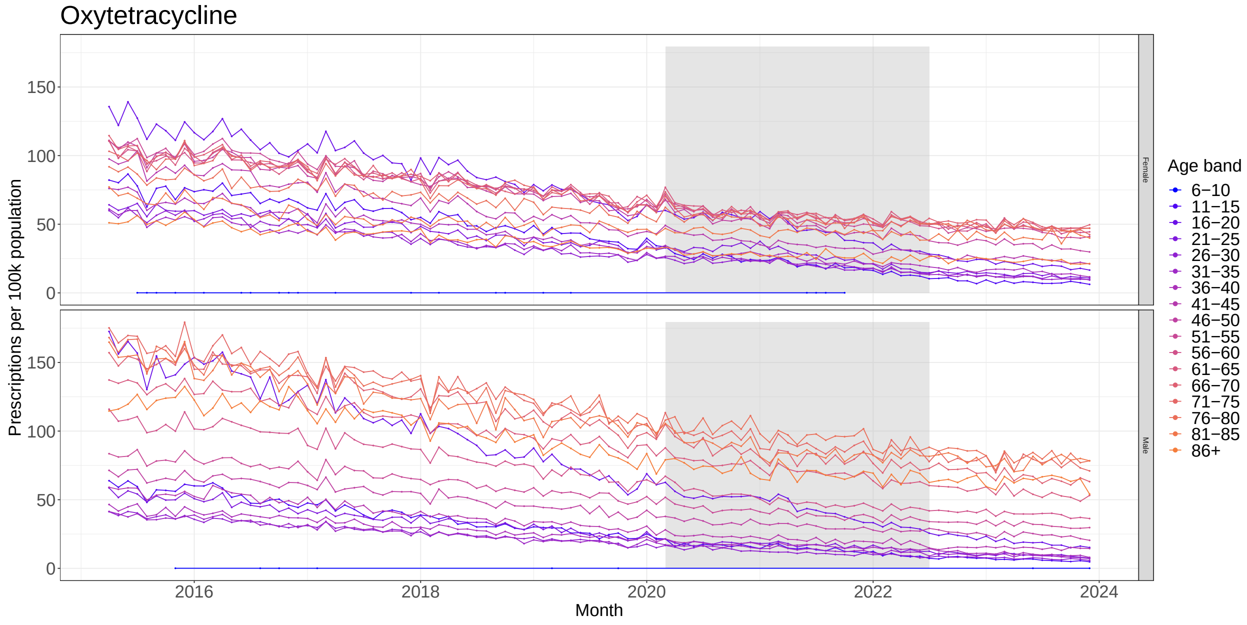


Figure S29: Prescription rate per 100’000 population for Oxytetracycline . Colours indicate age groups, facets indicate sex. Grey shading indicates years of Covid-19 interventions

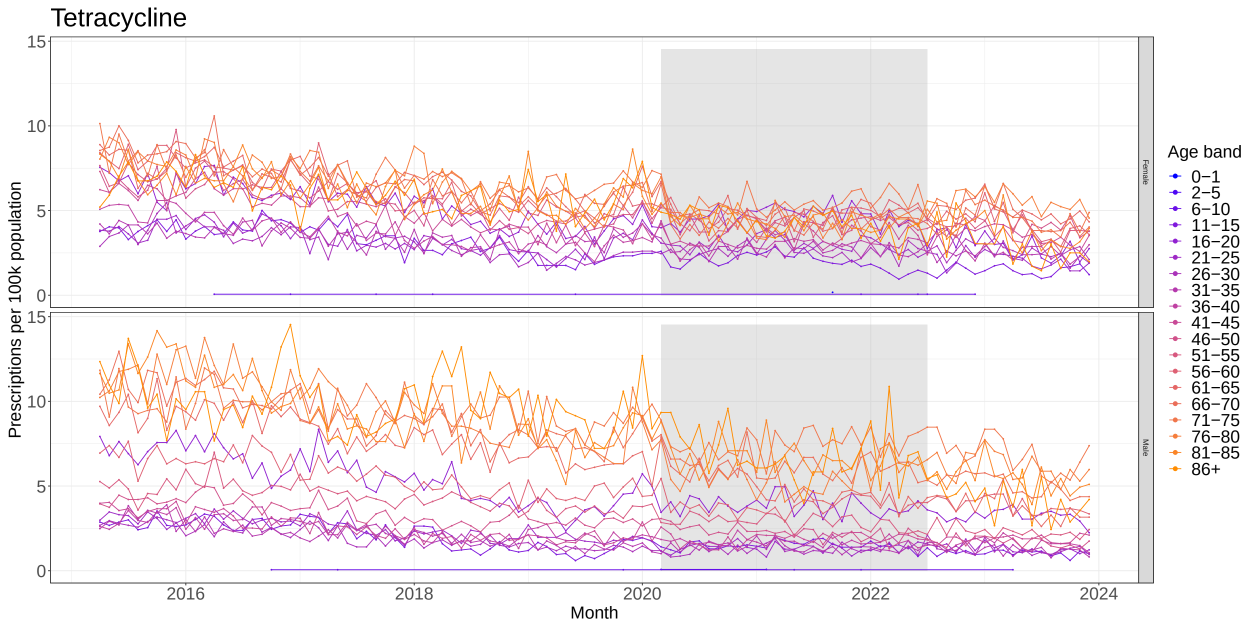


Figure S30: Prescription rate per 100'000 population for Tetracycline . Colours indicate age groups, facets indicate sex. Grey shading indicates years of Covid-19 interventions

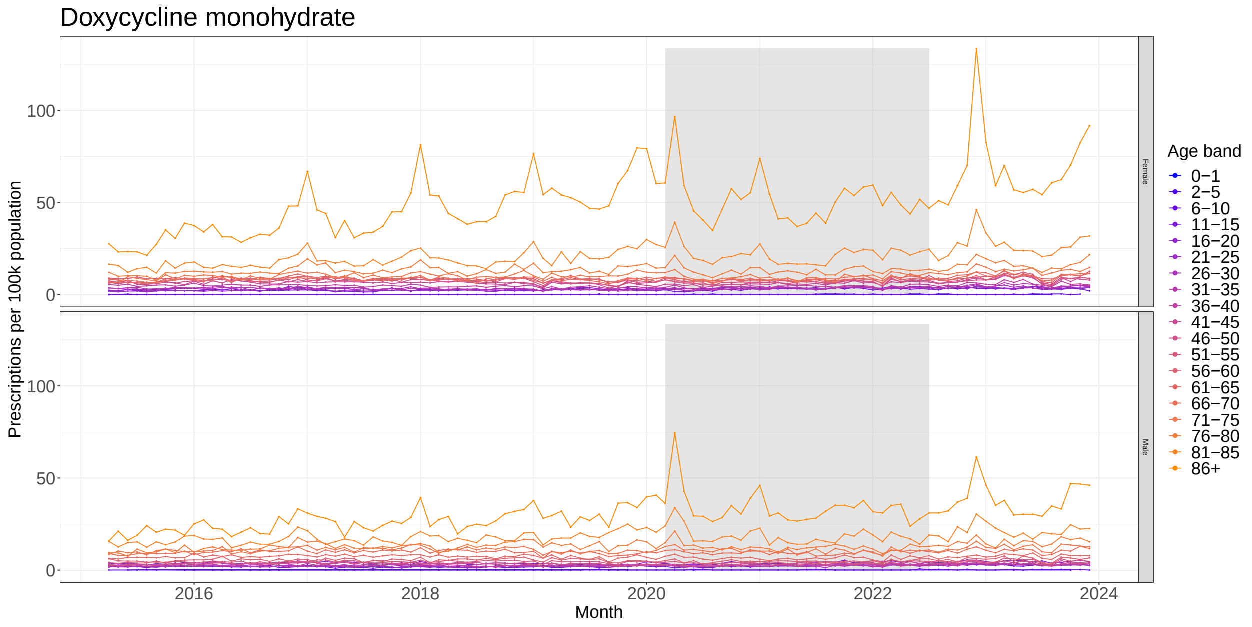


Figure S31: Prescription rate per 100'000 population for Doxycycline monohydrate . Colours indicate age groups, facets indicate sex. Grey shading indicates years of Covid-19 interventions

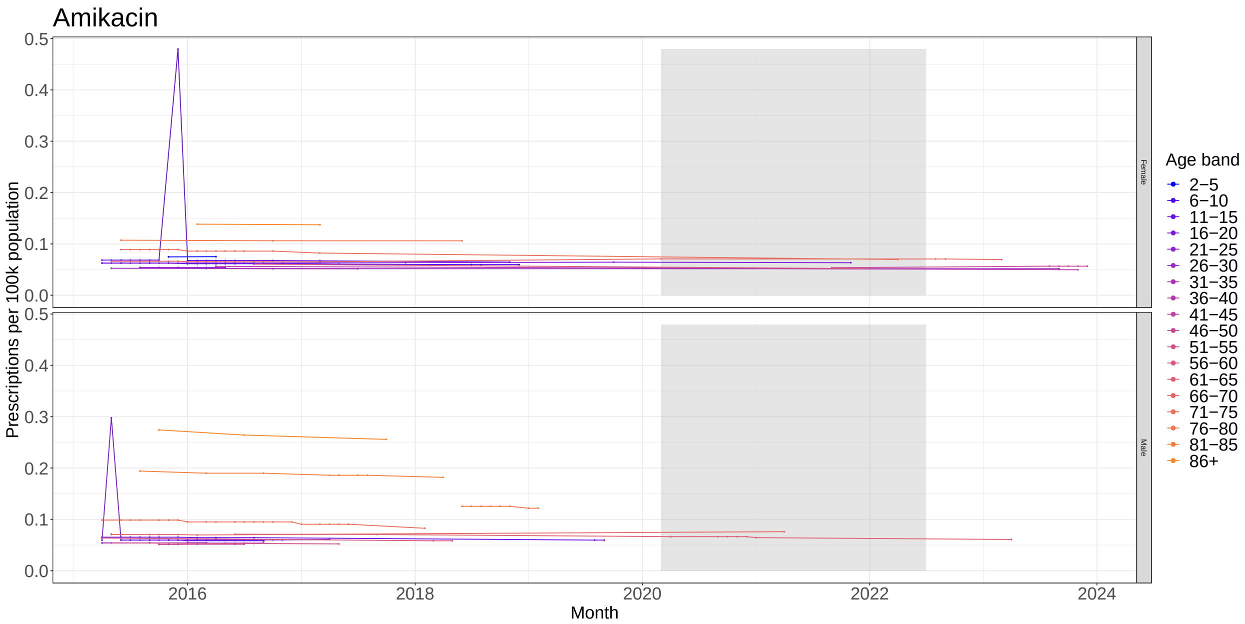


Figure S32: Prescription rate per 100'000 population for Amikacin . Colours indicate age groups, facets indicate sex. Grey shading indicates years of Covid-19 interventions

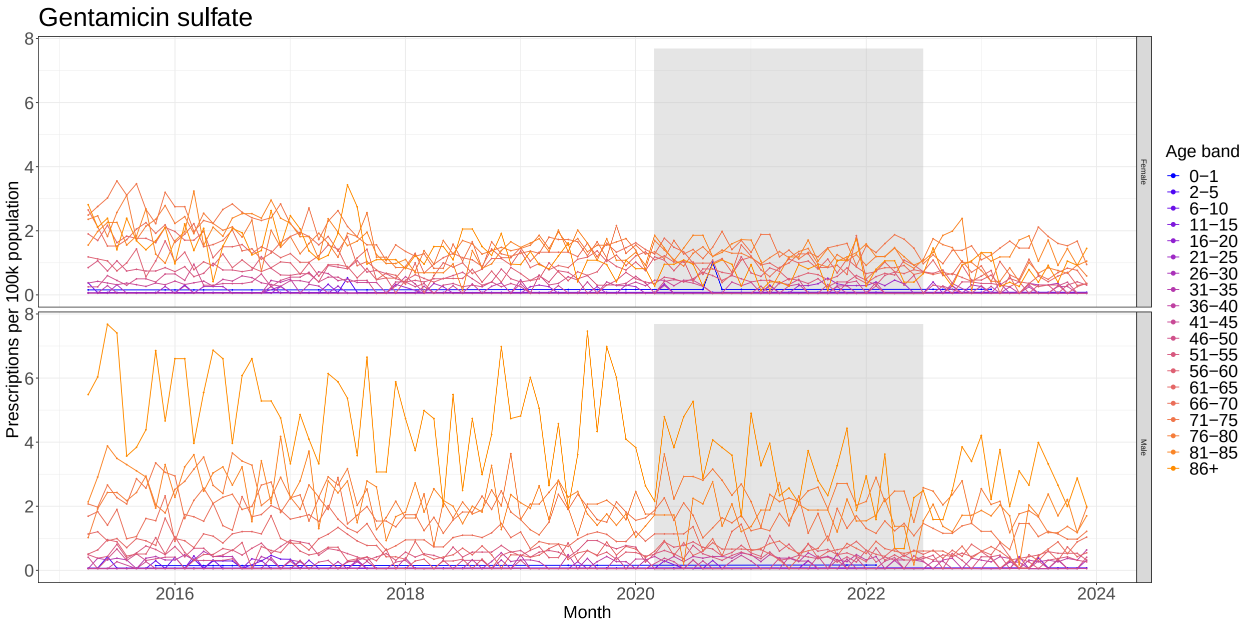


Figure S33: Prescription rate per 100'000 population for Gentamicin sulfate . Colours indicate age groups, facets indicate sex. Grey shading indicates years of Covid-19 interventions

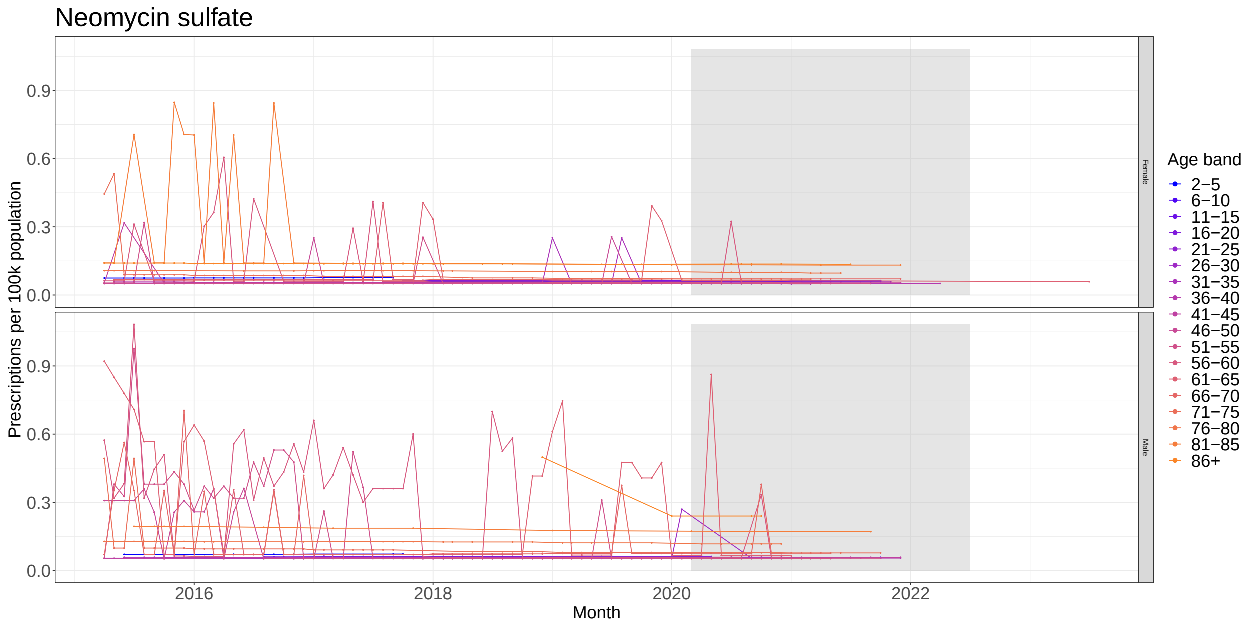


Figure S34: Prescription rate per 100’000 population for Neomycin sulfate . Colours indicate age groups, facets indicate sex. Grey shading indicates years of Covid-19 interventions

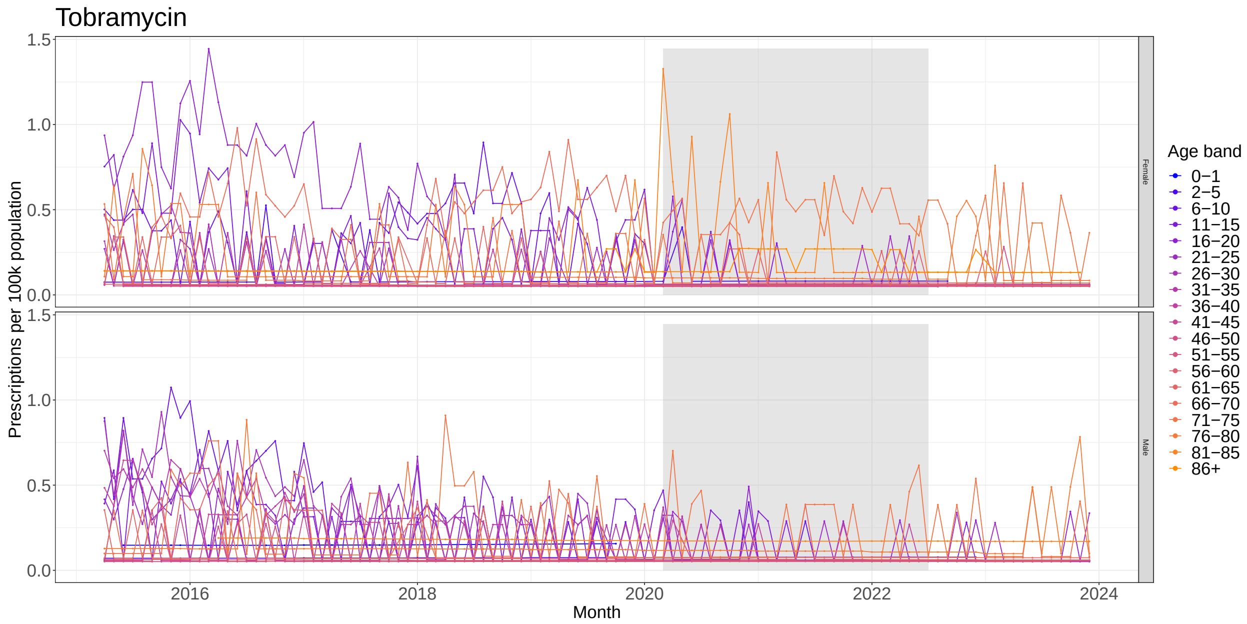


Figure S35: Prescription rate per 100’000 population for Tobramycin . Colours indicate age groups, facets indicate sex. Grey shading indicates years of Covid-19 interventions

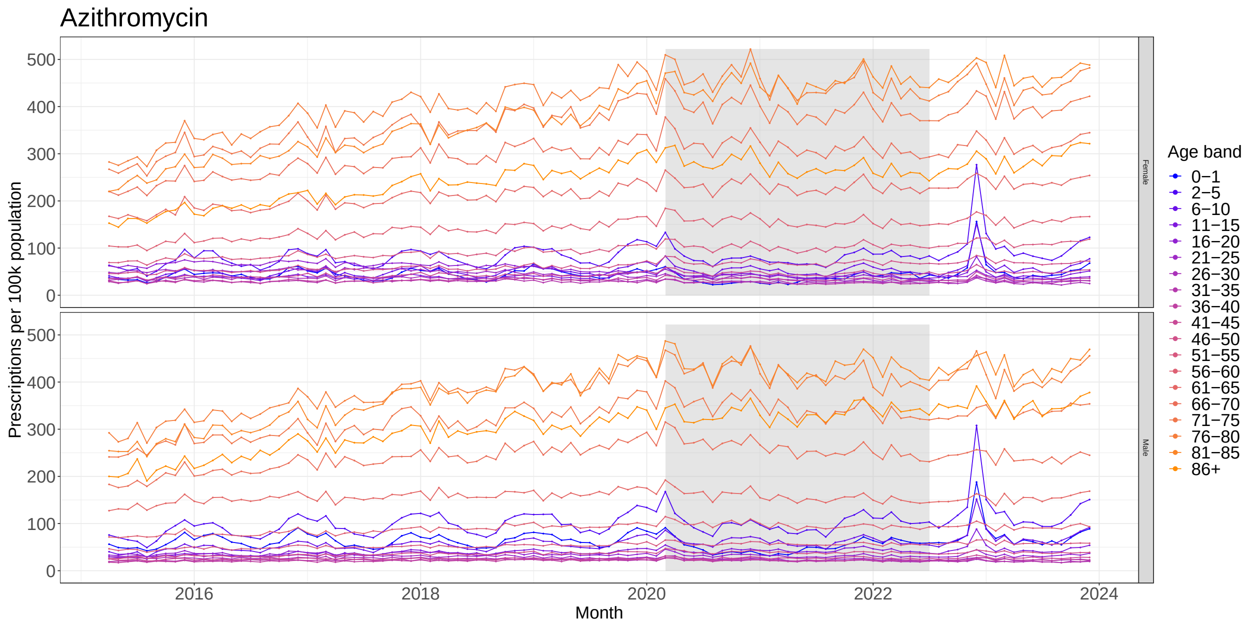


Figure S36: Prescription rate per 100'000 population for Azithromycin . Colours indicate age groups, facets indicate sex. Grey shading indicates years of Covid-19 interventions

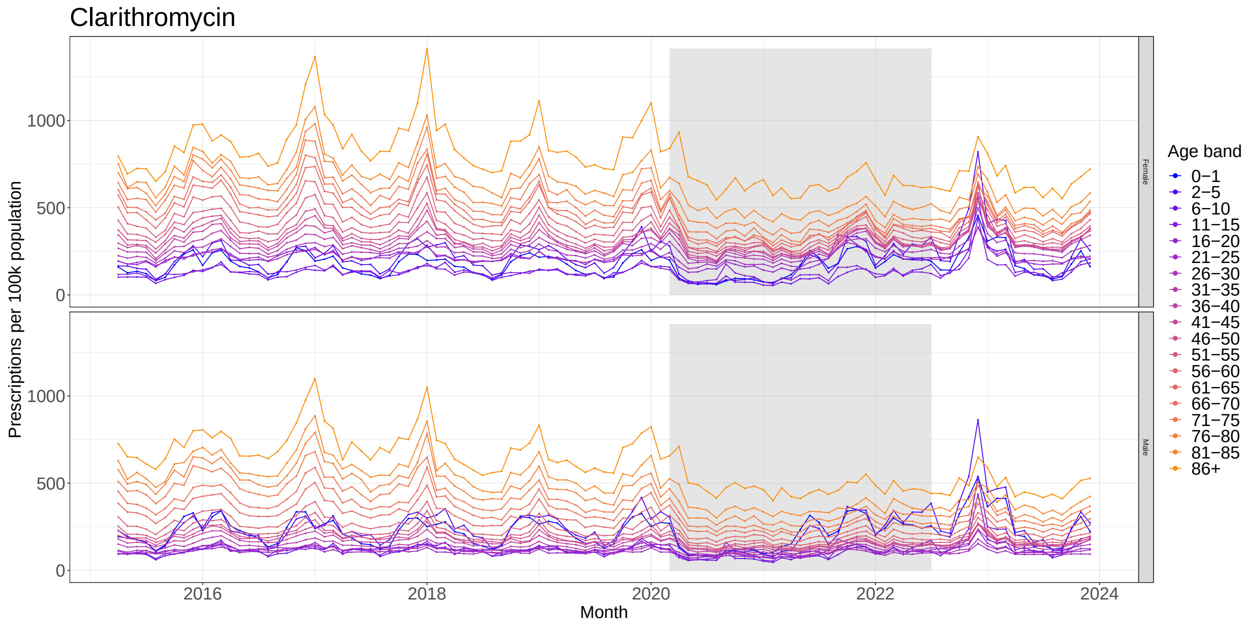


Figure S37: Prescription rate per 100'000 population for Clarithromycin . Colours indicate age groups, facets indicate sex. Grey shading indicates years of Covid-19 interventions

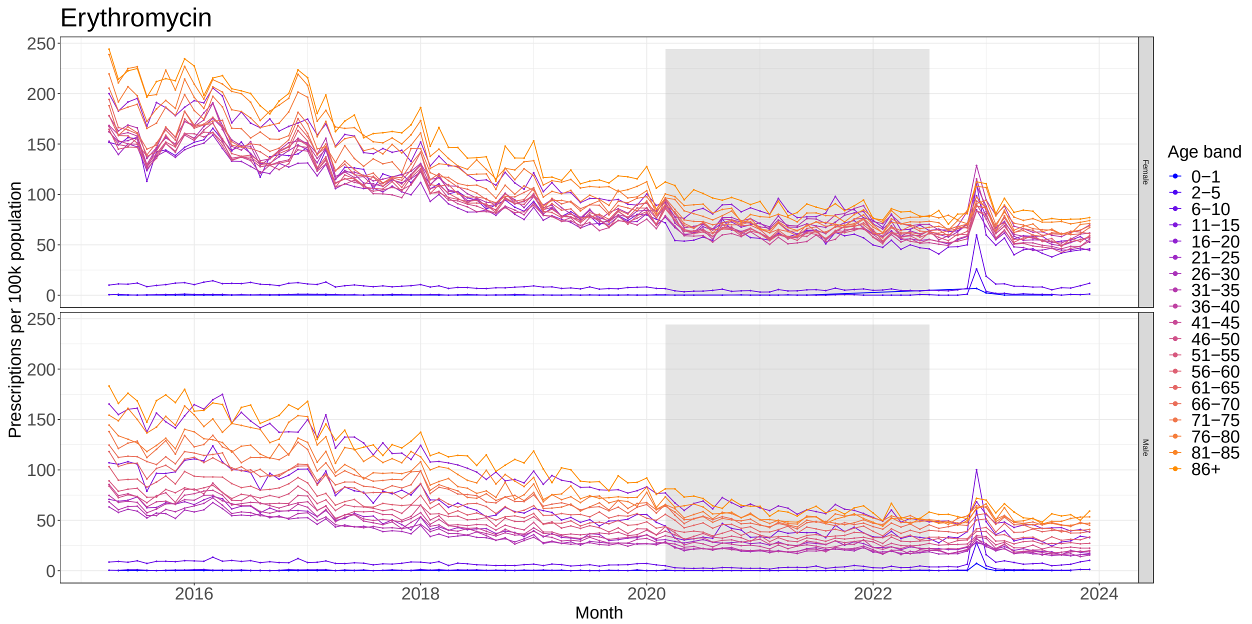


Figure S38: Prescription rate per 100’000 population for Erythromycin . Colours indicate age groups, facets indicate sex. Grey shading indicates years of Covid-19 interventions

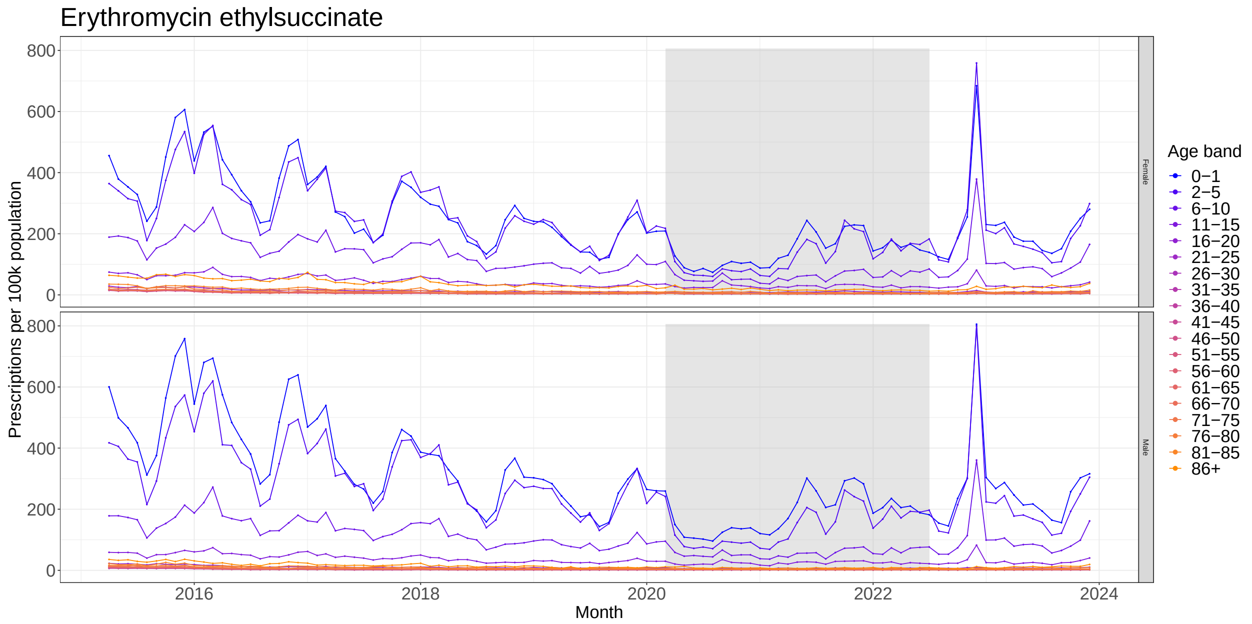


Figure S39: Prescription rate per 100’000 population for Erythromycin ethylsuccinate . Colours indicate age groups, facets indicate sex. Grey shading indicates years of Covid-19 interventions

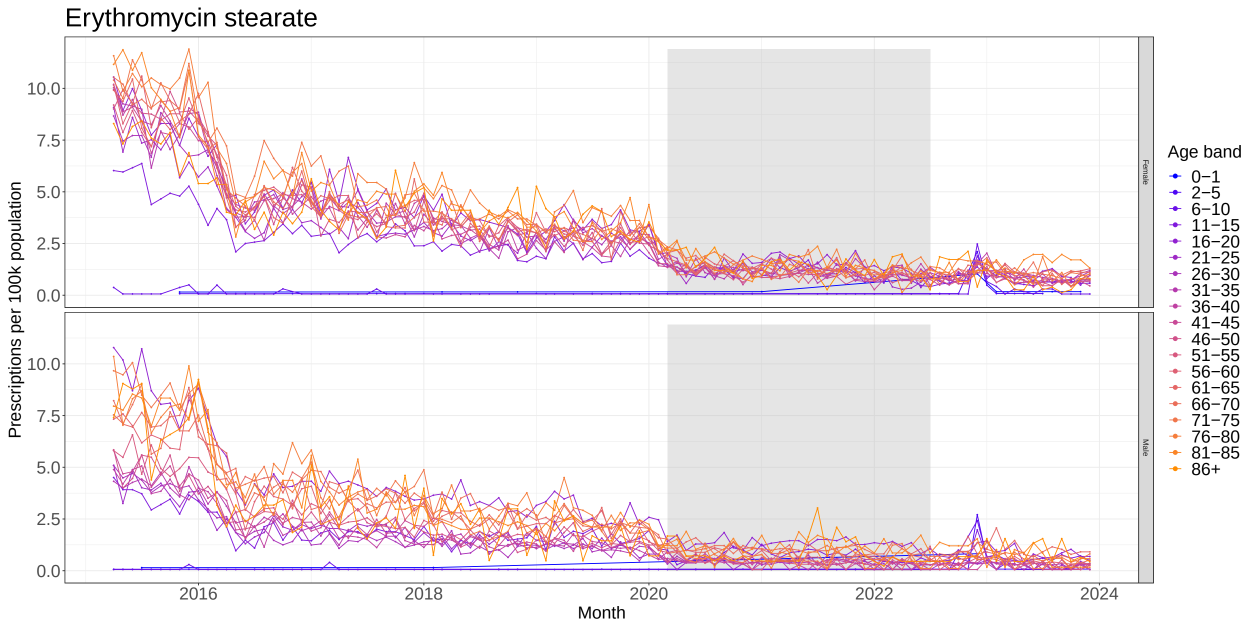


Figure S40: Prescription rate per 100’000 population for Erythromycin stearate . Colours indicate age groups, facets indicate sex. Grey shading indicates years of Covid-19 interventions

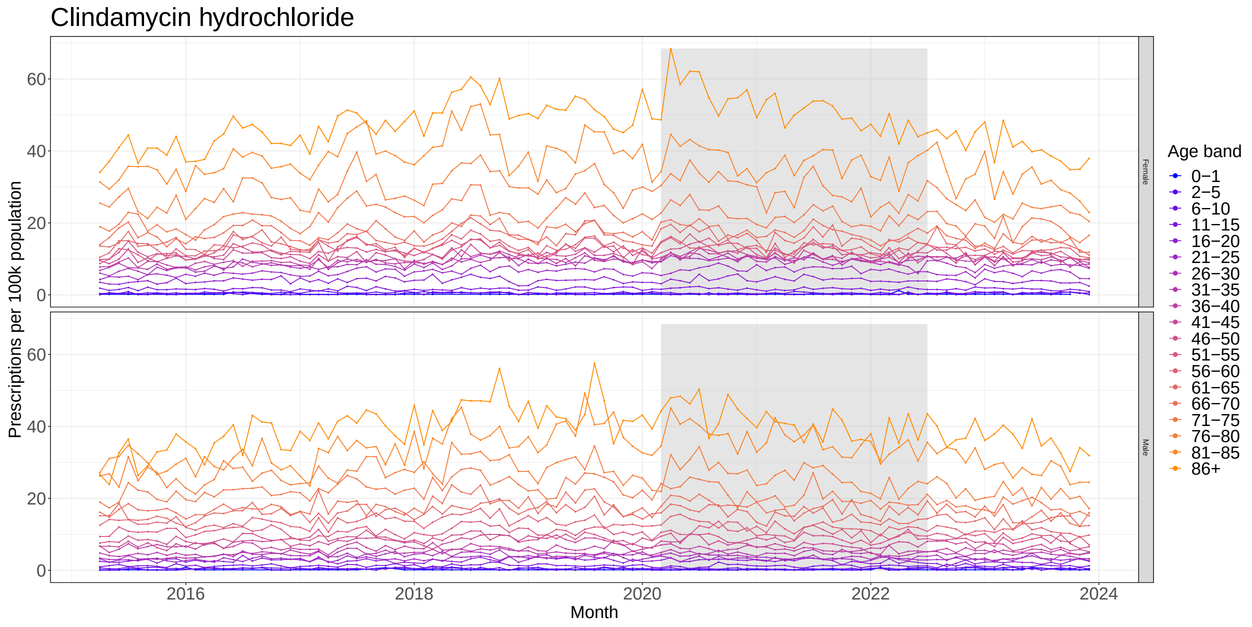


Figure S41: Prescription rate per 100’000 population for Clindamycin hydrochloride . Colours indicate age groups, facets indicate sex. Grey shading indicates years of Covid-19 interventions

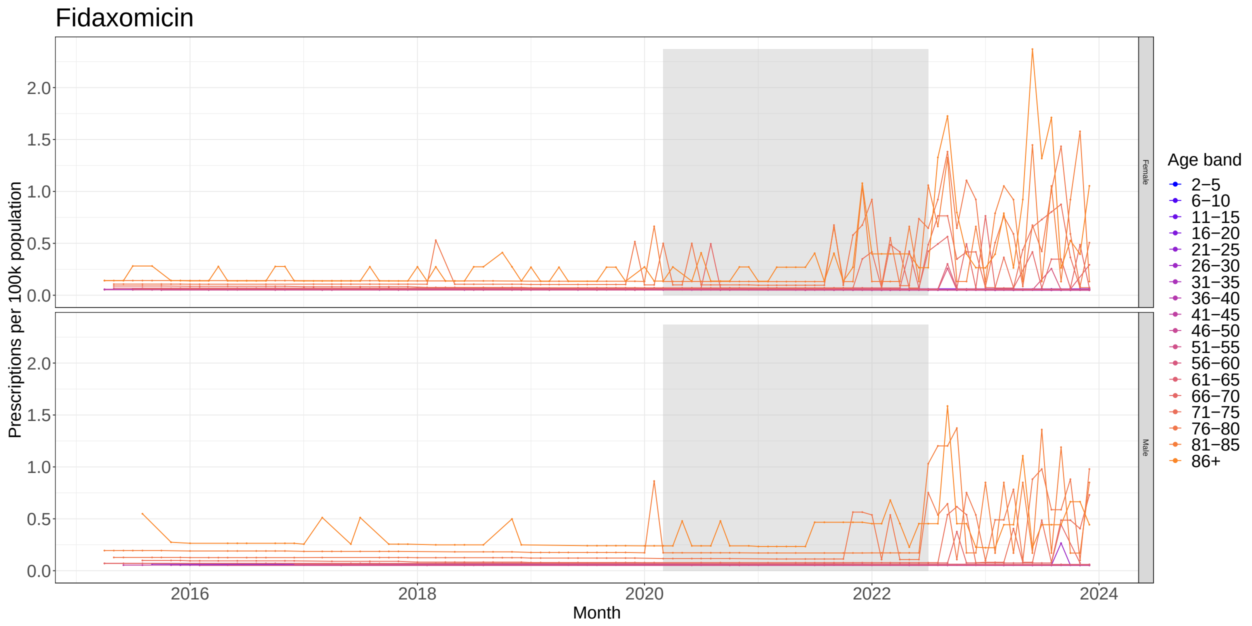


Figure S42: Prescription rate per 100’000 population for Fidaxomicin . Colours indicate age groups, facets indicate sex. Grey shading indicates years of Covid-19 interventions

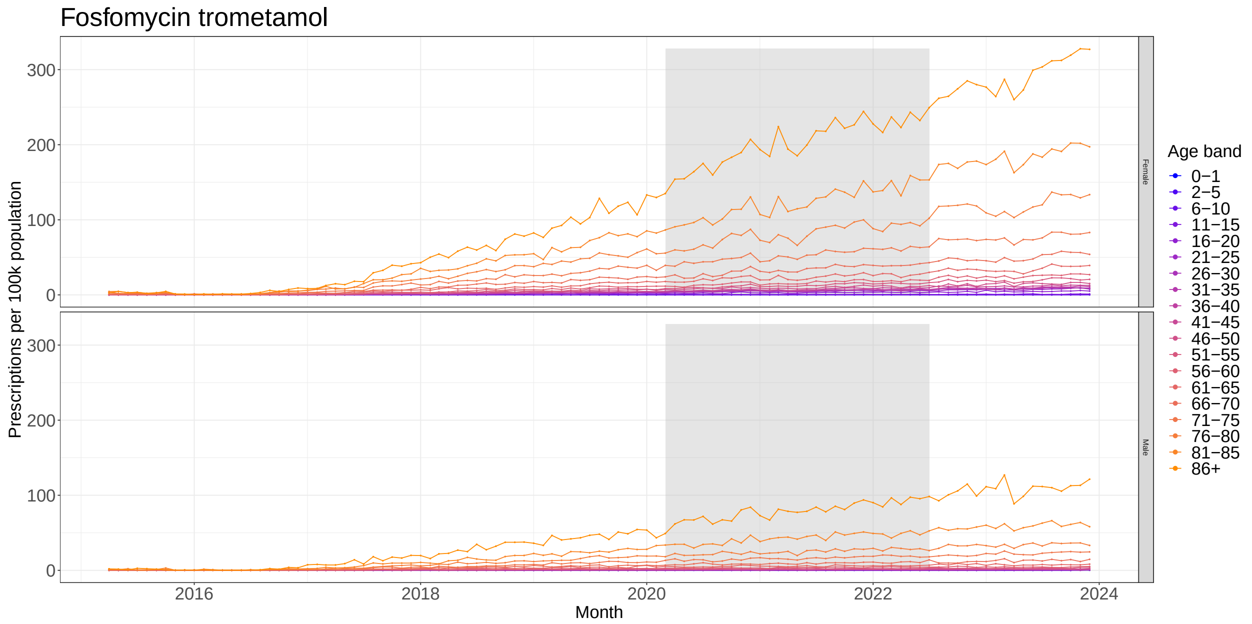


Figure S43: Prescription rate per 100’000 population for Fosfomycin trometamol . Colours indicate age groups, facets indicate sex. Grey shading indicates years of Covid-19 interventions

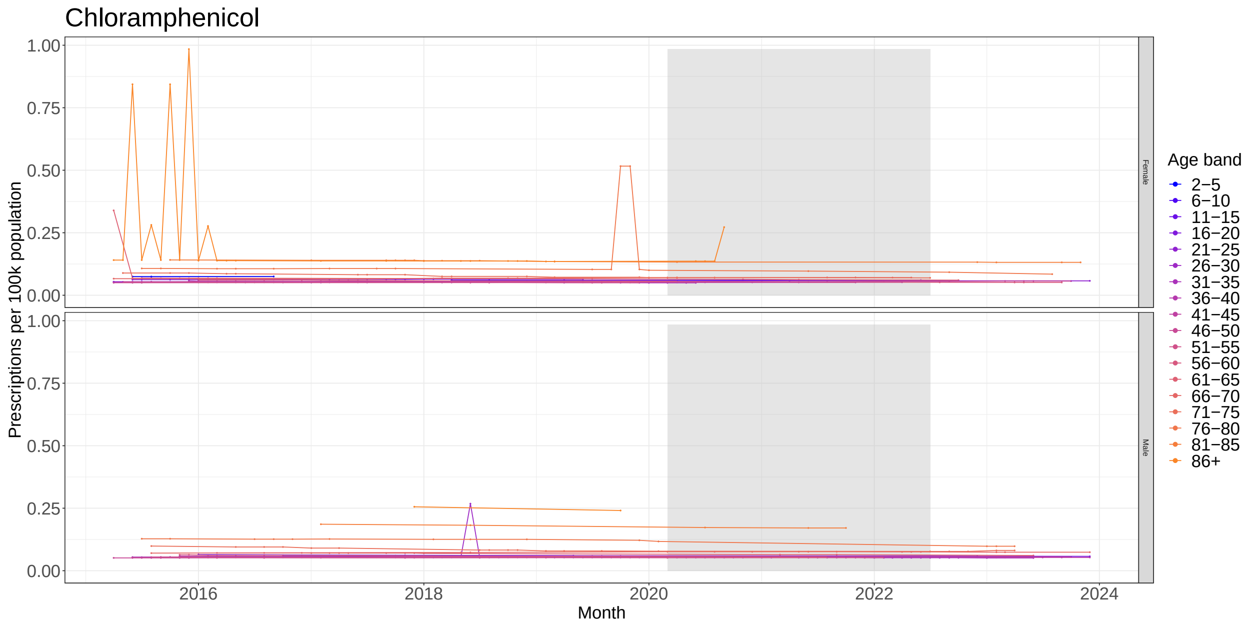


Figure S44: Prescription rate per 100'000 population for Chloramphenicol . Colours indicate age groups, facets indicate sex. Grey shading indicates years of Covid-19 interventions

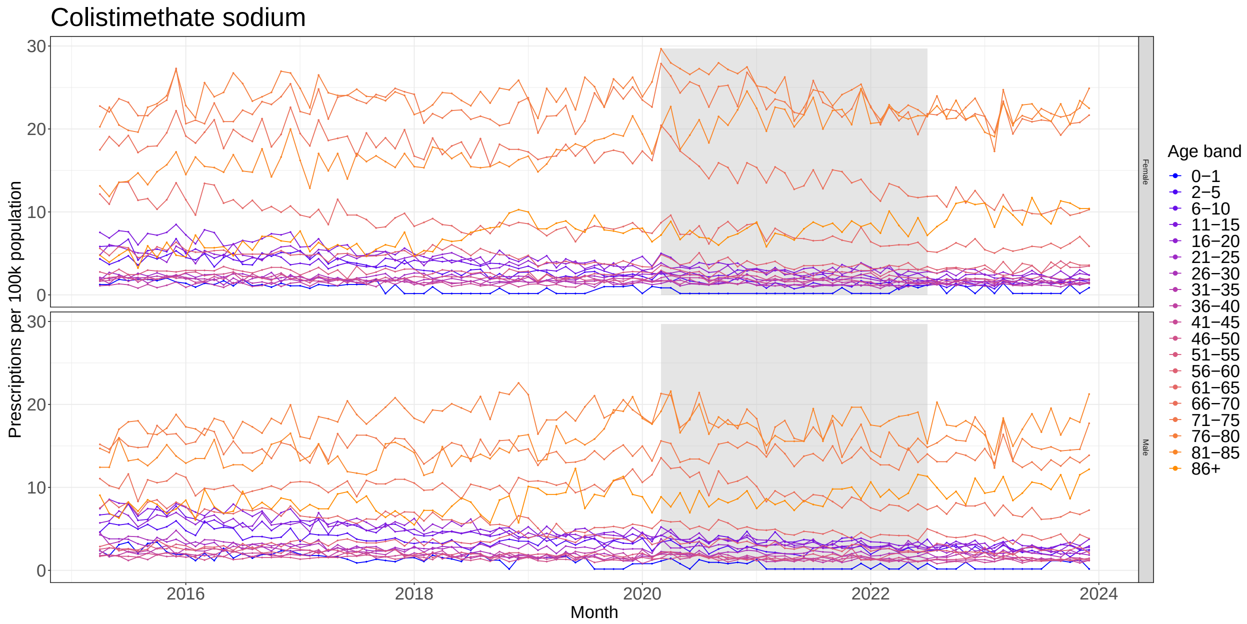


Figure S45: Prescription rate per 100'000 population for Colistimethate sodium . Colours indicate age groups, facets indicate sex. Grey shading indicates years of Covid-19 interventions

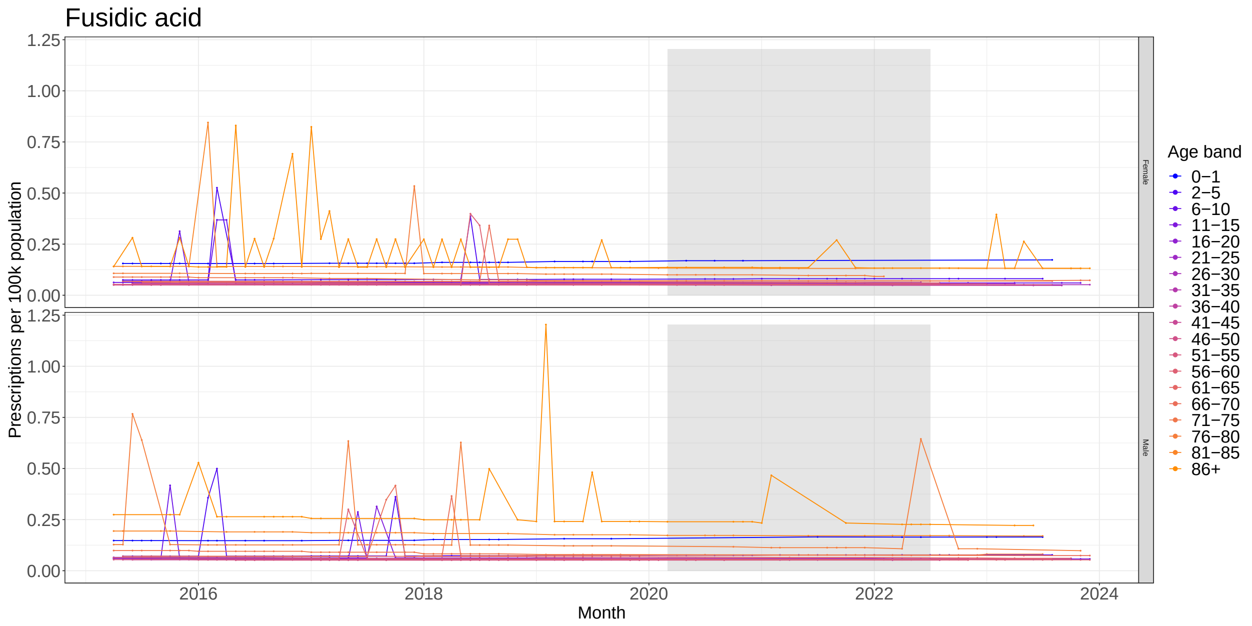


Figure S46: Prescription rate per 100'000 population for Fusidic acid . Colours indicate age groups, facets indicate sex. Grey shading indicates years of Covid-19 interventions

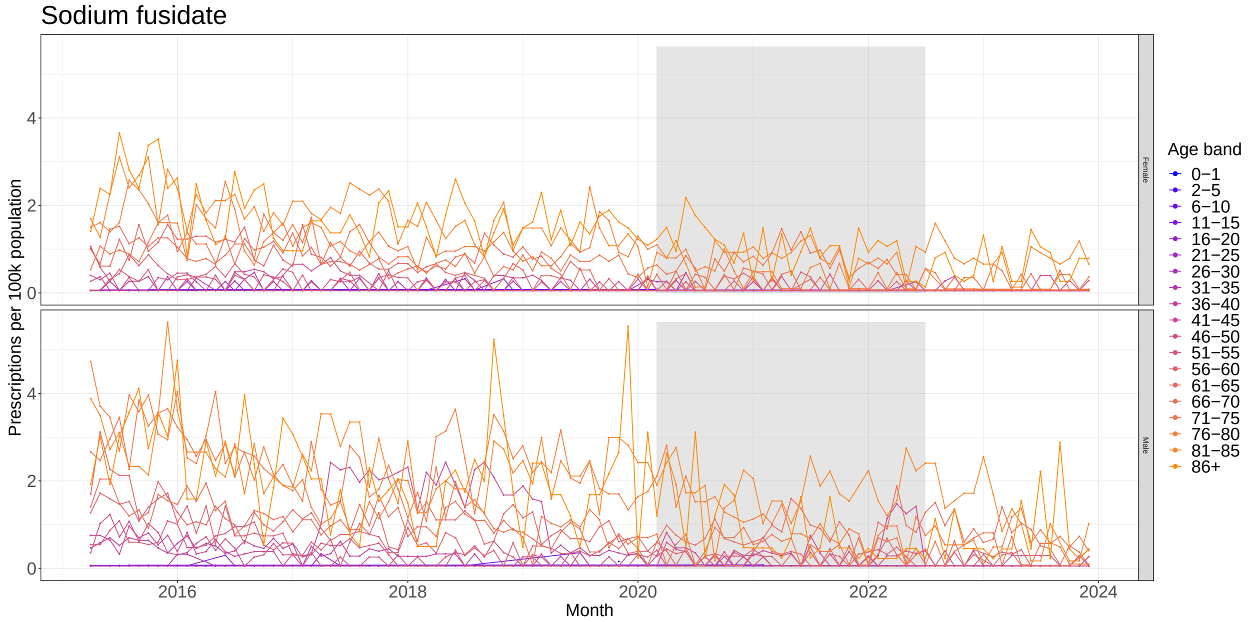


Figure S47: Prescription rate per 100'000 population for Sodium fusidate . Colours indicate age groups, facets indicate sex. Grey shading indicates years of Covid-19 interventions

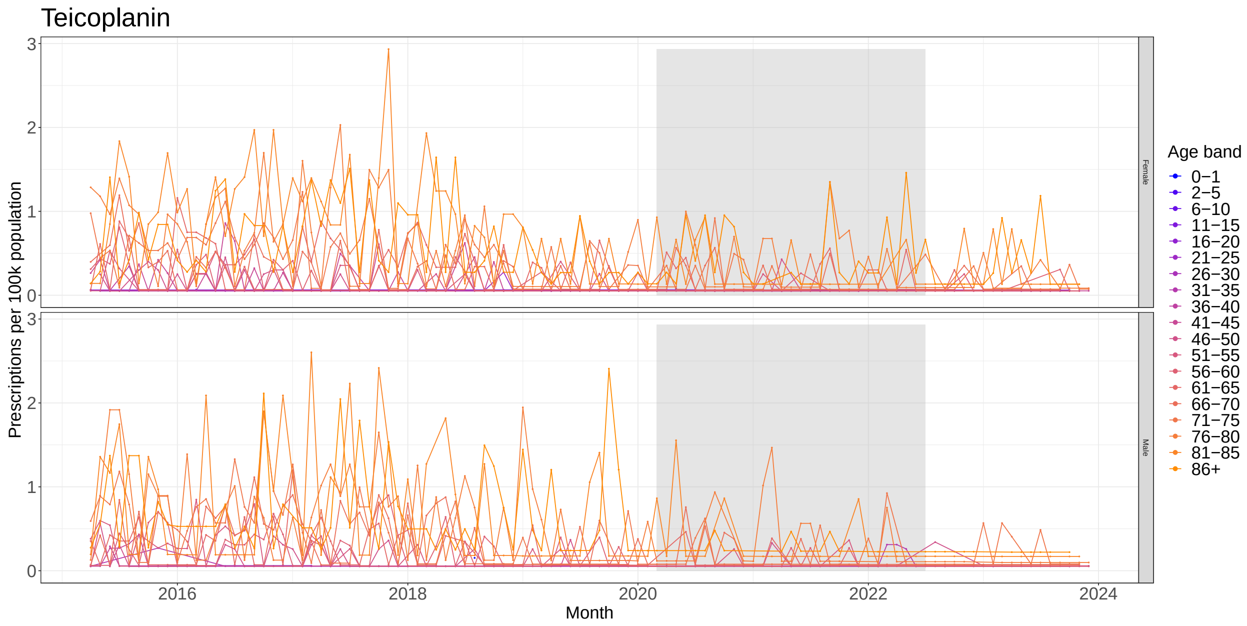


Figure S48: Prescription rate per 100’000 population for Teicoplanin . Colours indicate age groups, facets indicate sex. Grey shading indicates years of Covid-19 interventions

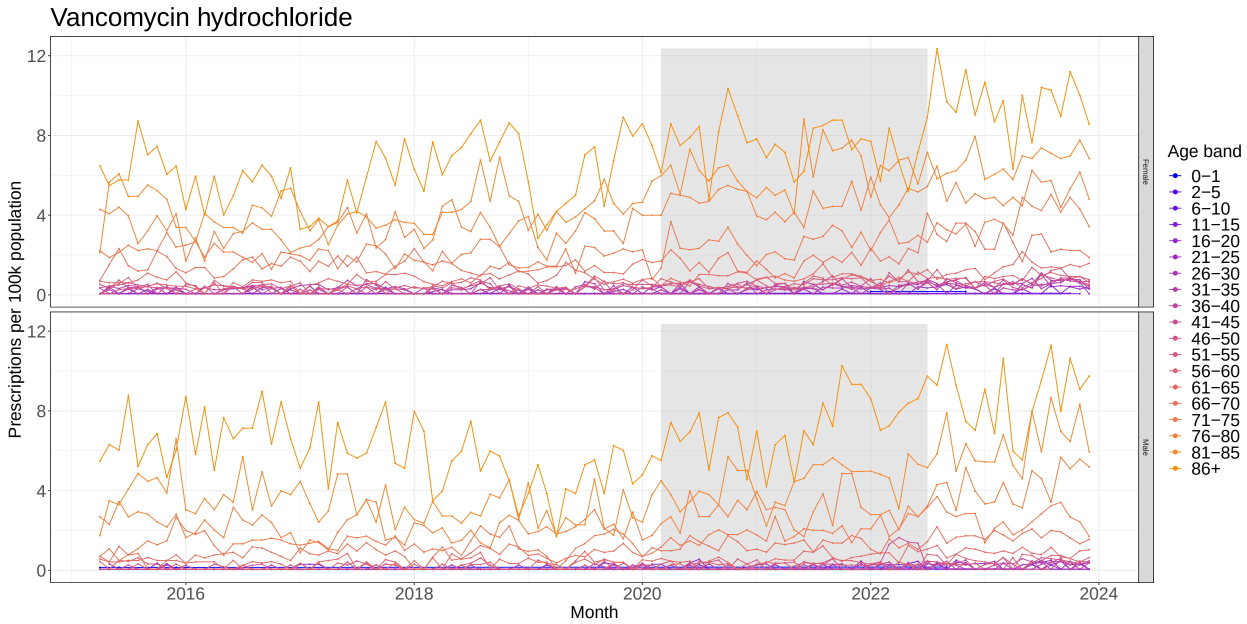


Figure S49: Prescription rate per 100’000 population for Vancomycin hydrochloride . Colours indicate age groups, facets indicate sex. Grey shading indicates years of Covid-19 interventions

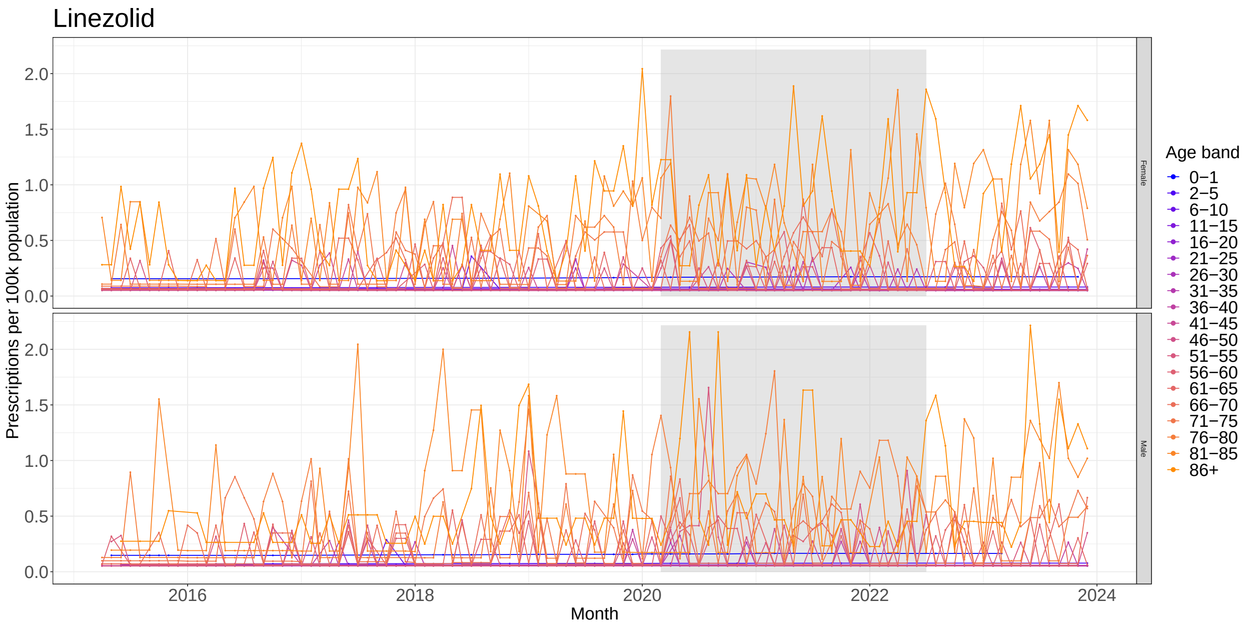


Figure S50: Prescription rate per 100'000 population for Linezolid . Colours indicate age groups, facets indicate sex. Grey shading indicates years of Covid-19 interventions

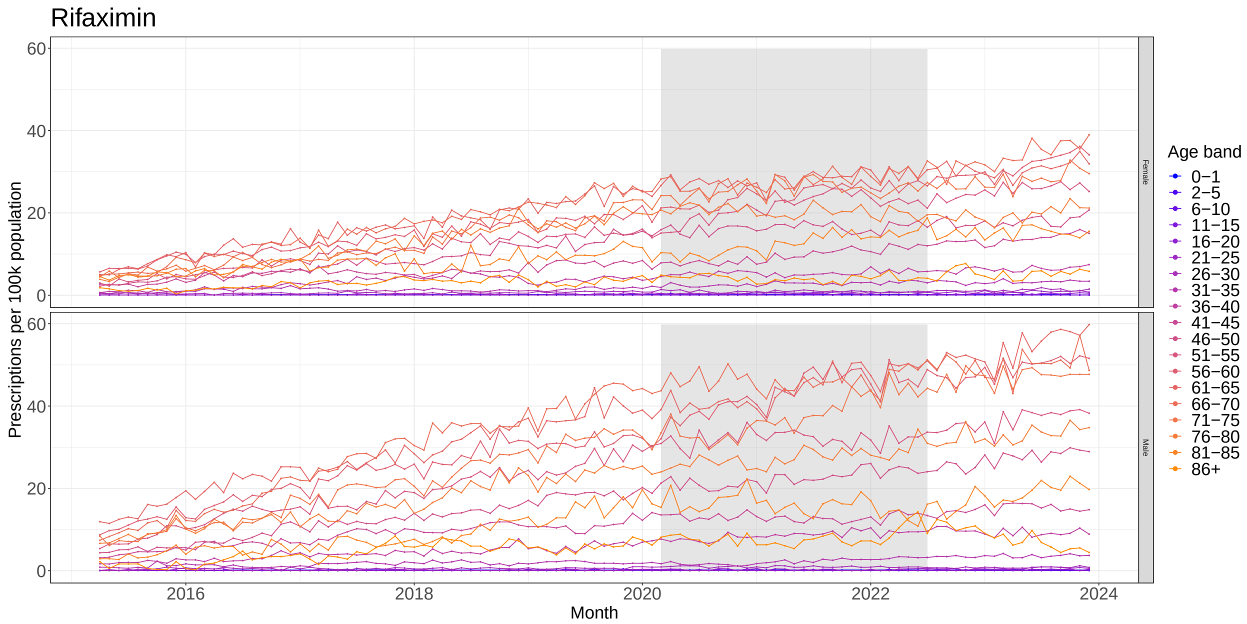


Figure S51: Prescription rate per 100’000 population for Rifaximin . Colours indicate age groups, facets indicate sex. Grey shading indicates years of Covid-19 interventions

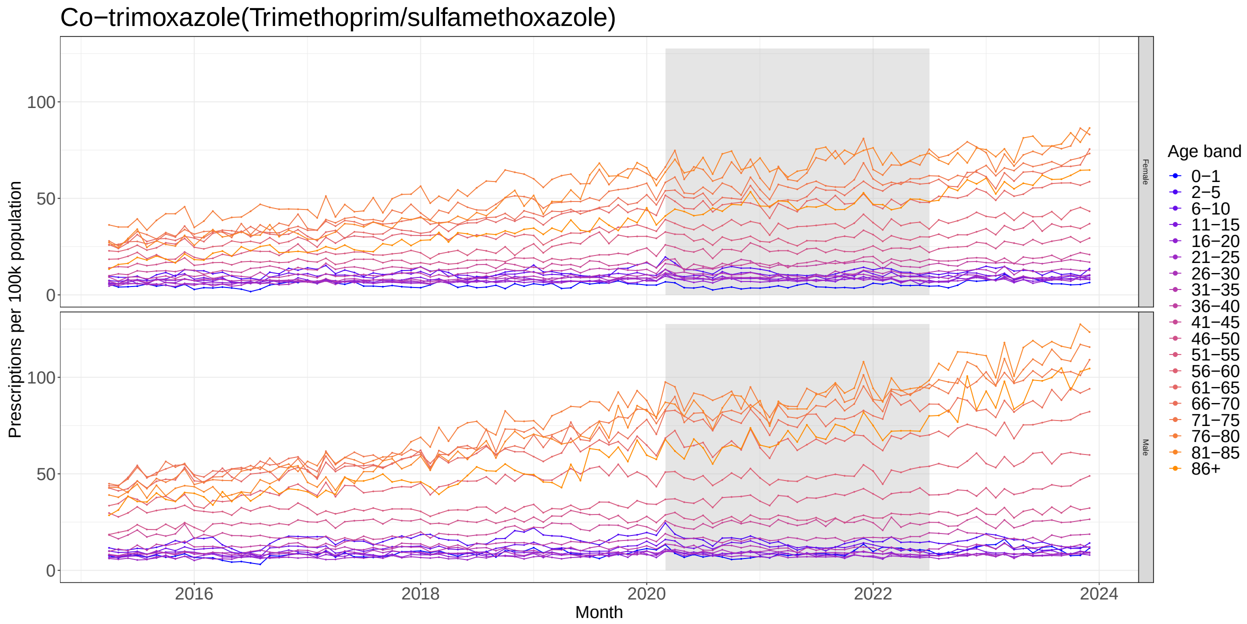


Figure S52: Prescription rate per 100’000 population for Co-trimoxazole(Trimethoprim/sulfamethoxazole) . Colours indicate age groups, facets indicate sex. Grey shading indicates years of Covid-19 interventions

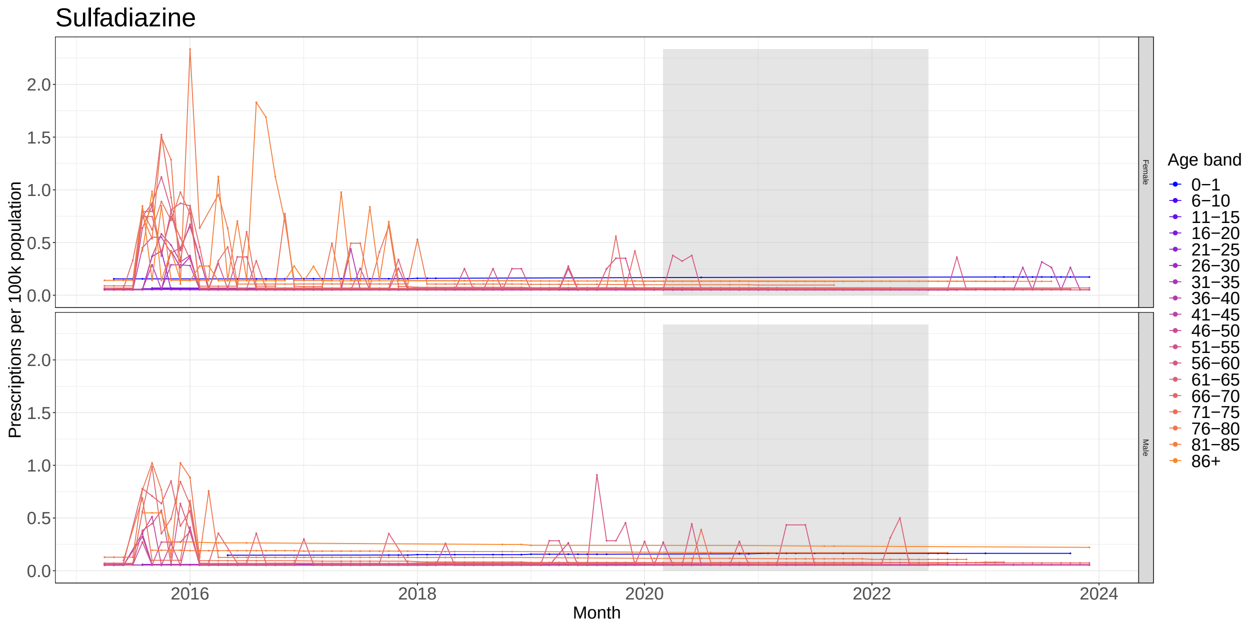


Figure S53: Prescription rate per 100’000 population for Sulfadiazine . Colours indicate age groups, facets indicate sex. Grey shading indicates years of Covid-19 interventions

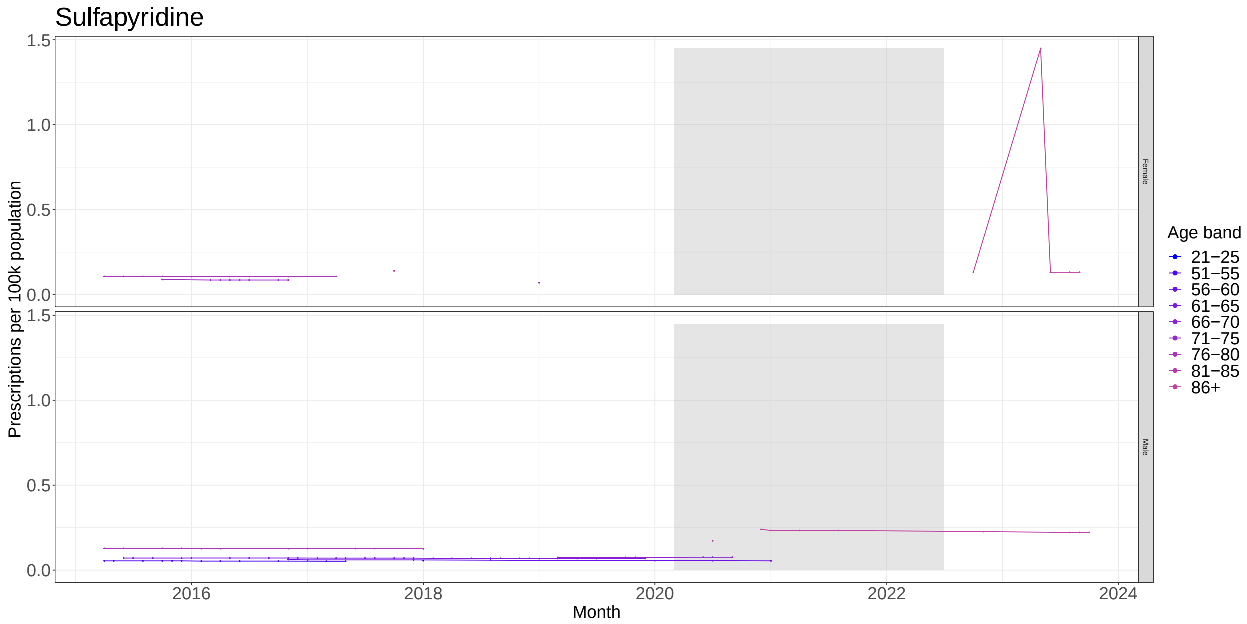


Figure S54: Prescription rate per 100’000 population for Sulfapyridine . Colours indicate age groups, facets indicate sex. Grey shading indicates years of Covid-19 interventions

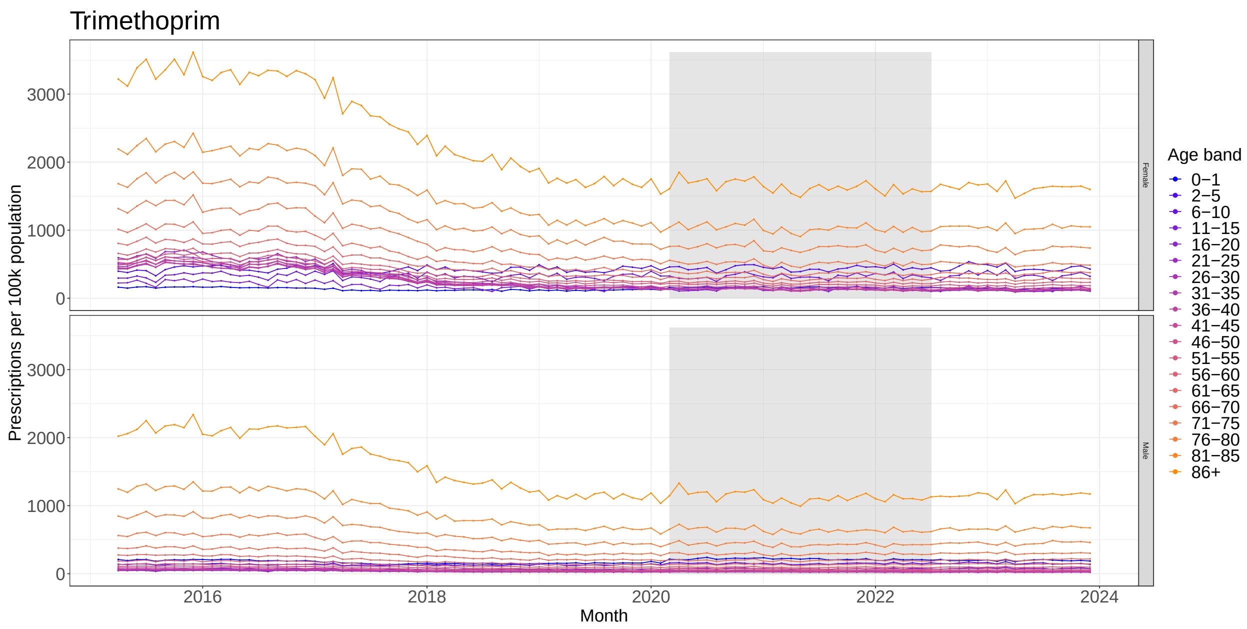


Figure S55: Prescription rate per 100'000 population for Trimethoprim . Colours indicate age groups, facets indicate sex. Grey shading indicates years of Covid-19 interventions

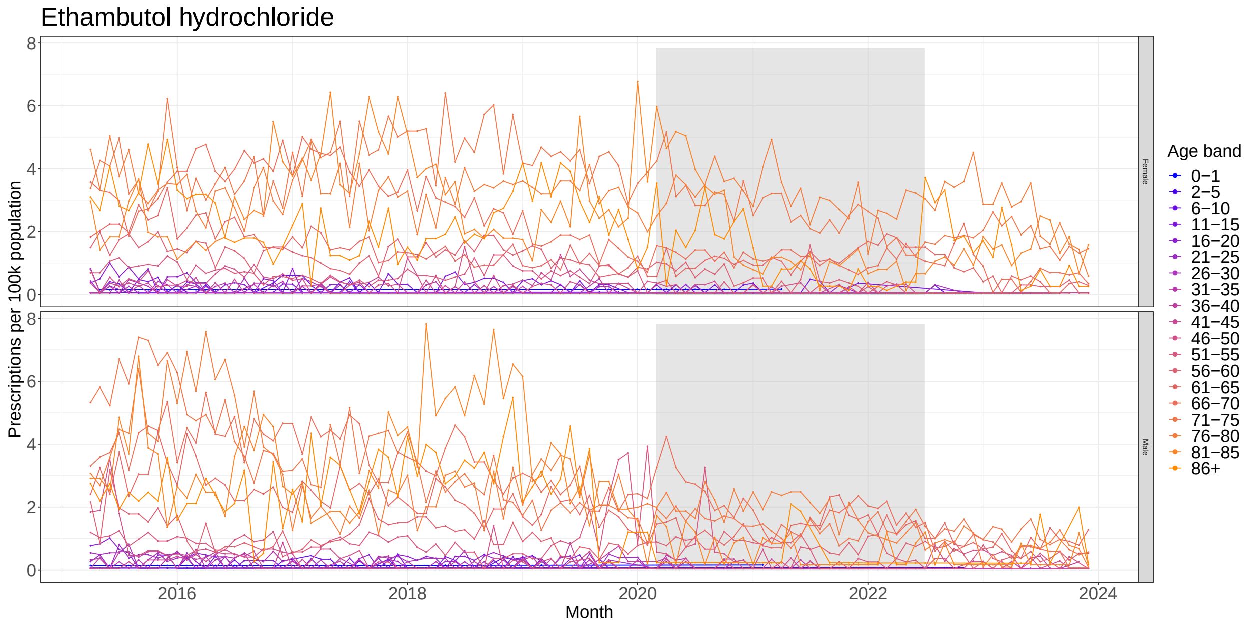


Figure S56: Prescription rate per 100'000 population for Ethambutol hydrochloride . Colours indicate age groups, facets indicate sex. Grey shading indicates years of Covid-19 interventions

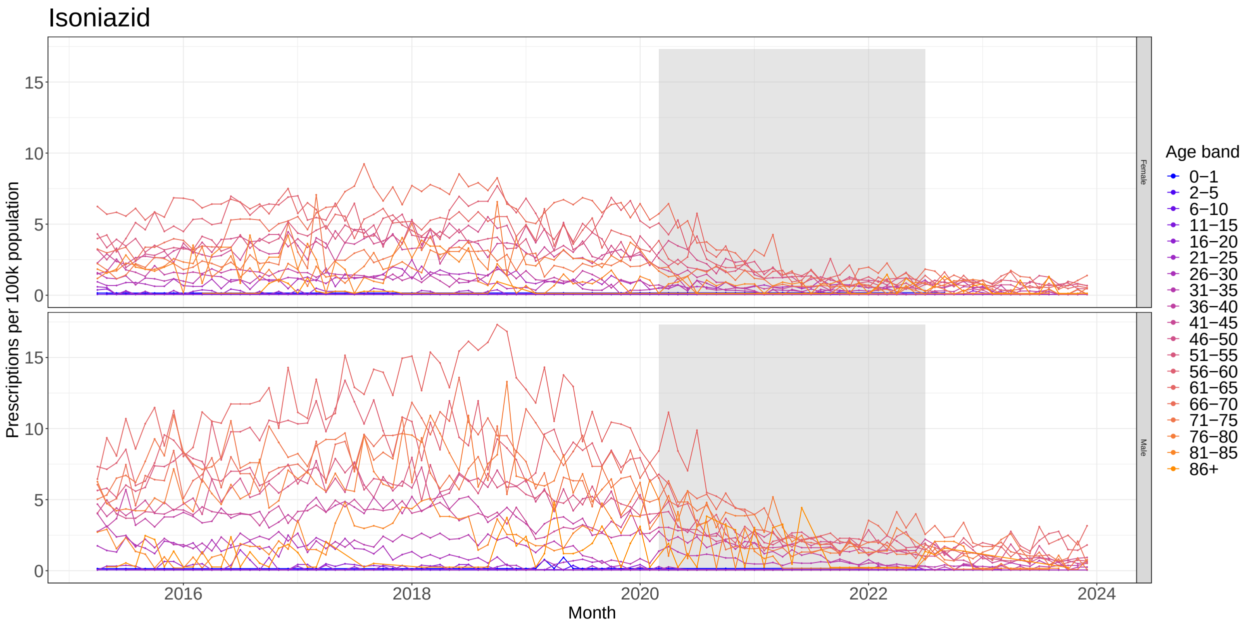


Figure S57: Prescription rate per 100'000 population for Isoniazid . Colours indicate age groups, facets indicate sex. Grey shading indicates years of Covid-19 interventions

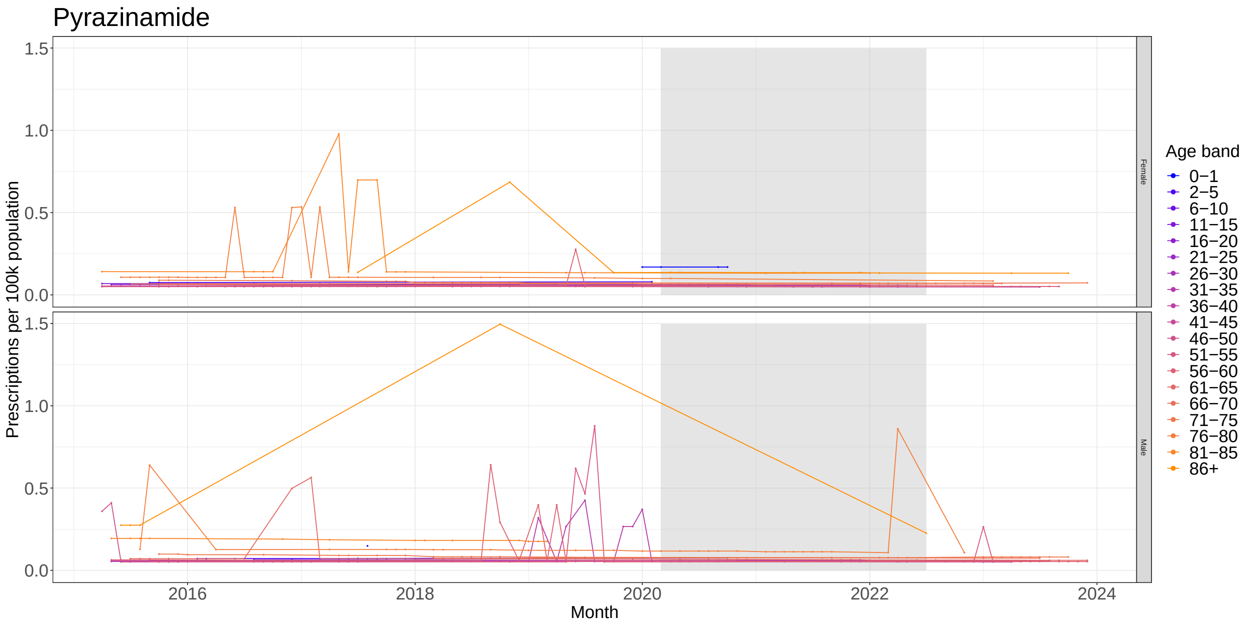


Figure S58: Prescription rate per 100'000 population for Pyrazinamide . Colours indicate age groups, facets indicate sex. Grey shading indicates years of Covid-19 interventions

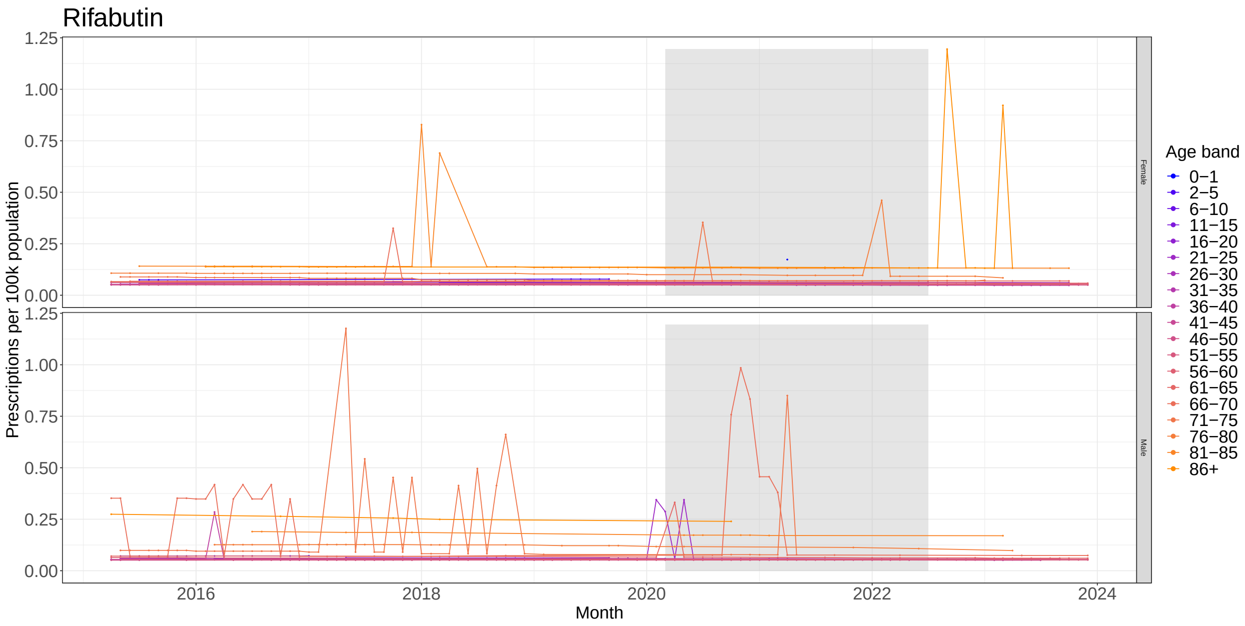


Figure S59: Prescription rate per 100'000 population for Rifabutin . Colours indicate age groups, facets indicate sex. Grey shading indicates years of Covid-19 interventions

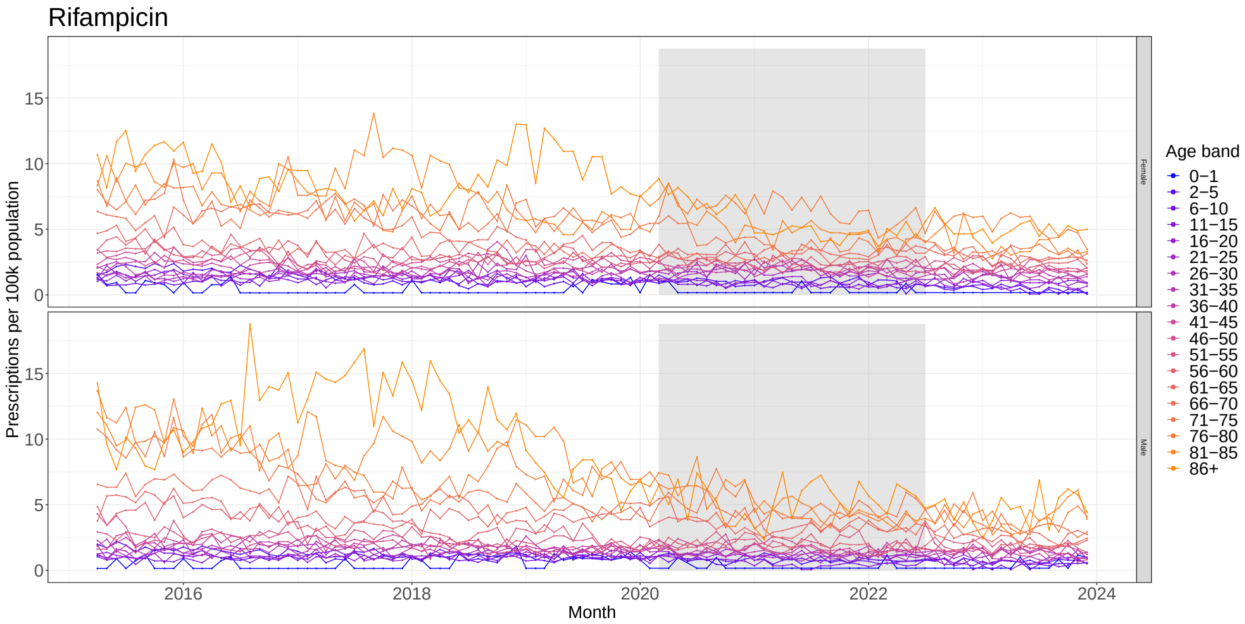


Figure S60: Prescription rate per 100'000 population for Rifampicin . Colours indicate age groups, facets indicate sex. Grey shading indicates years of Covid-19 interventions

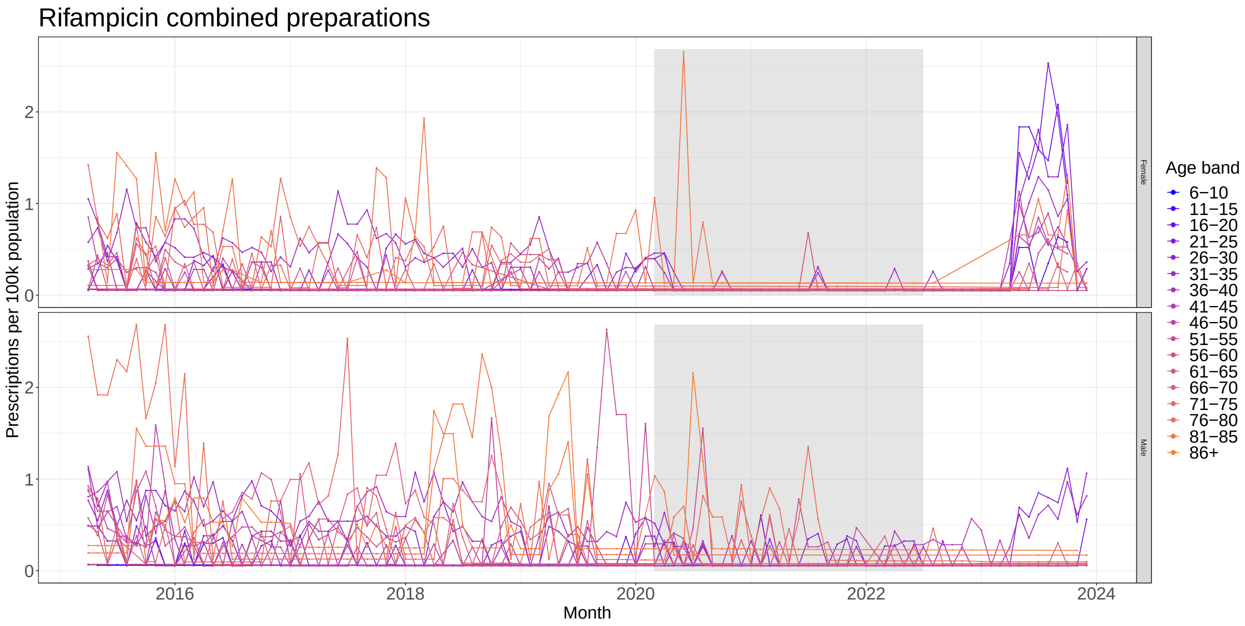


Figure S61: Prescription rate per 100’000 population for Rifampicin combined preparations . Colours indicate age groups, facets indicate sex. Grey shading indicates years of Covid-19 interventions

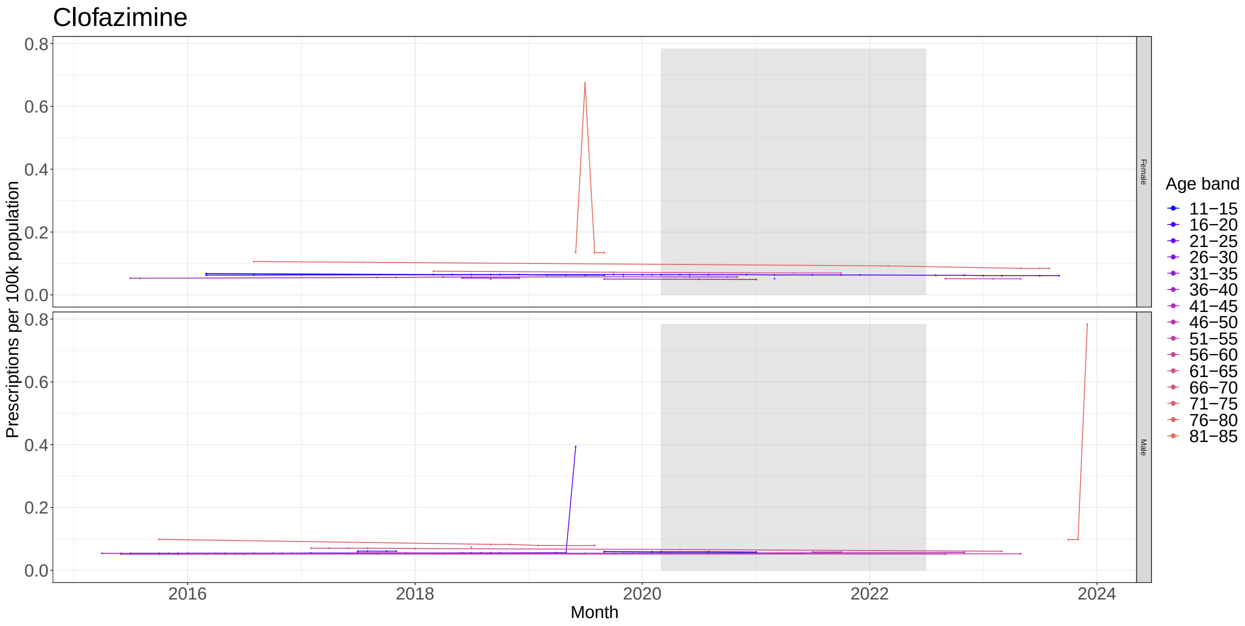


Figure S62: Prescription rate per 100’000 population for Clofazimine . Colours indicate age groups, facets indicate sex. Grey shading indicates years of Covid-19 interventions

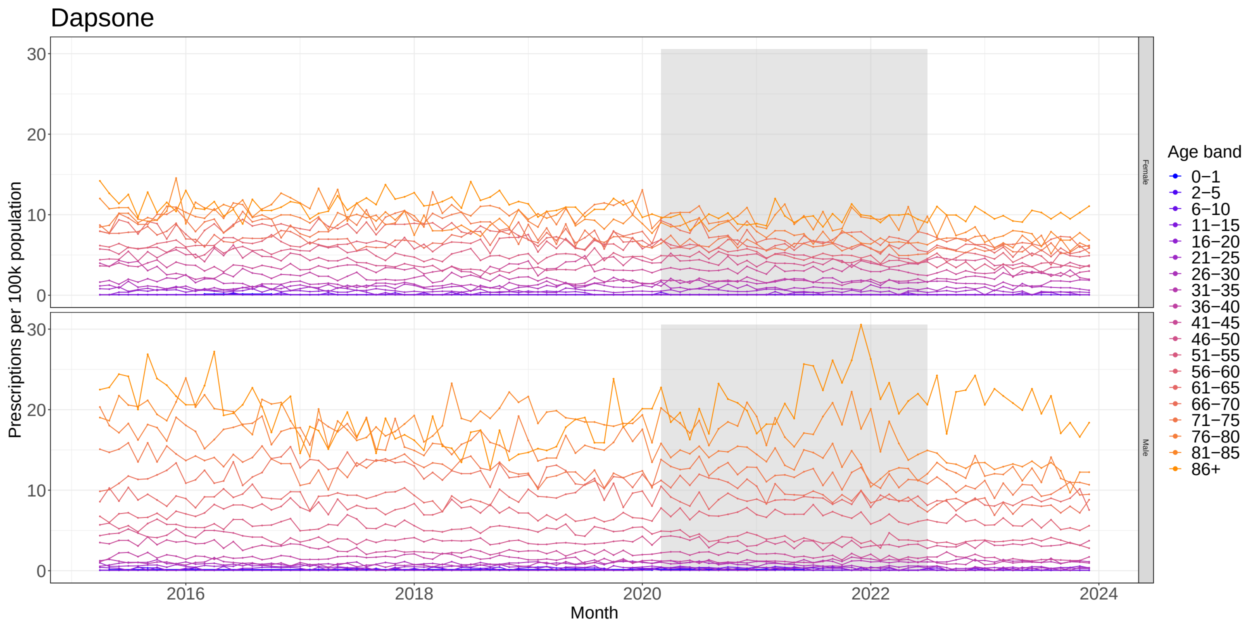


Figure S63: Prescription rate per 100'000 population for Dapsone . Colours indicate age groups, facets indicate sex. Grey shading indicates years of Covid-19 interventions

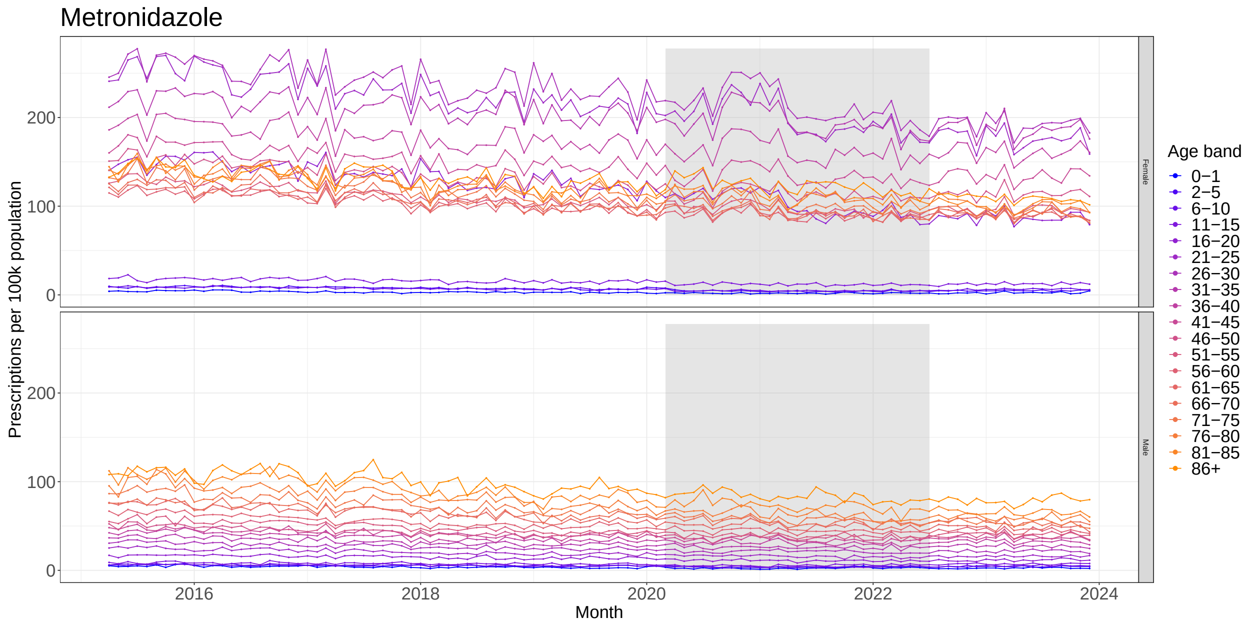


Figure S64: Prescription rate per 100'000 population for Metronidazole . Colours indicate age groups, facets indicate sex. Grey shading indicates years of Covid-19 interventions

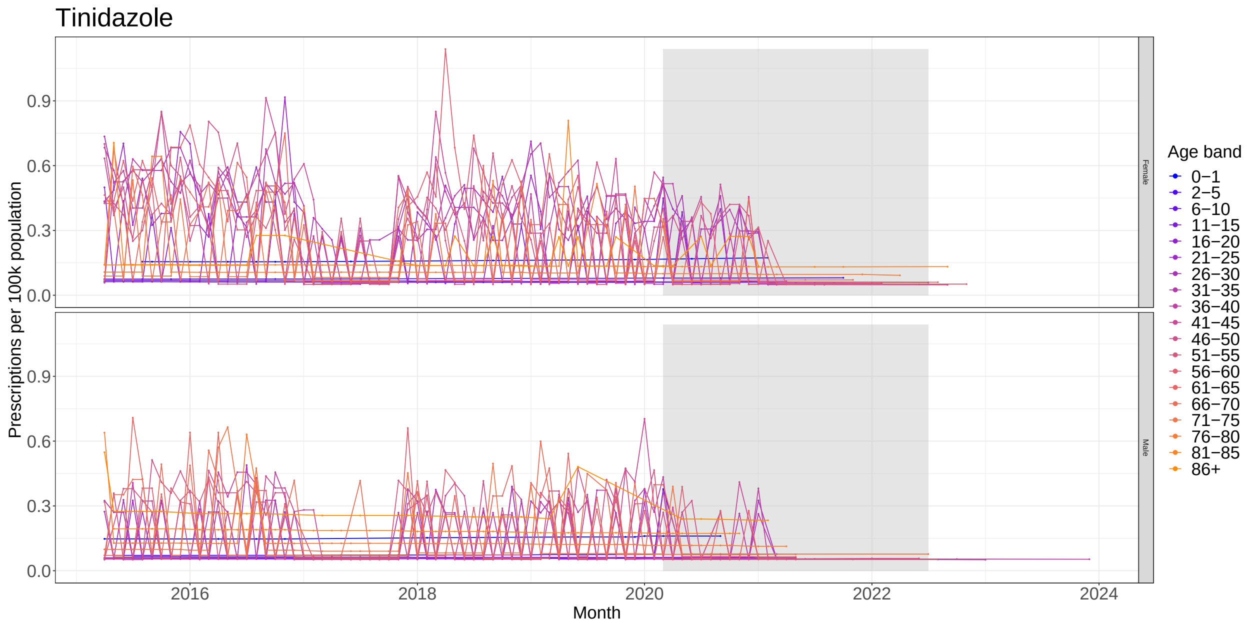


Figure S65: Prescription rate per 100'000 population for Tinidazole . Colours indicate age groups, facets indicate sex. Grey shading indicates years of Covid-19 interventions

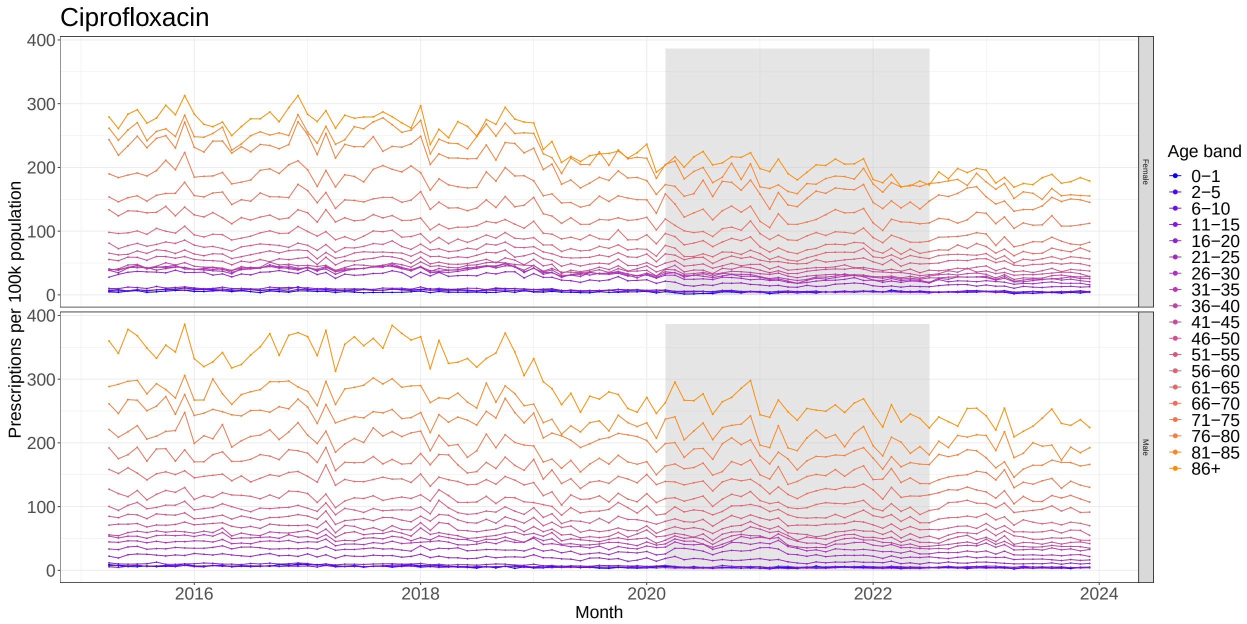


Figure S66: Prescription rate per 100'000 population for Ciprofloxacin . Colours indicate age groups, facets indicate sex. Grey shading indicates years of Covid-19 interventions

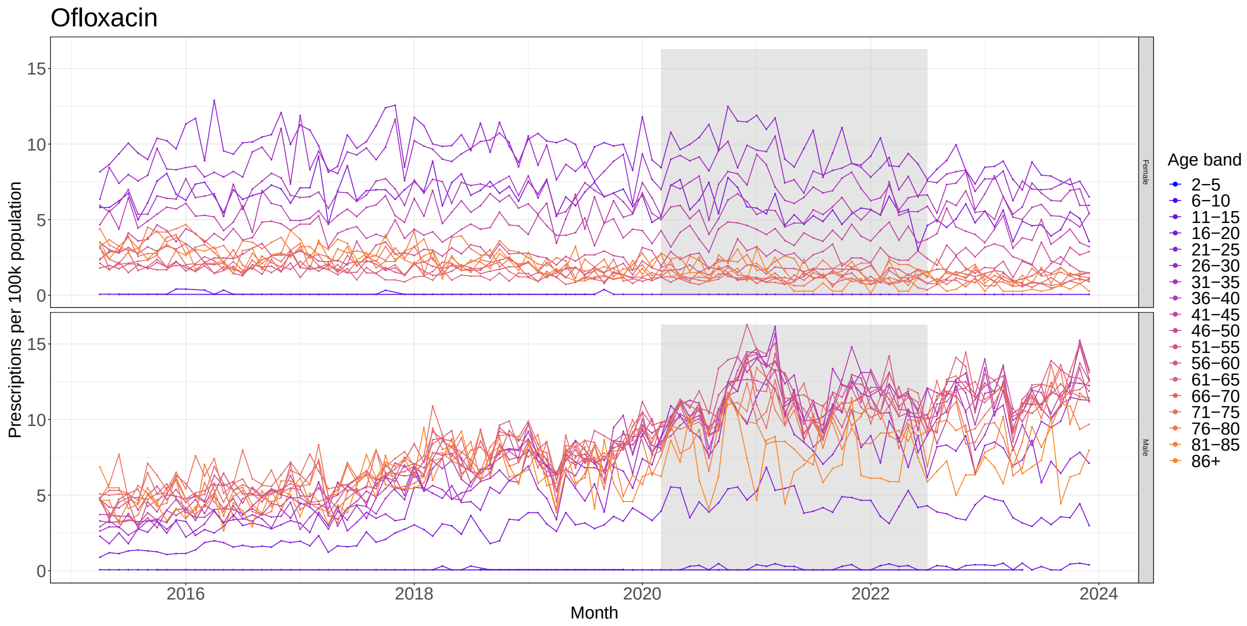


Figure S67: Prescription rate per 100’000 population for Ofloxacin . Colours indicate age groups, facets indicate sex. Grey shading indicates years of Covid-19 interventions

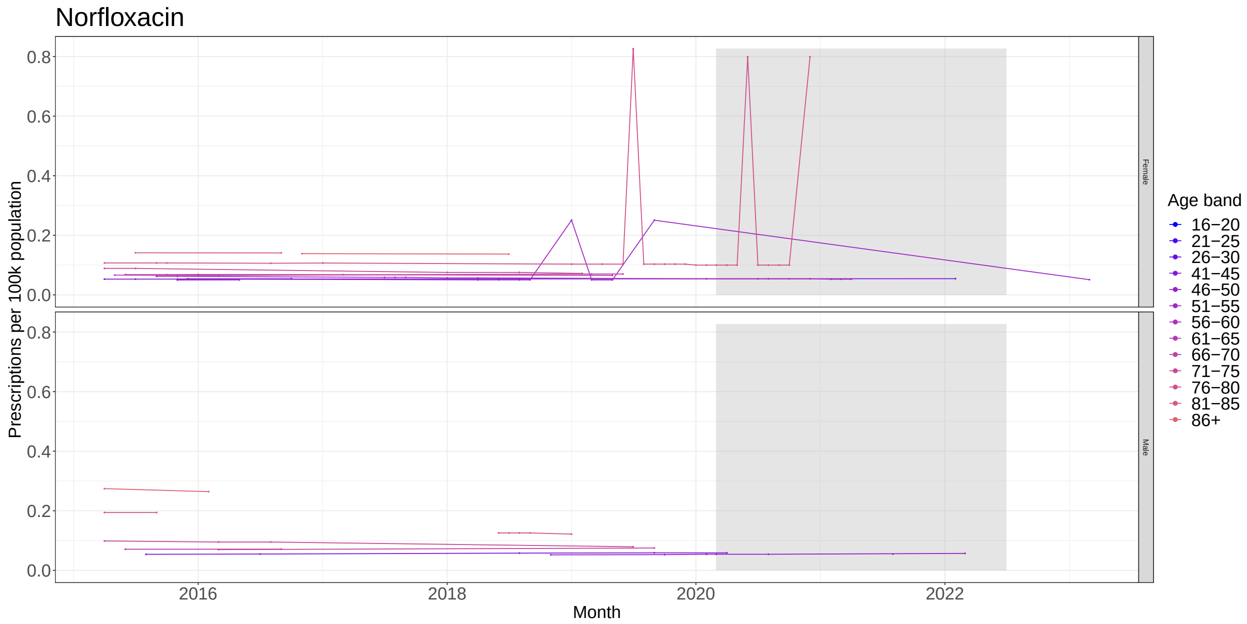


Figure S68: Prescription rate per 100’000 population for Norfloxacin . Colours indicate age groups, facets indicate sex. Grey shading indicates years of Covid-19 interventions

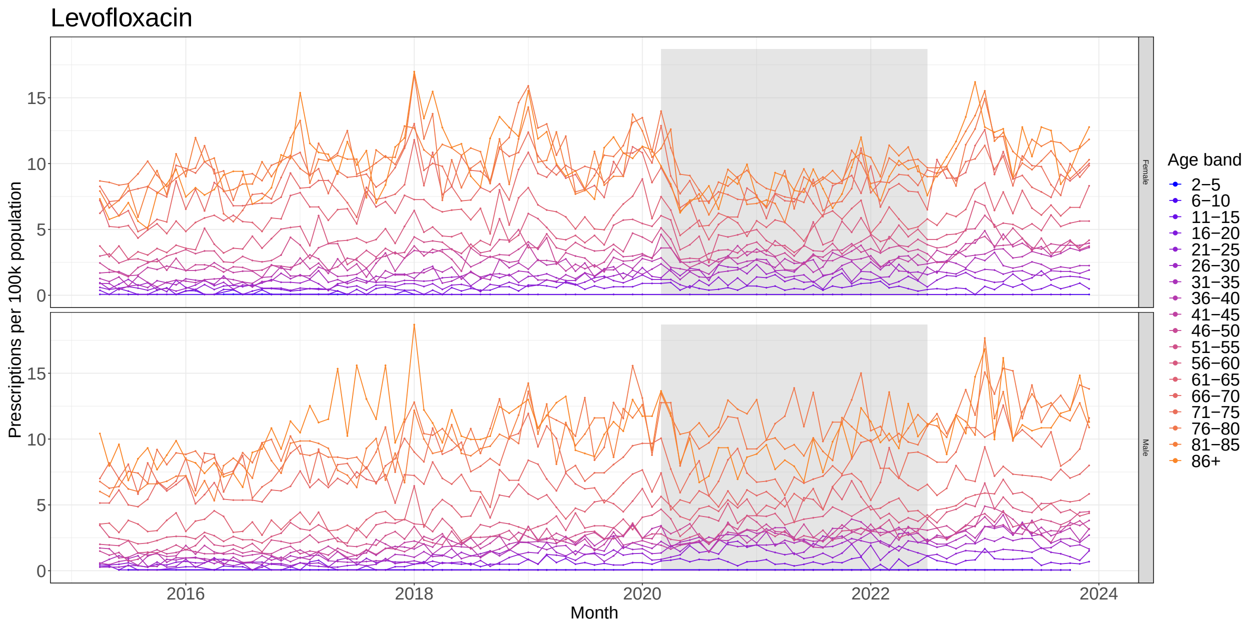


Figure S69: Prescription rate per 100'000 population for Levofloxacin . Colours indicate age groups, facets indicate sex. Grey shading indicates years of Covid-19 interventions

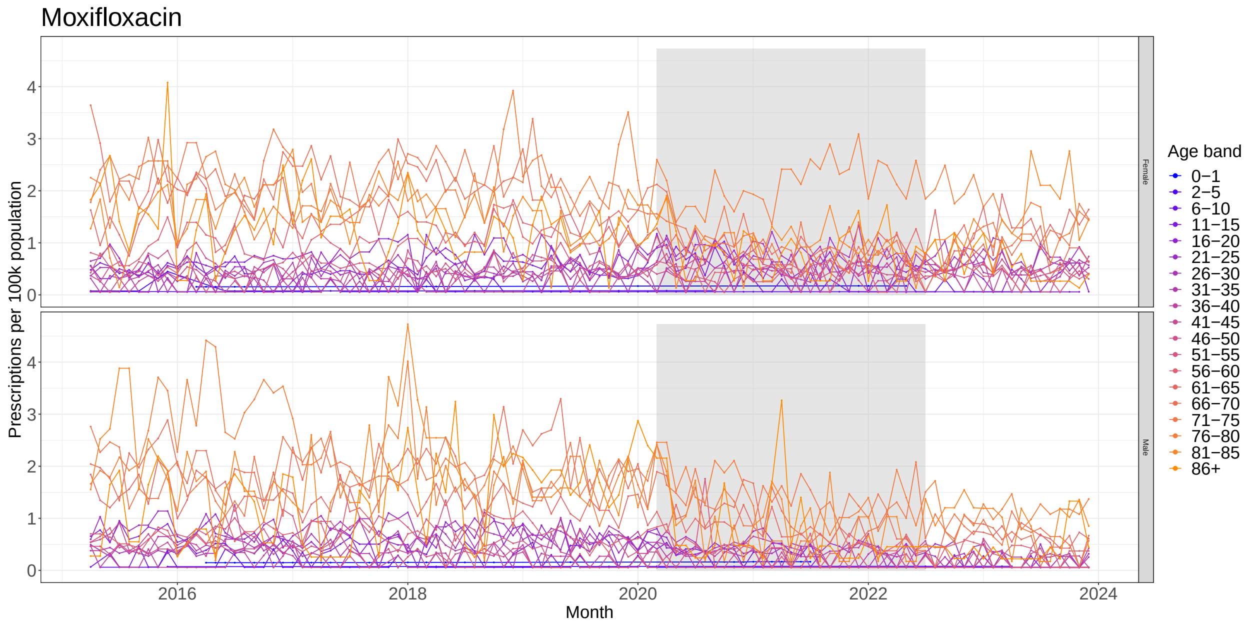


Figure S70: Prescription rate per 100'000 population for Moxifloxacin . Colours indicate age groups, facets indicate sex. Grey shading indicates years of Covid-19 interventions

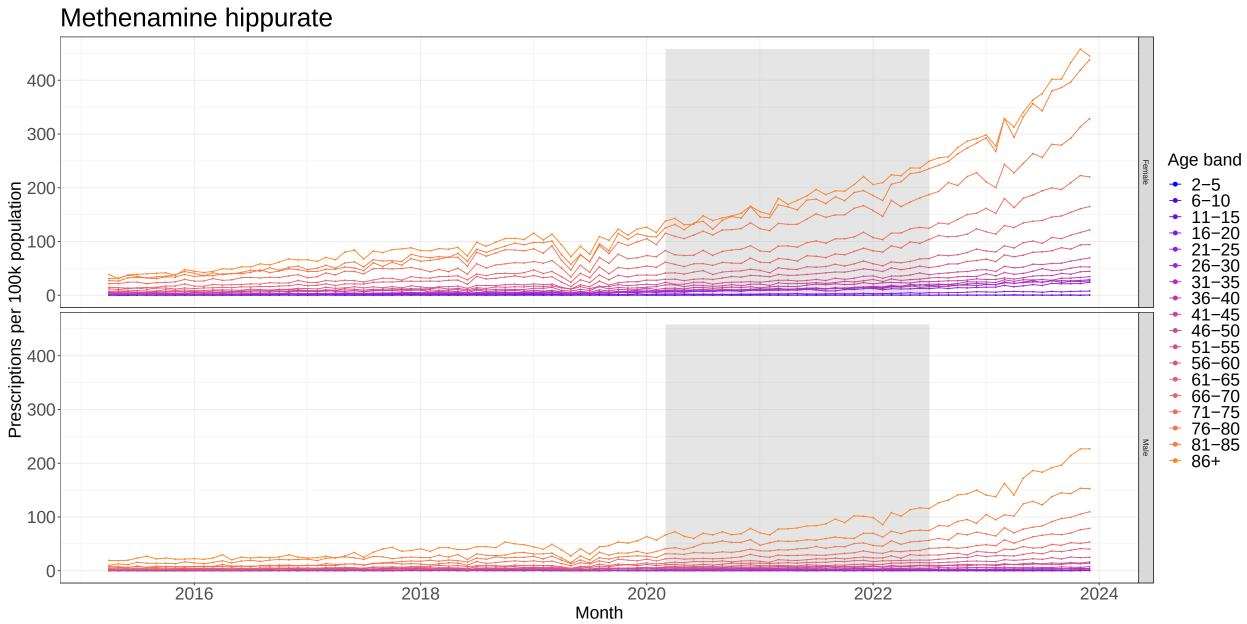


Figure S71: Prescription rate per 100’000 population for Methenamine hippurate . Colours indicate age groups, facets indicate sex. Grey shading indicates years of Covid-19 interventions

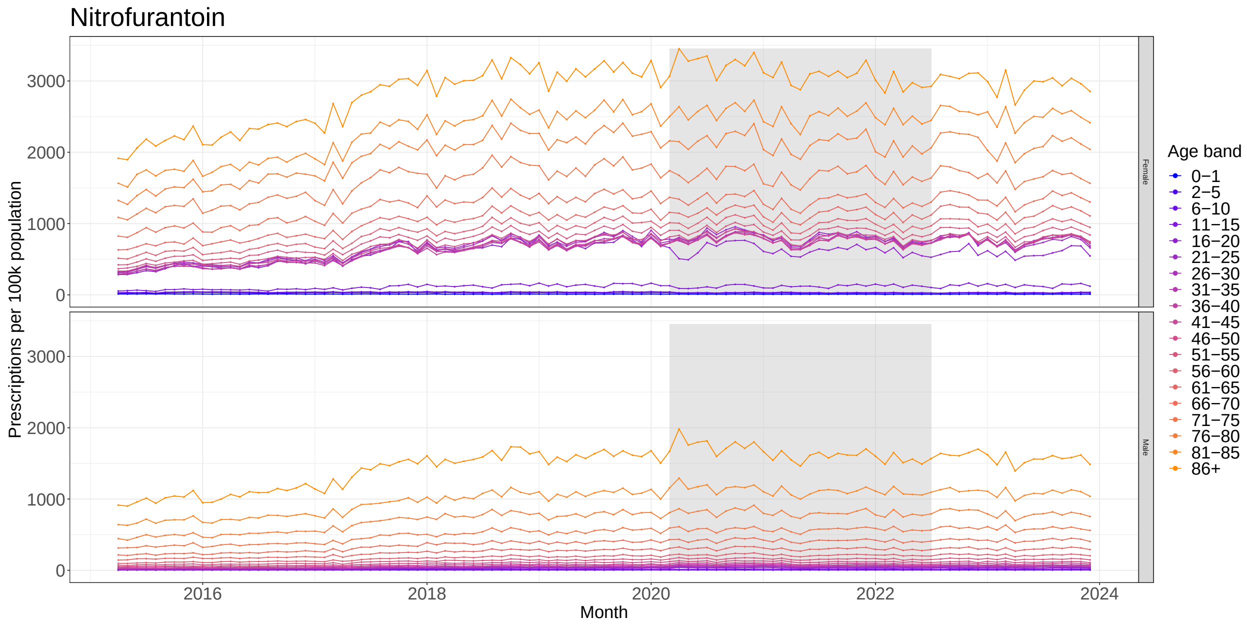


Figure S72: Prescription rate per 100’000 population for Nitrofurantoin . Colours indicate age groups, facets indicate sex. Grey shading indicates years of Covid-19 interventions

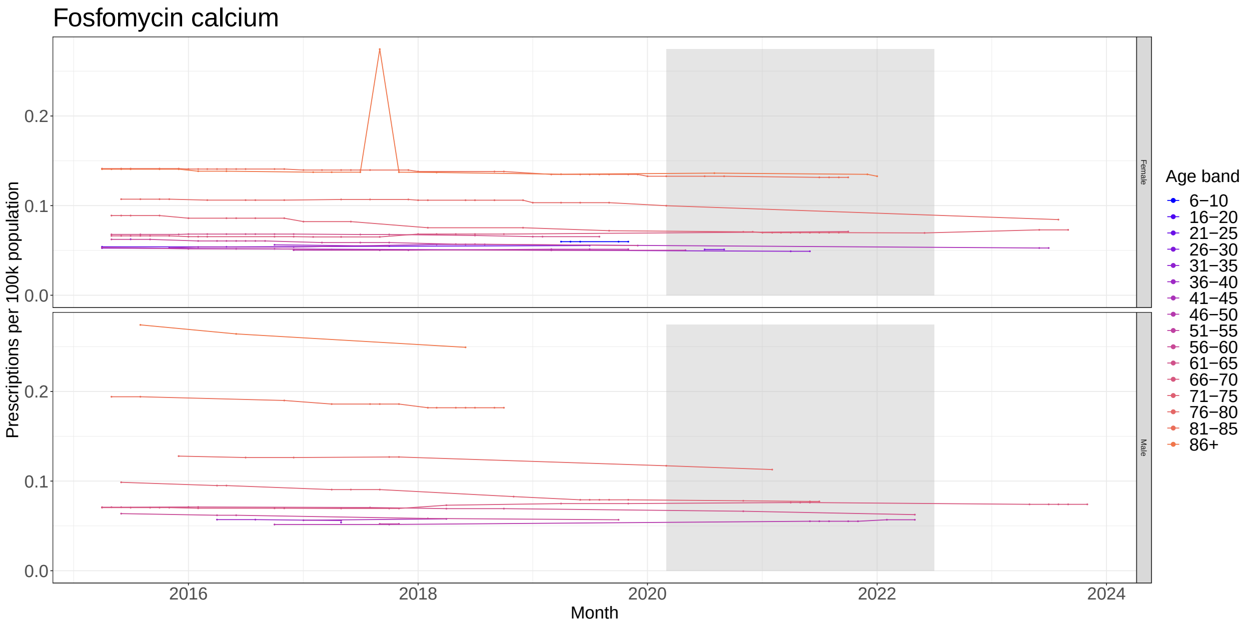


Figure S73: Prescription rate per 100'000 population for Fosfomycin calcium . Colours indicate age groups, facets indicate sex. Grey shading indicates years of Covid-19 interventions

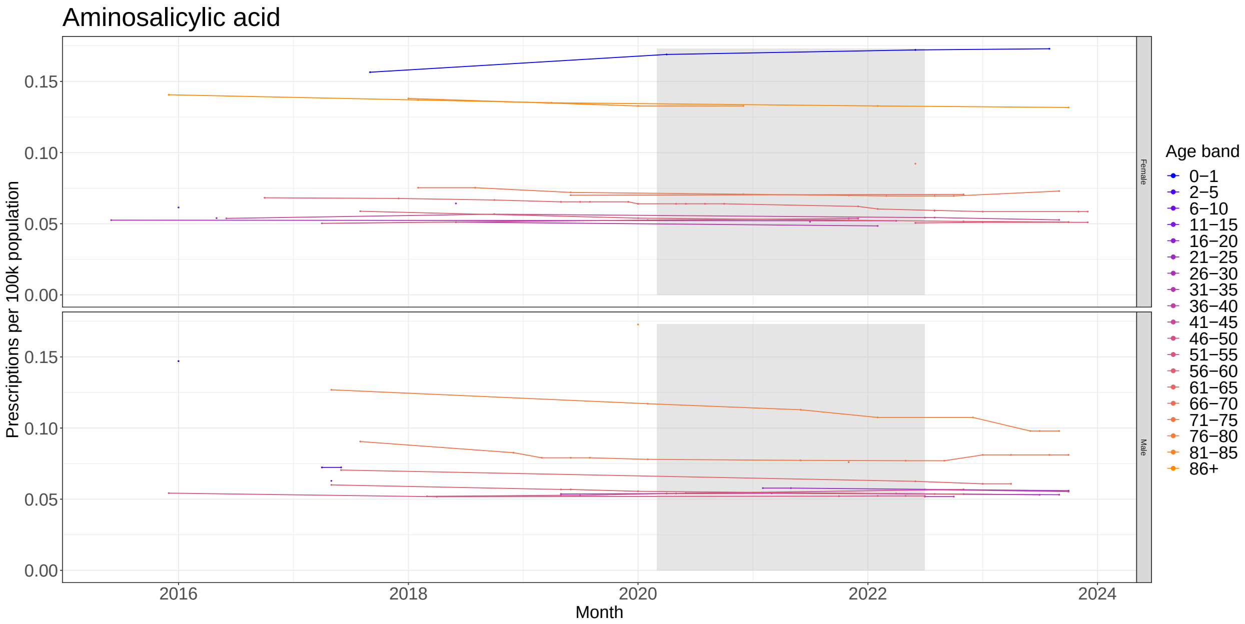


Figure S74: Prescription rate per 100'000 population for Aminosalicylic acid . Colours indicate age groups, facets indicate sex. Grey shading indicates years of Covid-19 interventions
